# Genetic Determinants of Facial Clefting: Analysis of 357 Candidate Genes Using Two National Cleft Studies from Scandinavia

**DOI:** 10.1371/journal.pone.0005385

**Published:** 2009-04-29

**Authors:** Astanand Jugessur, Min Shi, Håkon Kristian Gjessing, Rolv Terje Lie, Allen James Wilcox, Clarice Ring Weinberg, Kaare Christensen, Abee Lowman Boyles, Sandra Daack-Hirsch, Truc Nguyen Trung, Camilla Bille, Andrew Carl Lidral, Jeffrey Clark Murray

**Affiliations:** 1 Craniofacial Development, Musculoskeletal Disorders, Murdoch Childrens Research Institute, Royal Children's Hospital, Parkville, Australia; 2 Biostatistics Branch, National Institute of Environmental Health Sciences (NIEHS), Research Triangle Park, Durham, North Carolina, United States of America; 3 Department of Epidemiology (EPAM), Norwegian Institute of Public Health, Oslo, Norway; 4 Section for Epidemiology and Medical Statistics, Department of Public Health and Primary Health Care, University of Bergen, Bergen, Norway; 5 Medical Birth Registry of Norway, Norwegian Institute of Public Health, Bergen, Norway; 6 Epidemiology Branch, National Institute of Environmental Health Sciences (NIEHS), Research Triangle Park, Durham, North Carolina, United States of America; 7 Department of Epidemiology, University of Southern Denmark, Odense, Denmark; 8 College of Nursing, University of Iowa, Iowa City, Iowa, United States of America; 9 Departments of Pediatrics, Epidemiology and Biological Sciences, University of Iowa, Iowa City, Iowa, United States of America; Health Canada, Canada

## Abstract

**Background:**

Facial clefts are common birth defects with a strong genetic component. To identify fetal genetic risk factors for clefting, 1536 SNPs in 357 candidate genes were genotyped in two population-based samples from Scandinavia (Norway: 562 case-parent and 592 control-parent triads; Denmark: 235 case-parent triads).

**Methodology/Principal Findings:**

We used two complementary statistical methods, TRIMM and HAPLIN, to look for associations across these two national samples. TRIMM tests for association in each gene by using multi-SNP genotypes from case-parent triads directly without the need to infer haplotypes. HAPLIN on the other hand estimates the full haplotype distribution over a set of SNPs and estimates relative risks associated with each haplotype. For isolated cleft lip with or without cleft palate (I-CL/P), TRIMM and HAPLIN both identified significant associations with *IRF6* and *ADH1C* in both populations, but only HAPLIN found an association with *FGF12*. For isolated cleft palate (I-CP), TRIMM found associations with *ALX3*, *MKX*, and *PDGFC* in both populations, but only the association with *PDGFC* was identified by HAPLIN. In addition, HAPLIN identified an association with *ETV5* that was not detected by TRIMM.

**Conclusion/Significance:**

Strong associations with seven genes were replicated in the Scandinavian samples and our approach effectively replicated the strongest previously known association in clefting—with *IRF6*. Based on two national cleft cohorts of similar ancestry, two robust statistical methods and a large panel of SNPs in the most promising cleft candidate genes to date, this study identified a previously unknown association with clefting for *ADH1C* and provides additional candidates and analytic approaches to advance the field.

## Introduction

With an average worldwide prevalence of 1.2/1000 live births, facial clefts are the most common craniofacial birth defects and one of the most common major types of defect in humans [1]. The extensive surgical, dental and speech involvement, as well as potential psychological sequelae, underscore the importance of elucidating the causes of these complex facial defects. Analysis of familial recurrence, segregation, and concordance in twins have provided compelling evidence for a very strong genetic component to clefting [2]. In Norway, for example, the risk among first-degree relatives is approximately 40-fold higher than in the general population [3]. However, the joint contribution of genetic variants has to date accounted for only a modest fraction of the recognized etiologies [4].

The past few years have witnessed major strides in the mapping of facial cleft loci, with the list of genes rapidly expanding from the first reported association of *TGFA* [MIM 190170] with isolated cleft lip with or without cleft palate (I-CL/P) in 1989 [5] to now include *IRF6* [MIM 607199], *MSX1* [MIM 142983], *TGFB3* [MIM 190230], *FOXE1* [MIM 602617], *FGFR1* [MIM 136350], *FGFR2* [MIM 176943], *FGF8* [MIM 600483], *PDGFC* [MIM 608452], *CRISPLD2* [MIM 612434], *PVRL1* [MIM 600644], *GABRB3* [MIM 137192], *MSX2* [MIM 123101], *SATB2* [MIM 608148], *TBX10* [MIM 604648], *TBX22* [MIM 300307], *GLI2* [MIM 165230], *JAG2* [MIM 602570], *MTHFR* [MIM 607093], *RARA* [MIM 180240], *LHX8* [MIM 604425], *SKI* [MIM 164780] and *SPRY2* [MIM 602466], among the many promising candidate genes for clefts [2], [4], [6], [7], [8], [9], [10], [11], [12]. Although linkage studies have been successful in mapping a number of these key candidate genes [8], studies relying on linkage disequilibrium (LD) have grown in popularity because they can be more effective in detecting weaker associations from multiple common and low-penetrance alleles [13], [14]. Further, analyses based on haplotypes can outperform single-point analyses in which multiple SNPs in a gene are interrogated one at a time, because haplotypes can increase the overall information content at a given locus [15] and potentially capture association signals from variants that have not been directly typed [16].

We adopted this LD-based approach in the current search for fetal genetic risk factors for I-CL/P and isolated cleft palate (I-CP). A total of 1536 SNPs were selected in 357 candidate genes for facial clefts and genotyped in two population-based samples from Scandinavia (Norway and Denmark). The multi-SNP genotype data were analyzed using two complementary statistical methods, TRIMM [15] and HAPLIN [17], to detect multi-marker transmission distortion and to look for consistency in genetic associations across the two national samples.

## Methods

### Participants

A nationwide case-control study of facial clefts in Norway (1996–2001) provided 562 case-parent triads and 592 control-parent triads for analysis. The overall study design and characteristics of the study participants have been described elsewhere [18]. Of the 562 case-parent triads, 114 were I-CP and 311 were I-CL/P. An additional 69 I-CP and 166 I-CL/P triads were available from a population-based study of facial clefts in Denmark (1991–2001) [19].

### Candidate genes and SNP selection

Candidate genes for facial clefts were selected from a variety of resources, including published linkage and association studies on clefts, genome-wide scans, gene-knockout experiments in mice, studies of chromosomal rearrangements in humans, and gene expression analyses in human and mouse embryonic tissues [2], [4], [20], [21], [22], [23]. The Craniofacial and Oral Gene Expression Network (COGENE; http://hg.wustl.edu/COGENE) catalogs human gene expression changes during early embryonic development and contains expression profiles on 25 different tissues/stages in the craniofacial region of human embryos [21]. We searched the Serial Analysis of Gene Expression (SAGE) libraries to see whether a particular gene of interest is expressed in the relevant embryonic tissues at the pertinent developmental stage (weeks 5–6 for fusion of the embryonic lip; weeks 7–10 for fusion of the palatal shelves [24]).

Candidate genes were also chosen from two other global approaches for studying gene function. The first is the mouse mutagen N-ethyl-N-nitrosourea (ENU) project, which aims to characterize the functions of genes on mouse chromosome 11 (syntenic to human chromosome 17) by saturating the chromosome with point mutations [25]. The second approach is to query the Developmental Genome Anatomy Project (DGAP; http://www.bwhpathology.org/dgap/default.aspx) database using the search option ‘craniofacial: including clefts, cranial abnormalities or defects such as microcephaly’. DGAP identifies chromosomal rearrangements in patients with multiple congenital anomalies and uses these rearrangements to map and identify genes that are disrupted or dysregulated in critical stages of human development [26].

Finally, Mendelian forms of clefting provide another important avenue for identifying genes that may underlie the more common and isolated forms of clefts, especially if these syndromic forms can occasionally manifest as phenocopies of isolated clefts (e.g. Van der Woude syndrome, VWS [MIM 119300]). The Online Mendelian Inheritance in Man (OMIM; http://www.ncbi.nlm.nih.gov/Omim) maintains a catalog of human genes and genetic disorders, providing a rich resource for selecting candidate genes for clefts. Using the search query ‘cleft lip OR cleft palate’, 439 entries were retrieved from OMIM.

Using the above strategies, we compiled a list of 357 candidate genes that may contribute to facial cleft etiology (**Table S1**). To guide the selection of SNPs, the genome browser of the International HapMap Consortium (http://www.hapmap.org) was used to retrieve genotype data on these 357 candidate genes. We used genotypes from the CEPH samples of Northern and Western European ancestry for consistency with our white study populations. SNPs were also selected from additional databases: **dbSNP** (http://www.ncbi.nlm.nih.gov/projects/SNP); Japanese SNP database (JSNP; http://snp.ims.u-tokyo.ac.jp); genome browser at **UCSC** (http://genome.ucsc.edu); **CHIP** bioinformatics tools (http://snpper.chip.org/bio/); and **Seattle SNPs** (http://pga.mbt.washington.edu). The following criteria were used to prioritize SNP selection: prior evidence of an association with facial clefts; haplotype-tagging properties; minor allele frequency (MAF) of at least 10%; and a preference for coding SNPs and SNPs located in putative regulatory regions in the UTRs. SNPs with high MAFs are more likely to have arisen on ancestral chromosomes, making them more useful in assessing the degree of LD in the Norwegian and Danish populations. Intragenic and regulatory SNPs are more likely to be of functional importance, while haplotype-tagging SNPs (htSNPs) are valuable in that they use LD to extract the maximum amount of genetic information from a particular haplotype block and to possibly tag for SNPs with an unrecognized biological function.

A combination of software, including **HAPLOVIEW** (http://www.broad.mit.edu/haploview/haploview; [27]), Best Enumeration of SNP Tags (**BEST**; http://www.genomethods.org/best/index.htm; [28]), and **SNP Browser™** (Applied Biosystems; Foster City, CA), was used to evaluate MAF, inter-marker distance, as well as LD patterns and haplotype block structures for the selection of htSNPs. SNP assays were designed by Illumina (http://www.illumina.com; San Diego, CA) and a ‘design score’ was computed for each SNP using an algorithm that rigorously tests the performance of that SNP on an Illumina GoldenGate™ platform (Illumina, San Diego, CA). Approximately 10% more SNPs than the full panel of 1536 SNPs were initially submitted to allow flexibility in choosing substitute SNPs for those with poor design scores. After multiple rounds of SNP evaluations, a custom panel of 1536 SNPs was finalized for the 357 genes (**Table S2**). Genotyping was done by the US Center for Inherited Disease Research (CIDR; http://www.cidr.jhmi.edu).

### Data cleaning

The within- and between-plate genotype reproducibility rates of 96 duplicate DNA samples and three additional samples that were genotyped multiple times were used to evaluate the quality of genotyping. A SNP was deemed to have failed if fewer than 95% of the samples generated a genotype at the locus. SNPs with low minor allele frequency (MAF<1%) were also excluded due to their lack of statistical power. Deviations from HWE in control samples may indicate the presence of systematic genotyping errors, latent population substructure, or a biological effect such as natural selection [29], [30], [31]. For the **Tri**ad **M**ulti-**M**arker test [15] (TRIMM; see below), deviation from HWE (p<0.05) was used to search for assay problems directly, since the validity of TRIMM does not require SNPs to be in HWE. SNPs that showed deviation from HWE in all of the following three sample sets: parents in Norwegian case triads, parents in Norwegian control triads, and parents in Danish triads, were deemed to be problematic and three additional SNPs were removed based on this criterion.

To screen for Mendelian inconsistencies within families, we used PedCheck [32]. Families with excessive Mendelian inconsistencies are probably the result of sample switches or misidentified paternity. We retained families with fewer than 10 inconsistencies and removed eighteen families with 65 or more Mendelian inconsistencies from the analyses (there were no families with between 10 to 64 Mendelian inconsistencies).

After data cleaning, the final number of SNPs available for analysis was 1315 in 334 candidate genes on autosomal chromosomes, with one to twelve SNPs typed per gene, four SNPs on average (see **Tables S1** and **S2** for details).

### Data analysis

In this study, we investigated genes on autosomal chromosomes only. Two complementary methods, TRIMM [15] and HAPLIN [17], were used to analyze the case-parent triad genotype data. TRIMM is a robust and intuitive statistical method for association testing using multi-SNP genotype data from case-parent triads. It uses the genotypes directly, obviating the need for either a HWE assumption or haplotype phase inference. TRIMM identifies transmission distortion for sets of SNPs by comparing the genotypes of the offspring with those of the hypothetical “complement” child who would have inherited the two parental alleles not transmitted to the observed affected offspring at each locus. The LD structure is preserved in this hypothetical offspring.

The difference between the two offspring genotype vectors has an expected value of zero at each locus, under the null hypothesis of no linkage or no association with the disease locus under study. Statistical significance of the test is evaluated by randomly permuting the case-versus-complement labels for the pair of offspring from each family, i.e. randomizing the sign of the difference vector. In our analysis, we used the sum_logP test, which is constructed to optimize over scenarios where risk depends on a single studied SNP and also over scenarios where risk instead depends on a multi-SNP susceptibility haplotype. TRIMM can make full use of individuals with sporadic missing SNP data and has no limitation on the number of SNPs it can handle. All genotyped SNPs in a gene were thus used in the analyses.

HAPLIN (version 2.5) infers haplotypes from the unphased family genotype data when possible, using the Expectation Maximization (EM) algorithm. It estimates the disease relative risk for haplotypes that have a frequency high enough to be detected. The overall significance of the haplotypes is evaluated by a likelihood ratio test, or alternatively, a score test. HAPLIN can distinguish between single and double dose effects of haplotypes, but to reduce the number of parameters needed to be estimated in a multi-SNP scan, haplotype effects were assumed multiplicative in our analyses. Also, haplotypes with population frequency less than 1% were removed from the analyses.

A typical observation when constructing haplotypes with more than four SNPs is that the population LD structure dictates a large number of rare haplotypes, complicating the estimation. In our data, many of the genes with more than four SNPs displayed low LD between the SNPs at the outer ends. Using window lengths of more than four SNPs would thus generate many rare and perhaps irrelevant haplotypes. For the I-CL/P cleft category, we restricted HAPLIN to use four SNPs at a time with a sliding, overlapping window approach to cover all SNPs in a gene. Because considerably fewer cases were available for the analysis of I-CP, we used only three SNPs in the sliding-window for this cleft category. Within a gene, this produces a score test p-value for each window, and the smallest p-value was chosen. To adjust this p-value for within-gene multiple testing, the principle of ‘seemingly unrelated estimation’ [33] was used. The individual (family) score contributions for each window were saved and their between-window correlations were used to correct the p-value.

We plotted the p-values from TRIMM and HAPLIN analyses using a Schweder-Spjøtvoll plot [34], which is a simple graphical procedure for the simultaneous evaluations of many tests. If none of the genes are truly associated with facial clefts, the p-values would fall along the straight sloping line. Conversely, the p-values would deviate from this line in the presence of genes that are truly associated with the disorder. Statistical analyses were carried out on the Norwegian and Danish samples separately, and p-values from these two sources were combined using Fisher's method [35]. Fisher-combined p-values can be further corrected for multiple testing using the Bonferroni correction.

To test for within-gene differences in genotype frequency across the two national samples, we used all the case-parents in each triad. The test is based on computing the chi-squared value for each SNP in a given gene, followed by aggregating these values to obtain the within-gene sum chi-squared value. These were then converted to p-values using a permutation test with 1000 permutations (implying that the smallest p-value estimable is 0.001). The permutations permute the nationality label of each individual over all SNPs in a gene simultaneously, thus compensating for LD.

All analyses by TRIMM and HAPLIN were restricted to isolated clefts only, and were performed separately for the two major categories of clefts—I-CL/P and I-CP. Software for implementing TRIMM and HAPLIN are available for the ***R*** [36] computing environment from our web sites (TRIMM: http://www.niehs.nih.gov/research/atniehs/labs/bb/staff/weinberg/index.cfm#downloads; HAPLIN: http://www.uib.no/smis/gjessing/genetics/software/haplin).

### Ethics approval

Approval for this study was obtained from the Norwegian Data Inspectorate, the Regional Committee on Research Ethics for Western Norway, and the respective Institutional Review Boards of the US National Institute of Environmental Health Sciences (NIH/NIEHS) and the University of Iowa. For the Danish facial clefts study, approval was obtained from The Danish National Committee on Biomedical Research Ethics. Clinicopathological information from all participating families and biologic specimens for DNA extraction were obtained with the written informed consent of the mothers and fathers, and all aspects of this research were in compliance with the tenets of the *Declaration of Helsinki* for human research (http://www.wma.net).

## Results

This study was designed with the specific aim of identifying consistent genetic associations across two population-based cleft studies in Scandinavia. We genotyped 1536 SNPs in 357 candidate genes that were selected *a priori* for their potential roles in clefting. We observed a high duplicate reproducibility rate (99.98%), high Mendelian consistency rate (99.93%), and a low rate of missing genotypes (0.55%) in the data. After removing SNPs with more than 10 Mendelian errors and those with significant deviations from HWE (p<0.05), the final number of SNPs analyzed was 1315, representing a total of 334 candidate genes on autosomal chromosomes (**Tables S1** **and** **S2**).

**Tables S3** **,** **S4** **,** **S5** **,** **S6** summarize the results of the TRIMM and HAPLIN analyses by cleft type (I-CL/P vs. I-CP) and study population (Norway vs. Denmark). The corresponding Schweder-Spjøtvoll plots of p-values are provided in **Figures 1** and **2**. For I-CL/P, HAPLIN and TRIMM identified strong associations with *IRF6* and *ADH1C* [MIM 103730] in both the Norwegian and Danish sample. In addition to these, HAPLIN identified *FGF12* [MIM 605802] in both populations. For I-CP, HAPLIN identified strong associations with *PDGFC* and *ETV5* [MIM 601600] in both populations. While TRIMM confirmed the association with *PDGFC*, it also identified additional associations with *ALX3* [MIM 606014] and *MKX* (MIM entry not available for this gene). The quantile-quantile (QQ) plots of Fisher-combined p-values from the TRIMM and HAPLIN analyses in the two samples are shown in **Figure 3**. Compared with the QQ plot for I-CP, the QQ plot for I-CL/P displays an excess of small p-values.

**Figure 1:**
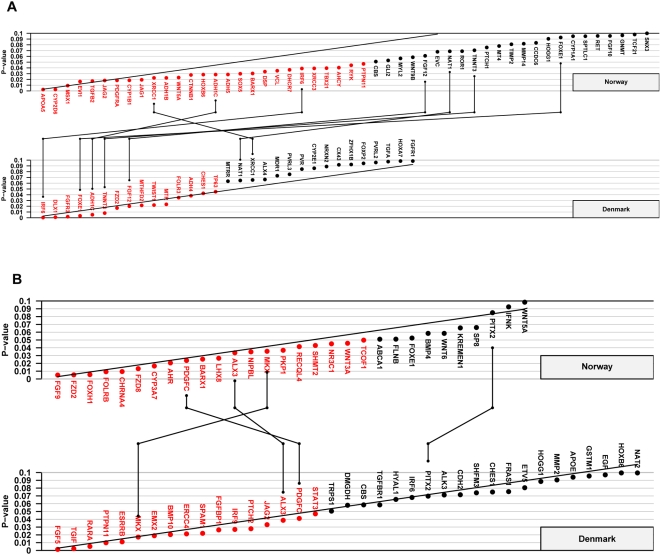
TRIMM analyses of the Norwegian and Danish samples. Schweder-Spjøtvoll plot of p-values for (A) isolated cleft lip with or without cleft palate (I-CL/P) and (B) isolated cleft palate (I-CP). All genes with p-values ≤0.1 are shown on the X-axis and ordered according to observed p-values (Y-axis). Genes with p-values ≤0.05 are highlighted in red. The sloping line represents the expected uniform distribution under the null (of no effect). Genes with p-values ≤0.1 in both the Norwegian and Danish samples are indicated by vertical lines connecting the upper (Norway) and lower (Denmark) plots.

**Figure 2:**
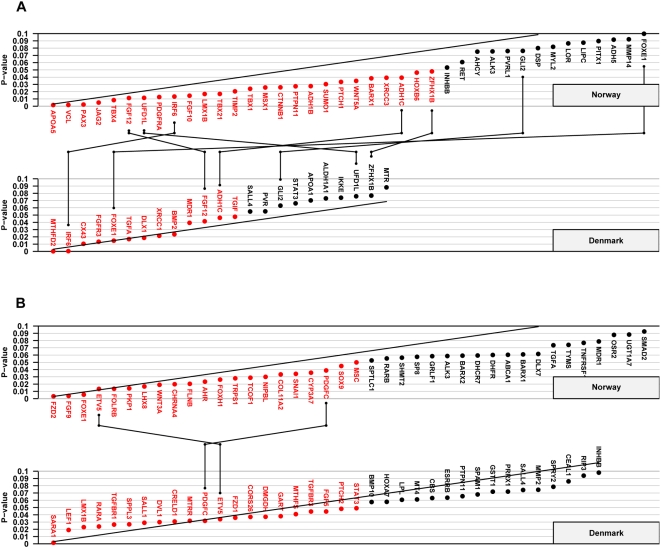
HAPLIN analyses of the Norwegian and Danish samples. Schweder-Spjøtvoll plot of p-values for (A) isolated cleft lip with or without cleft palate (I-CL/P) and (B) isolated cleft palate (I-CP). All genes with p-values ≤0.1 are shown on the X-axis and ordered according to observed p-values (Y-axis). Genes with p-values ≤0.05 are highlighted in red. The sloping line represents the expected uniform distribution under the null (of no effect). Genes that had p-values ≤0.1 in both the Norwegian and Danish samples are indicated by vertical lines connecting the upper (Norway) and lower (Denmark) plots.

**Figure 3:**
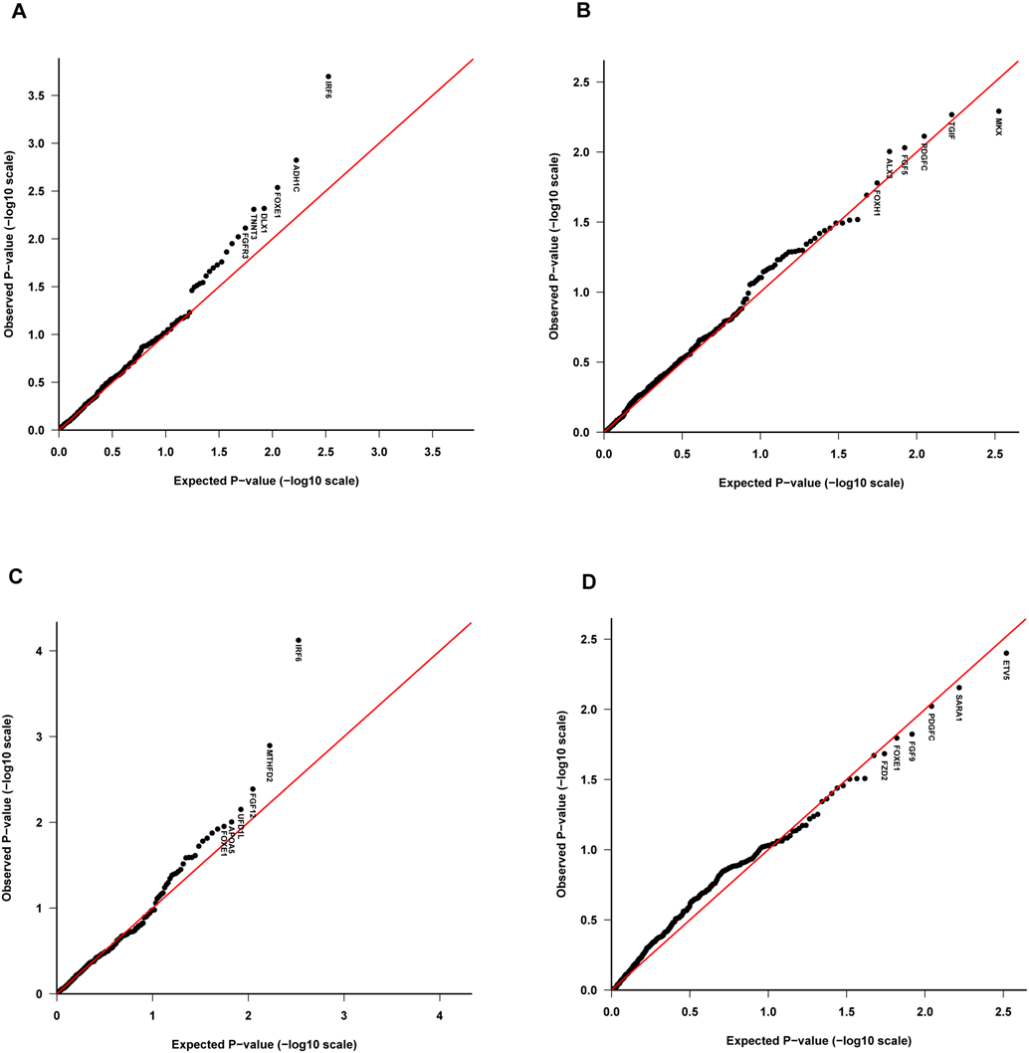
Quantile-quantile (QQ) plots of p-values. The QQ plot compares the distribution of the observed Fisher-combined p-values (−log scale) for both populations with an expected uniform distribution under the null (sloping line). The plots for I-CL/P and I-CP are provided separately for TRIMM in (A) and (B), respectively, and the corresponding plots for HAPLIN are shown in (C) and (D). Gene labels for the top six most significant genes are displayed in each plot.

**Figure 4:**
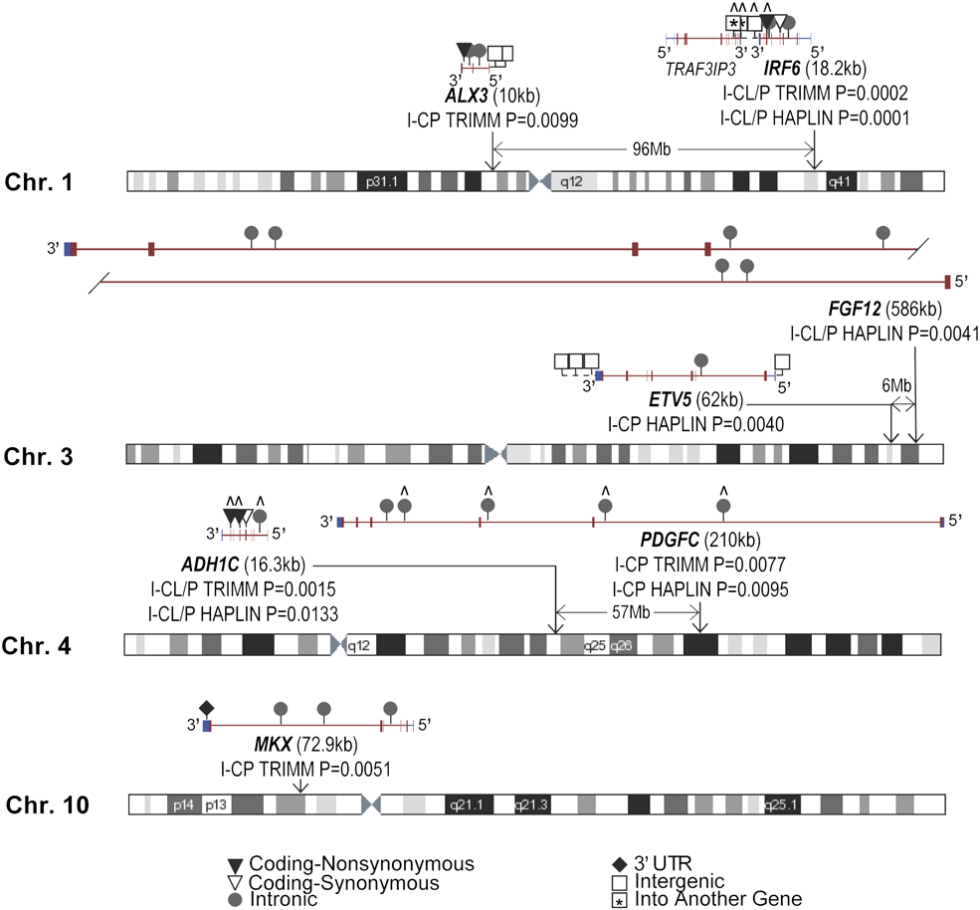
Genomic location of the seven genes identified in both populations by TRIMM and HAPLIN. Fisher-combined p-values are shown for all genes with p-values ≤0.05 in both the Norwegian and Danish populations for either analysis. The distance between genes on the same chromosome is indicated in megabases (Mb). Gene structure is shown in red for coding regions and blue for untranslated regions, with exon boxes connected by intron lines. The seven identified genes are on the minus strand compared to the reference sequence and are scaled relative to one another based on the length shown in kilobases (kb). Each SNP is represented by a symbol for its functional status as indicated at the bottom of the figure. Two SNPs represented by a square with a star were considered in the region of *IRF6*, but actually fell within the neighboring gene *TRAF3IP3*.

Finally, **Figure 4** provides a graphical summary of the main results, diagrams the genomic location of the seven genes showing associations, and details the type and position of the SNPs within each gene.

## Discussion

Of the 334 autosomal cleft candidate genes analyzed in this study, associations with seven genes—*IRF6*, *PDGFC*, *ADH1C*, *MKX*, *ALX3*, *FGF12* and *ETV5*—were replicated in the population-based samples from Norway and Denmark. As expected with this large number of statistical tests, there were many more associations with p-values <0.05 in each population. Of 334 candidate genes, 17 false positives (p<0.05) in each cleft category in each country would be expected by chance alone, and fewer than one gene would show replication across the two countries. As shown in **Figures 1** and **2**, significant associations were found with 27 genes for I-CL/P and 21 genes for I-CP in the Norwegian and Danish sample respectively using TRIMM and/or HAPLIN. Since all candidate genes in this study were chosen *a priori* for their potential roles in facial clefting, it is important to separate true associations from spurious ones. We will therefore focus this discussion on genetic associations that were replicated in both populations and that produced the smallest p-values.

### TRIMM and HAPLIN analyses

#### *I-CL/P—IRF6*, *ADH1C* and *FGF12*

We initially hypothesized similar genetic contributions to clefting in both populations because of the shared ancestry of the Norwegian and Danish populations. The genes for interferon regulatory factor 6 (*IRF6*), alcohol dehydrogenase 1C (*ADH1C*), and fibroblast growth factor 12 (*FGF12*) were strongly associated with I-CL/P in both populations using TRIMM and/or HAPLIN. An even larger number of genes were significantly associated in one population but not the other. Many are no doubt merely chance associations, although a few may reflect true risk genes with associations too weak to emerge in both Danish and Norwegian samples.

The role of *IRF6* in facial clefting is now well established [37], [38], [39], [40], [41], [42], [43], [44], and our findings provide proof-of-principle that this study design can reliably detect gene/SNP combinations where the relative risks are relatively low. *ADH1C* is the third gene of the class I alcohol dehydrogenase family consisting of *ADH1A*, *ADH1B*, and *ADH1C*. These genes are tandemly organized as a gene-cluster on chromosome 4q21–q23 and encode the alpha, beta and gamma subunits responsible for most of the ethanol-oxidizing capacity in the liver [45]. The gamma 2 enzyme was previously reported to oxidize ethanol to acetaldehyde at a lower rate than the major gamma 1 variant [46]. Furthermore, the gamma 2 allele also appears to have a protective effect on the risk of facial clefts [47].

Alcohol consumption during the first trimester of pregnancy is a recognized risk factor for facial clefts [4]. Our recent analysis of maternal binge drinking in the same Norwegian sample suggested that women who reported binge drinking were more likely to have an infant with either CL/P or CP [48]. However, our preliminary analyses show no evidence of interaction between variants in *ADH1C* and maternal alcohol consumption (results not shown). Previous support for a role of *ADH1C* in facial clefting stems from an exploratory study from the US where 64 candidate genes were investigated in a combined sample of 58 I-CL/P and I-CP case-parent triads [49]. Despite the small number of triads analyzed in that study, the strong association with *ADH1C* persisted even after correcting for multiple comparisons.

*FGF12* was significantly associated with I-CL/P in both samples in our analyses. Although numerous studies have investigated several members of the FGF and FGFR family in clefting, there are no previously published association studies with *FGF12* specifically. The FGF signaling pathway is known to play a crucial role in craniofacial development [50], and when dysregulated causes craniosynostosis and other facio-skeletal malformations in humans [51]. Using a combination of direct sequencing, association studies and *in silico* protein modeling, our group recently showed that the FGF signaling pathway may contribute to as much as 3–5% of I-CL/P [11].

Our data also suggest population-specific differences in susceptibility to facial clefts. For example, both HAPLIN and TRIMM identified *FOXE1* as being significantly associated with I-CL/P in the Danish sample, but a significant association did not emerge in the larger Norwegian sample. Conversely, both methods identified *MSX1* among the genes most significantly associated with I-CL/P in the Norwegian sample, whereas no such association was seen in the Danish sample (**Figures 1** and **2**). Although these findings do not contradict our previously reported associations with these genes [9], [52], [53], this population-specific susceptibility nevertheless highlights the problem with lack of replication across diverse populations [54], [55].

Among other genes significantly associated with facial clefts in one population but not the other, zinc finger homeobox 1b (*ZFHX1B* [MIM 605802]; *a.k.a. ZEB2*) is of particular interest. Mutations in this gene underlie the autosomal dominant Mowat-Wilson syndrome (MWS [MIM 235730]), characterized by severe mental retardation and multiple congenital anomalies [56]. Among the wide range of clinical features associated with MWS, a small subset of patients also present with facial clefts [57]. In the mouse model, cranial neural crest cells fail to dissociate and migrate when *Zfhx1b* is knocked out [58]. These cells are early progenitors of craniofacial cartilage, bone, and facial connective tissue. It is thus plausible that a failure of the cells to delaminate could underlie the distinctive facies and occasional clefting seen in MWS patients.

The paralog *Zfhx1a* (*a.k.a. Zeb1*) when knocked out in mice causes a cleft palate phenotype with 100% penetrance [59], [60]. The defect appears to be largely due to a delay in palatal elevation. In addition, the *Zfhx1a* null embryos also show reduced expression of *Jag2*, *Tgfb3* and *Mmp13*—genes known to be involved in palatogenesis. There also appears to be complex synergistic interactions between *Zfhx1a* and *Zfhx1b* during mouse embryogenesis [61]. Notably, the double *Zfhx1a/Zfhx1b* mutants have midline defects at the fusion sites of the nasal and maxillary processes.

#### *I-CP—ALX3*, *MKX*, *PDGFC* and *ETV5*

TRIMM and HAPLIN identified four genes strongly associated with I-CP in both populations. Aristaless (*ALX*)-related genes are a subset of the Paired-related homeobox genes involved in the development of craniofacial structures [62], [63]. *ALX3* encodes a nuclear protein containing a homeobox DNA-binding domain that functions as a transcriptional regulator in embryonic development. In mice, expression of *Alx3* is highly localized and the characteristic expression pattern suggests an important role in patterning of neural-crest derived mesenchyme and shaping of craniofacial structures [64]. Compound mutants of *Alx3* and *Alx4* exhibit severe craniofacial abnormalities that are otherwise absent in *Alx4* single mutants [65]. In addition, *Alx3/Alx4* double mutant newborn mice have cleft nasal regions, most likely as a result of a failure of the medial nasal processes to fuse in the facial midline [65].

The mohawk homeobox (*MKX*) gene product is another important regulator of vertebrate development [66]. In mice, *Mkx* is expressed in somite-derived cell lineages that give rise to skeletal muscle, among other cell types [67]. *Mkx* was formerly described as *Irxl1* (iroquois homeobox protein-like 1) in Liu et al. [68]. Localized in the middle of the 4.3 Mb critical region in the spontaneous *Twirler* mouse mutant, *Mkx* is a likely candidate for the *Twirler* (*Tw*) gene. Mice heterozygous for the *Twirler* mutation have inner ear defects, whereas those homozygous have cleft lip and cleft palate. *Mkx* is also highly expressed in the palatal mesenchyme during palatal growth and fusion [68]. Interestingly, both *MKX* and *ZFHX1A* (a paralog of *ZFHX1B* described above) are located within the 10p15-11 region which is associated with cleft lip and cleft palate when duplicated [69], but not when deleted [70]. Finally, Blanton and co-workers have reported strong associations between markers from the 10p13 region and I-CL/P [71].

Compared with *ALX* and *MKX*, the gene for platelet-derived growth factor C (*PDGFC*) has a well-substantiated role in palatogenesis. Linkage, association and cytogenetic studies have supported the existence of a CL/P locus in the chromosome 4q31-ter region containing the *PDGFC* locus [70], [72], [73], [74]. More recently, Choi and colleagues showed that a SNP in the *PDGFC* regulatory region decreased promoter transcriptional activity and was associated with CL/P in multiple populations [75]. Animal studies have also shown a specific role for *PDGFC* in palatal development. For example, *Pdgfc^−/−^* mice die in the perinatal period, presumably due to feeding and respiratory difficulties from having a complete cleft of the secondary palate [6]. In these mice, the palatal bones fail to extend across the roof of the oral-nasal cavity, suggesting that hypoplasia of palate tissues combined with fusion defects of the medial edge epithelia may contribute to the cleft palate seen in the *Pdgfc^−/−^* mice [6]. *In vitro* PDGFC, a potent stimulator of mitosis, is downregulated by retinoic acid in mouse embryonic palatal mesenchymal cells [76]. Reduced PDGFC by retinoic acid may cause inhibition of proliferation in palatal shelves, resulting in the pathogenesis of cleft palate in *Pdgfc^−/−^* mice or retinoic acid-treated mice. Consistent with the absence of cleft lip in *Pdgfc^−/−^* mice, the association with *PDGFC* was found exclusively in the I-CP group in our data.

Lastly, the gene encoding transcription factor ETV5 (ets variant 5) is located on chromosome 3q28 (as *FGF12* above). In the E13.5 mouse palatal epithelium, expression of *Etv5* is strong on the oral surface of the palatal shelf and overlaps to some extent with that of the FGF signaling antagonist *Spry2* [77]. Mice carrying a deletion that disrupts *Spry2* have cleft palate, and point mutations in *SPRY2* may be rare causes of I-CL/P [9]. The link between *ETV5*, *SPRY2* and members of the FGF family offers an exciting opportunity to further explore these interacting developmental pathways in the pathogenesis of cleft palate.

### Methodological aspects

#### Multiple testing

Both TRIMM and HAPLIN correct for within-gene multiple testing, ensuring the validity of the individual p-values for each gene. When assessing the overall results, no single gene remained significant after a full Bonferroni correction. However, it is generally agreed that the Bonferroni requirement of ensuring an *overall* type 1 error rate of below 5% is too strict and may result in too many false negatives [78], particularly in this study where the selected genes already had an *a priori* connection to clefting. As an alternative, we focused here on genes showing significance in both populations. As proof-of-principle, our approach consistently replicated the previously known strong associations with *IRF6* and *PDGFC*, while proposing several new candidate genes including *ETV5*, *FGF12*, *MKX*, *ADH1C* and *ALX3* for further analyses.

#### Quantile-quantile (QQ) plot

A QQ plot compares the observed p-values with the expected uniform distribution under the null when multiple tests are performed. If the plot reveals a marked difference between the expected and observed p-value distribution, this may indicate that a number of genes are significantly associated with disease, although individual genes may not have a marginal effect that is strong enough to withstand Bonferroni correction. We used the plot to test for within-gene differences in SNP genotype distribution across the two national samples, as well as to evaluate the results of the genetic association tests by TRIMM and HAPLIN.

Our data revealed systematic differences in genotypes for a subset of the SNPs across the Norwegian and Danish samples. These differences cannot be simply ascribed to random genotyping errors, as these had already been filtered out by our initial HWE check and data-cleaning steps. Biases in genotype call rates may arise if DNA comes from different sources (e.g. blood versus buccal swab). However, the genotype call rates were 99.6% for DNA extracted from blood (Norwegian sample) and 99.1% for DNA extracted from buccal swabs (Danish sample). Even if the marked heterogeneity is a true indication of population differences, different genotype frequencies do not imply differences in gene-effects on phenotype. Moreover, our methods, based on analysis of case-parent triads, insulate against population-based differences of this kind.

QQ plots of the combined p-values from the TRIMM and HAPLIN analyses for I-CL/P and I-CP are depicted in **Figure 3**. Of the two cleft categories, the QQ plot for I-CP showed the least evidence of association with the selected SNPs, yet this is the cleft subtype with the stronger recurrence risk in families [79]. If there are real but weak genetic effects, these would still be expected to deviate from the uniform distribution. The lack of associated genes in I-CP may be a direct consequence of insufficient SNP coverage rather than a lack of statistical power. Also, there may be important gene-gene/gene-environment interactions that were not considered in the current analyses.

#### SNP resolution

Our selection of htSNPs was based primarily on genotype information publicly available from the International HapMap Consortium. Although the use of haplotypes may guard against the loss of power in detecting an association signal, inadequate SNP resolution might still be a concern in this study, as pointed out for the lack of associated genes in I-CP. Adequate representation of the genetic architecture of each candidate gene is feasible only if htSNPs are selected from complete re-sequencing of the genes in the two populations examined. Furthermore, the effect of SNP resolution on inferred haplotype block structure is not fully understood. Studies using lower marker density identify progressively longer blocks, and vice-versa [80]. The small set of reference samples (30 CEPH trios) of [Western] European descent in HapMap may not be sufficiently representative of the Danish or Norwegian population, especially if common alleles are overrepresented and rare alleles more strongly linked to facial clefting are underrepresented. Hence, blocks detected at low marker density may not adequately reflect the true genetic diversity in our data.

#### HAPLIN and TRIMM

These two analytic methods are both designed to detect multi-marker transmission distortion, but they accomplish this in different ways. TRIMM is non-parametric and can accommodate population structure, deviation from HWE, multiple SNPs, missing SNPs and non-negligible recombination rates. TRIMM does not attempt to infer haplotypes and, consequently, is computationally efficient. TRIMM performs best when there is one important risk haplotype or one SNP associated with risk. When applied to a set of SNPs within a gene, TRIMM accounts for within-gene SNP correlations by permuting alleles at all SNPs simultaneously. HAPLIN on the other hand is better suited to handle more complicated scenarios, for example, when there is more than one risk-associated haplotype. Being parametric, HAPLIN estimates the full haplotype distribution over a set of SNPs and also estimates relative risks associated with each haplotype. Through the use of a full maximum likelihood model, it produces a complete description of the “risk structure” over the set of haplotypes in a region. HAPLIN requires HWE, assumes no recombination, and is computationally more demanding.

Of particular relevance to our analyses here is that if one of the genotyped SNPs has a direct effect on disease risk, independently of the other SNPs, TRIMM would be most likely to detect it. Alternatively, if the specific risk locus has not been genotyped directly, it is still likely to be associated with a haplotype composed of SNPs measured in the surrounding region. Since HAPLIN scans window-lengths of 3 and 4 SNPs, it will typically surround such a locus with 1–2 SNPs on either side, provided the disease locus resides within the genotyped region. In such cases, the sliding-window approach of HAPLIN should have a better chance of detecting the true association.

#### Replication of findings

Replication has a central role in the evaluation of association findings, particularly in studies involving large numbers of statistical tests [55]. To help separate true associations from spurious ones, we looked for associations that were consistent across the two populations of shared ancestry, which are more likely to represent true associations as opposed to spurious associations that show up randomly—even in populations of similar ancestral background. We opted not to combine the Norwegian and Danish samples (although this would have boosted power) because we had no prior data to assume that the same haplotype(s) contribute to the risk of clefts in both populations. Although our data do not speak to that issue, our test for heterogeneity between these two populations showed marked and systematic differences in genotypes for a subset of the SNPs (data not shown), suggesting ancestral differences between the two populations.

### Concluding remarks

In this search among 357 candidate genes for facial clefting, we found a relatively small number of genes to be significantly associated with either I-CL/P or I-CP. The modest number of genes associated with this strongly genetic birth defect has several possible interpretations. It may suggest the presence of etiological variants not genotyped in the current study (copy number variants for example), or cleft candidate genes overlooked in our gene selection. It may also reflect the effects of non-additive interactions with other genetic variants and/or with environmental factors that have been and will be explored in other studies [81]. Furthermore, it is possible that the remaining genetic risks arise in the context of allelic heterogeneity, in which case they would not be detectable by the current LD-based association approach. We have analyzed only individual genes; epistasis between these may play an important role as well. There is also the possibility that, regardless of apparent similarities between the Norwegian and Danish populations, these two populations may have inherently distinct genetic susceptibilities to facial clefting.

The use of two powerful and complementary statistical methods for haplotype analysis of genetic data from offspring-parent triads, two well-defined cleft phenotypes, two national cleft cohorts of similar ancestry, and the use of a very large number of SNPs in many genes (10 times the number of SNPs previously used to look for cleft associations), provided a powerful opportunity to look for associations that are most likely to be genuine. Our approach effectively replicated the strongest previously known association with cleft lip—the one with *IRF6*—in both populations and by both methods, and suggested new associations with *ADH1C* and *PDGFC*. Finally, the novel genes detected here (*MKX*, *ALX3*, *ETV5*, and *FGF12*) provide new insights for future analyses of this common and complex birth defect.

## Supporting Information

Table S1 Overview of the 357 candidate genes selected for facial clefts.
(0.77 MB DOC)

Table S2 Genes and SNPs.
(1.78 MB DOC)

Table S3 TRIMM results for I-CL/P.
(0.09 MB DOC)

Table S4 TRIMM results for I-CP.
(0.09 MB DOC)

Table S5 HAPLIN results for I-CL/P.
(0.08 MB DOC)

Table S6 HAPLIN results for I-CP.
(0.08 MB DOC)
