# Supplementary material for: Genetic Determinants of Facial Clefting: Analysis of 357 Candidate Genes Using Two National Cleft Studies from Scandinavia
Source: PLoS One. 2009 Apr 29;4(4):e5385. doi: 10.1371/journal.pone.0005385 (PMC2671138; doi:10.1371/journal.pone.0005385)
Supplement: Table S1 — Overview of the 357 candidate genes selected for facial clefts. (0.77 MB DOC) [file pone.0005385.s001.doc]

**Table S1.** Overview of the 357 candidate genes selected for facial clefts.

| **Gene Name** ^a^ | **Gene ID** ^b^ | **Chromosome** | **Gene Description** | **Molecular Function | Biological Process | Pathway** ^c^ |
| --- | --- | --- | --- | --- |
| ***ABCA1*** | 19 | 9q31.1 | ATP-binding cassette, sub-family A (ABC1), member 1. The membrane-associated protein encoded by this gene is a member of the superfamily of ATP-binding cassette (ABC) transporters. ABC proteins transport various molecules across extra- and intracellular membranes. ABC genes are divided into seven distinct subfamilies (ABC1, MDR/TAP, MRP, ALD, OABP, GCN20, and White). This protein is a member of the ABC1 subfamily. Members of the ABC1 subfamily comprise the only major ABC subfamily found exclusively in multicellular eukaryotes. With cholesterol as its substrate, this protein functions as a cholesterol efflux pump in the cellular lipid removal pathway. Mutations in this gene have been associated with Tangier's disease and familial high-density lipoprotein deficiency. | ATP-binding cassette (ABC) transporter | Lipid and fatty acid transport; Phospholipid metabolism; Cholesterol metabolism; Transport | N/A |
| ***ACTN1*** | 87 | 14q24.1-q24.2 | Actinin, alpha 1. Alpha actinins belong to the spectrin gene superfamily which represents a diverse group of cytoskeletal proteins, including the alpha and beta spectrins and dystrophins. Alpha actinin is an actin-binding protein with multiple roles in different cell types. In nonmuscle cells, the cytoskeletal isoform is found along microfilament bundles and adherens-type junctions, where it is involved in binding actin to the membrane. In contrast, skeletal, cardiac, and smooth muscle isoforms are localized to the Z-disc and analogous dense bodies, where they help anchor the myofibrillar actin filaments. This gene encodes a nonmuscle, cytoskeletal, alpha actinin isoform and maps to the same site as the structurally similar erythroid beta spectrin gene. | Non-motor actin binding protein | Cell structure | Integrin signaling pathway->alpha actinin |
| ***ADAM17*** | 6868 | [2p25](http://www.ncbi.nlm.nih.gov/mapview/maps.cgi?ORG=hum&chr=2&maps=cntg-r,clone,model,ugHs,loc&VERBOSE=ON&cmd=focus&fill=40&size=40&query=ADAM17%5BSYM%5D) | ADAM metallopeptidase domain 17. This gene encodes a member of the ADAM (a disintegrin and metalloprotease domain) family. Members of this family are membrane-anchored proteins structurally related to snake venom disintegrins, and have been implicated in a variety of biologic processes involving cell-cell and cell-matrix interactions, including fertilization, muscle development, and neurogenesis. The protein encoded by this gene functions as a tumor necrosis factor-alpha converting enzyme; binds mitotic arrest deficient 2 protein; and also plays a prominent role in the activation of the Notch signaling pathway. | Metalloprotease | Proteolysis; Cell adhesion-mediated signaling; Heart development | Notch signaling pathway->TACE |
| ***ADH1A*** | 124 | 4q21-q23 | Alcohol dehydrogenase 1A (class I), alpha polypeptide. This gene encodes class I alcohol dehydrogenase, alpha subunit, which is a member of the alcohol dehydrogenase family. Members of this enzyme family metabolize a wide variety of substrates, including ethanol, retinol, other aliphatic alcohols, hydroxysteroids, and lipid peroxidation products. Class I alcohol dehydrogenase, consisting of several homo- and heterodimers of alpha, beta, and gamma subunits, exhibits high activity for ethanol oxidation and plays a major role in ethanol catabolism. Three genes encoding alpha, beta and gamma subunits are tandemly organized in a genomic segment as a gene cluster. This gene is monomorphic and predominant in fetal and infant livers, whereas the genes encoding beta and gamma subunits are polymorphic and strongly expressed in adult livers. | Dehydrogenase; Reductase | Other carbohydrate metabolism; Other carbon metabolism | N/A |
| ***ADH1B*** | 125 | 4q21-q23 | Alcohol dehydrogenase IB (class I), beta polypeptide. The protein encoded by this gene is a member of the alcohol dehydrogenase family. Members of this enzyme family metabolize a wide variety of substrates, including ethanol, retinol, other aliphatic alcohols, hydroxysteroids, and lipid peroxidation products. This encoded protein, consisting of several homo- and heterodimers of alpha, beta, and gamma subunits, exhibits high activity for ethanol oxidation and plays a major role in ethanol catabolism. Three genes encoding alpha, beta and gamma subunits are tandemly organized in a genomic segment as a gene cluster. | Dehydrogenase; Reductase | Other carbohydrate metabolism; Other carbon metabolism | N/A |
| ***ADH1C*** | 126 | 4q21-q23 | Alcohol dehydrogenase 1C (class I), gamma polypeptide. This gene encodes class I alcohol dehydrogenase, gamma subunit, which is a member of the alcohol dehydrogenase family. Members of this enzyme family metabolize a wide variety of substrates, including ethanol, retinol, other aliphatic alcohols, hydroxysteroids, and lipid peroxidation products. Class I alcohol dehydrogenase, consisting of several homo- and heterodimers of alpha, beta, and gamma subunits, exhibits high activity for ethanol oxidation and plays a major role in ethanol catabolism. Three genes encoding alpha, beta and gamma subunits are tandemly organized in a genomic segment as a gene cluster. | Dehydrogenase; Reductase | Other carbohydrate metabolism; Other carbon metabolism | N/A |
| ***ADH4*** | 127 | 4q21-q24 | Alcohol dehydrogenase 4 (class II), pi polypeptide. This gene encodes class II alcohol dehydrogenase 4 pi subunit, which is a member of the alcohol dehydrogenase family. Members of this enzyme family metabolize a wide variety of substrates, including ethanol, retinol, other aliphatic alcohols, hydroxysteroids, and lipid peroxidation products. Class II alcohol dehydrogenase is a homodimer composed of 2 pi subunits. It exhibits a high activity for oxidation of long-chain aliphatic alcohols and aromatic alcohols and is less sensitive to pyrazole. This gene is localized to chromosome 4 in the cluster of alcohol dehydrogenase genes. | Dehydrogenase; Reductase | Other carbohydrate metabolism; Other carbon metabolism | N/A |
| ***ADH5*** | 128 | 4q21-q25 | Alcohol dehydrogenase 5 (class III), chi polypeptide. This gene encodes glutathione-dependent formaldehyde dehydrogenase or class III alcohol dehydrogenase chi subunit, which is a member of the alcohol dehydrogenase family. Members of this family metabolize a wide variety of substrates, including ethanol, retinol, other aliphatic alcohols, hydroxysteroids, and lipid peroxidation products. Class III alcohol dehydrogenase is a homodimer composed of 2 chi subunits. It has virtually no activity for ethanol oxidation, but exhibits high activity for oxidation of long-chain primary alcohols and for oxidation of S-hydroxymethyl-glutathione, a spontaneous adduct between formaldehyde and glutathione. This enzyme is an important component of cellular metabolism for the elimination of formaldehyde, a potent irritant and sensitizing agent that causes lacrymation, rhinitis, pharyngitis, and contact dermatitis. | Dehydrogenase; Reductase | Other carbohydrate metabolism; Other carbon metabolism | N/A |
| ***AHCY*** | 191 | 20cen-q13.1 | S-adenosylhomocysteine hydrolase. This gene catalyzes the reversible hydrolysis of S-adenosylhomocysteine (AdoHcy) to adenosine (Ado) and L-homocysteine (Hcy). Thus, it regulates the intracellular S-adenosylhomocysteine (SAH) concentration thought to be important for transmethylation reactions. Deficiency in this protein is one of the different causes of hypermethioninemia. S-adenosylhomocysteine hydrolase belongs to the adenosylhomocysteinase family. | Other hydrolase | Purine metabolism | N/A |
| ***AHR*** | 196 | 7p15 | Aryl hydrocarbon receptor. This gene encodes a ligand-activated transcription factor involved in the regulation of biological responses to planar aromatic hydrocarbons. This receptor has been shown to regulate xenobiotic-metabolizing enzymes such as cytochrome P450. Its ligands included a variety of aromatic hydrocarbons. | Other receptor; Basic helix-loop-helix transcription factor | Ligand-mediated signaling; Detoxification | N/A |
| ***AIP*** | 9049 | 11q13 | Aryl hydrocarbon receptor interacting protein. AIP may play a positive role in AHR-mediated signalling possibly by influencing its receptivity for ligand and/or its nuclear targeting. AIP is the cellular negative regulator of the HBV X protein. | Chaperone | Vision | N/A |
| ***ALDH1A1*** | 216 | 9q21.13 | Aldehyde dehydrogenase 1 family, member A1. This protein belongs to the aldehyde dehydrogenases family of proteins. Aldehyde dehydrogenase is the second enzyme of the major oxidative pathway of alcohol metabolism. Two major liver isoforms of this enzyme, cytosolic and mitochondrial, can be distinguished by their electrophoretic mobilities, kinetic properties, and subcellular localizations. Most Caucasians have two major isozymes, while approximately 50% of Orientals have only the cytosolic isozyme, missing the mitochondrial isozyme. A remarkably higher frequency of acute alcohol intoxication among Orientals than among Caucasians could be related to the absence of the mitochondrial isozyme. This gene encodes a cytosolic isoform, which has a high affinity for aldehydes. | Dehydrogenase | Other carbon metabolism | 5-Hydroxytryptamine degradation->Aldehyde Dehydrogenase |
| ***ALK3*** | 657 | 10q22.3 | Same as BMPR1A or bone morphogenetic protein receptor, type IA. The bone morphogenetic protein (BMP) receptors are a family of transmembrane serine/threonine kinases that include the type I receptors BMPR1A and BMPR1B and the type II receptor BMPR2. These receptors are also closely related to the activin receptors, ACVR1 and ACVR2. The ligands of these receptors are members of the TGF-beta superfamily. TGF-betas and activins transduce their signals through the formation of heteromeric complexes with 2 different types of serine (threonine) kinase receptors: type I receptors of about 50-55 kD and type II receptors of about 70-80 kD. Type II receptors bind ligands in the absence of type I receptors, but they require their respective type I receptors for signaling, whereas type I receptors require their respective type II receptors for ligand binding. | Molecular function unclassified | Biological process unclassified | N/A |
| ***ALK6*** | 658 | 4q22-q24 | Same as BMPR1B or bone morphogenetic protein receptor, type IB. This gene encodes a member of the bone morphogenetic protein (BMP) receptor family of transmembrane serine/threonine kinases. The ligands of this receptor are BMPs, which are members of the TGF-beta superfamily. BMPs are involved in endochondral bone formation and embryogenesis. These proteins transduce their signals through the formation of heteromeric complexes of 2 different types of serine (threonine) kinase receptors: type I receptors of about 50-55 kD and type II receptors of about 70-80 kD. Type II receptors bind ligands in the absence of type I receptors, but they require their respective type I receptors for signaling, whereas type I receptors require their respective type II receptors for ligand binding. Mutations in this gene have been associated with primary pulmonary hypertension. This gene expresses two transcript variants. | Molecular function unclassified | Biological process unclassified | N/A |
| ***ALX3*** | 257 | [1p21-p13](http://www.ncbi.nlm.nih.gov/mapview/maps.cgi?ORG=hum&chr=1&maps=cntg-r,clone,model,ugHs,loc&VERBOSE=ON&cmd=focus&fill=40&size=40&query=ALX3%5BSYM%5D) | Aristaless-like homeobox 3. This gene encodes a nuclear protein with a homeobox DNA-binding domain that functions as a transcriptional regulator involved in cell-type differentiation and development. Preferential methylation of this gene's promoter is associated with advanced-stage neuroblastoma tumors. | Homeobox transcription factor; Other DNA-binding protein | mRNA transcription regulation; Segment specification; Neurogenesis | N/A |
| ***ALX4*** | 60529 | [11p11.2](http://www.ncbi.nlm.nih.gov/mapview/maps.cgi?ORG=hum&chr=11&maps=cntg-r,clone,model,ugHs,loc&VERBOSE=ON&cmd=focus&fill=40&size=40&query=ALX4%5BSYM%5D) | Aristaless-like homeobox 4. The ALX4 mutation p.R218Q tends to result in persistent cranium bifidum and is associated with anatomical abnormalities of the posterior fossa. One subject with parietal foramina whose deletion does not include ALX4 indicates that ALX4 in this subject may be rendered functionally haploinsufficient by a position effect. | Homeobox transcription factor; Other DNA-binding protein | mRNA transcription regulation; Segment specification; Neurogenesis | N/A |
| ***AMT*** | 275 | 3p21.2-p21.1 | Aminomethyltransferase. The enzyme system for cleavage of glycine (glycine cleavage system; EC 2.1.2.10), which is confined to the mitochondria, is composed of 4 protein components: P protein (a pyridoxal phosphate-dependent glycine decarboxylase; MIM 238300), H protein (a lipoic acid-containing protein; MIM 238330), T protein (a tetrahydrofolate-requiring enzyme), and L protein (a lipoamide dehydrogenase; MIM 238331). Glycine encephalopathy (GCE; MIM 605899) may be due to a defect in any one of these enzymes. | Dehydrogenase | Amino acid catabolism; Other metabolism | N/A |
| ***APE1*** | 328 | 14q11.2-q12 | Same as APEX1 or APEX nuclease (multifunctional DNA repair enzyme) 1. Apurinic/apyrimidinic (AP) sites occur frequently in DNA molecules by spontaneous hydrolysis, by DNA damaging agents or by DNA glycosylases that remove specific abnormal bases. AP sites are pre-mutagenic lesions that can prevent normal DNA replication so the cell contains systems to identify and repair such sites. Class II AP endonucleases cleave the phosphodiester backbone 5' to the AP site. This gene encodes the major AP endonuclease in human cells. Splice variants have been found for this gene; all encode the same protein. | Exodeoxyribonuclease; Endodeoxyribonuclease | DNA repair | N/A |
| ***APOA1*** | 335 | 11q23-q24 | Apolipoprotein A-I. This gene encodes apolipoprotein A-I, which is the major protein component of high density lipoprotein (HDL) in plasma. The protein promotes cholesterol efflux from tissues to the liver for excretion, and it is a cofactor for lecithin cholesterolacyltransferase (LCAT) which is responsible for the formation of most plasma cholesteryl esters. This gene is closely linked with two other apolipoprotein genes on chromosome 11. Defects in this gene are associated with HDL deficiencies, including Tangier disease, and with systemic non-neuropathic amyloidosis. | Transporter; Apolipoprotein | Lipid and fatty acid transport; Transport | N/A |
| ***APOA1BP*** | 128240 | 1q22-q21.2 | Apolipoprotein A-I binding protein. The product of this gene interacts with apolipoprotein A-I (apoA-I), the major apolipoprotein of high-density lipoproteins (HDLs). It is secreted into some bodily fluids, and its synthesis and secretion are stimulated in vitro by incubating cells with apoA-I. The human genome contains related pseudogenes. | Molecular function unclassified | Biological process unclassified | N/A |
| ***APOA5*** | 116519 | 11q23 | Apolipoprotein A-V. The protein encoded by this gene is an apolipoprotein and an important determinant of plasma triglyceride levels, a major risk factor for coronary artery disease. It is a component of high density lipoprotein and is highly similar to a rat protein that is upregulated in response to liver injury. This gene uses alternate polyadenylation sites and is located proximal to the apolipoprotein gene cluster on chromosome 11q23. | Transporter; Apolipoprotein | Lipid and fatty acid transport; Transport; Blood circulation and gas exchange | N/A |
| ***APOB*** | 338 | 2p24-p23 | Apolipoprotein B (including Ag(x) antigen). Apolipoprotein B (ApoB) is the main apolipoprotein of chylomicrons and low density lipoproteins (LDL). The protein occurs in the plasma in 2 main isoforms, apoB-48 and apoB-100. The first is synthesized exclusively by the gut, the second by the liver. The intestinal (B-48) and hepatic (B-100) forms of apoB are coded by a single gene and by a single mRNA transcript larger than 16 kb. The 2 proteins share a common amino terminal sequence. From structural studies, it is thought that apoB-48 represents the amino-terminal 47% of apoB-100 and that the carboxyl terminus of apoB-48 is in the vicinity of residue 2151 of mature apoB-100. Apolipoprotein B-48, a shortened form of apoB-100 lacking the LDL-receptor region, is a product generated when a stop codon (UAA) at residue 2180 is created by RNA editing. | Apolipoprotein | Lipid and fatty acid transport; Transport | N/A |
| ***APOC2*** | 344 | 19q13.2 | Apolipoprotein C-II. The protein encoded by this gene is secreted in plasma where it is a component of very low density lipoprotein. This protein activates the enzyme lipoprotein lipase, which hydrolyzes triglycerides and thus provides free fatty acids for cells. Mutations in this gene cause hyperlipoproteinemia type IB, characterized by hypertriglyceridemia, xanthomas, and increased risk of pancreatitis and early atherosclerosis. | Transporter; Apolipoprotein; Lipase | Lipid and fatty acid transport; Lipid metabolism; Transport | N/A |
| ***APOE*** | 348 | 19q13.2 | Apolipoprotein E. Chylomicron remnants and very low density lipoprotein (VLDL) remnants are rapidly removed from the circulation by receptor-mediated endocytosis in the liver. Apolipoprotein E, a main apoprotein of the chylomicron, binds to a specific receptor on liver cells and peripheral cells. ApoE is essential for the normal catabolism of triglyceride-rich lipoprotein constituents. The APOE gene is mapped to chromosome 19 in a cluster with APOC1 and APOC2. Defects in apolipoprotein E result in familial dysbetalipoproteinemia, or type III hyperlipoproteinemia (HLP III), in which increased plasma cholesterol and triglycerides are the consequence of impaired clearance of chylomicron and VLDL remnants. | Transporter; Apolipoprotein | Lipid and fatty acid transport; Transport | N/A |
| ***ARNT*** | 405 | 1q21.3 | Aryl hydrocarbon receptor nuclear translocator. The aryl hydrocarbon (Ah) receptor is involved in the induction of several enzymes that participate in xenobiotic metabolism. The ligand-free, cytosolic form of the Ah receptor is complexed to heat shock protein 90. Binding of ligand, which includes dioxin and polycyclic aromatic hydrocarbons, results in translocation of the ligand-binding subunit only to the nucleus. Induction of enzymes involved in xenobiotic metabolism occurs through binding of the ligand-bound Ah receptor to xenobiotic responsive elements in the promoters of genes for these enzymes. This gene encodes a protein that forms a complex with the ligand-bound Ah receptor, and is required for receptor function. The encoded protein has also been identified as the beta subunit of a heterodimeric transcription factor, hypoxia-inducible factor 1 (HIF1). A t(1;12)(q21;p13) translocation, which results in a TEL-ARNT fusion protein, is associated with acute myeloblastic leukemia. Three alternatively spliced variants encoding different isoforms have been described for this gene. | Basic helix-loop-helix transcription factor | mRNA transcription regulation | Hypoxia response via HIF activation->hypoxia inducible factor-1-beta |
| ***ARNT2*** | 9915 | 15q24 | Aryl-hydrocarbon receptor nuclear translocator 2. This gene encodes a member of the basic-helix-loop-helix-Per-Arnt-Sim (bHLH-PAS) superfamily of transcription factors. The encoded protein acts as a partner for several sensor proteins of the bHLH-PAS family, forming heterodimers with the sensor proteins that bind regulatory DNA sequences in genes responsive to developmental and environmental stimuli. Under hypoxic conditions, the encoded protein complexes with hypoxia-inducible factor 1alpha in the nucleus and this complex binds to hypoxia-responsive elements in enhancers and promoters of oxygen-responsive genes. A highly similar protein in mouse forms functional complexes with both aryl hydrocarbon receptors and Single-minded proteins, suggesting addition roles for the encoded protein in the metabolism of xenobiotic compounds and the regulation of neurogenesis, respectively. | Basic helix-loop-helix transcription factor | mRNA transcription regulation | N/A |
| ***ARVCF*** | 421 | 22q11.21 | Armadillo Repeat gene deleted in Velo-Cardio-Facial syndrome (ARVCF). ARVCF is a member of the catenin family which play an important role in the formation of adherens junction complexes, which are thought to facilitate communication between the inside and outside environments of a cell. ARVCF gene was isolated in the search for the genetic defect responsible for the autosomal dominant Velo-Cardio-Facial syndrome (VCFS) a relatively common human disorder with phenotypic features including cleft palate, conotruncal heart defects and facial dysmorphology. ARVCF gene encodes a protein containing two motifs, a coiled coil domain in the N-terminus and a 10 armadillo repeat sequence in the midregion. Since these sequences can facilitate protein-protein interactions ARVCF is thought to function in a protein complex. In addition, ARVCF contains a predicted nuclear-targeting sequence suggesting that it may have a function as a nuclear protein. | Cell adhesion molecule; Other cell junction protein | Cell communication; Cell adhesion | N/A |
| ***ARX*** | 170302 | Xp22.1-p21.3 | Aristaless related homeobox. This gene is a homeobox-containing gene expressed during development. The expressed protein contains two conserved domains, a C-peptide (or aristaless domain) and the prd-like class homeobox domain. It is a member of the group-II aristaless-related protein family whose members are expressed primarily in the central and/or peripheral nervous system. This gene is thought to be involved in CNS development. Mutations in this gene cause X-linked mental retardation and epilepsy. | Homeobox transcription factor; Other DNA-binding protein | mRNA transcription regulation; Segment specification; Neurogenesis | N/A |
| ***ATIC*** | 471 | 2q35 | 5-aminoimidazole-4-carboxamide ribonucleotide formyltransferase/IMP cyclohydrolase. Polymorphisms of reduced folate carrier, aminoimidazole carboxamide ribonucleotide transformylase, and thymidylate synthase genes contribute to the therapeutic response in rheumatoid arthritis patients to methotrexate. | Methyltransferase; Other hydrolase | Purine metabolism | De novo purine biosynthesis->AICAR transformylase; De novo purine biosynthesis->IMP cyclohydrolase |
| ***ATR*** | 545 | 3q22-q24 | Ataxia telangiectasia and Rad3 related. The protein encoded by this gene belongs the PI3/PI4-kinase family, and is most closely related to ATM, a protein kinase encoded by the gene mutated in ataxia telangiectasia. This protein and ATM share similarity with Schizosaccharomyces pombe rad3, a cell cycle checkpoint gene required for cell cycle arrest and DNA damage repair in response to DNA damage. This kinase has been shown to phosphorylate checkpoint kinase CHK1, checkpoint proteins RAD17, and RAD9, as well as tumor suppressor protein BRCA1. Mutations of this gene are associated with Seckel syndrome. An alternatively spliced transcript variant of this gene has been reported, however, its full length nature is not known. Transcript variants utilizing alternative polyA sites exist. | Non-receptor serine/threonine protein kinase; Nucleotide kinase | DNA repair; Protein phosphorylation; Cell cycle control | p53 pathway feedback loops 2->ATM; p53 pathway->Ataxia telangiectasia mutated (ATM) and Rad3-related (ATR) |
| ***ATRX*** | 546 | Xq13.1-q21.1 | The protein encoded by this gene contains an ATPase/helicase domain, and thus it belongs to the SWI/SNF family of chromatin remodeling proteins. The mutations of this gene are associated with an X-linked mental retardation (XLMR) syndrome most often accompanied by alpha-thalassemia (ATRX) syndrome. Theses mutations have been shown to cause diverse changes in the pattern of DNA methylation, which may provide a link between chromatin remodeling, DNA methylation, and gene expression in developmental processes. This protein is found to undergo cell cycle-dependent phosphorylation, which regulates its nuclear matrix and chromatin association, and suggests its involvement in the gene regulation at interphase and chromosomal segregation in mitosis. Three alternatively spliced transcript variants encoding distinct isoforms have been reported. | DNA helicase | mRNA transcription regulation | N/A |
| ***BAMBI*** | 25805 | 10p12.3-p11.2 | BMP and activin membrane-bound inhibitor homolog (Xenopus laevis). This gene encodes a transmembrane glycoprotein related to the type I receptors of the transforming growth factor-beta (TGF-beta) family, whose members play important roles in signal transduction in many developmental and pathological processes. The encoded protein however is a pseudoreceptor, lacking an intracellular serine/threonine kinase domain required for signaling. Similar proteins in frog, mouse and zebrafish function as negative regulators of TGF-beta, which has led to the suggestion that the encoded protein may function to limit the signaling range of the TGF-beta family during early embryogenesis. | Molecular function unclassified | Biological process unclassified | TGF-beta signaling pathway->BMP and activin membrane-bound inhibitor |
| ***BARX1*** | 56033 | 9q12 | BarH-like homeobox 1. This gene belongs to the Bar subclass of the homeobox gene family. The function of this gene has not yet been determined; however, studies in the mouse and chick homolog suggest a role in developing teeth and craniofacial mesenchyme of neural crest origin. The role of these homologs implicates the human gene as a candidate for unmapped disorders involving tooth and jaw development. | Homeobox transcription factor; Other DNA-binding protein | mRNA transcription regulation; Neurogenesis; Skeletal development | N/A |
| ***BARX2*** | 8538 | 11q25 | BarH-like homeobox 2. BARX2 is a member of the homeobox gene family which are regulators of place-dependent morphogenesis and play important roles in controlling the expression patterns of cell adhesion molecules. The homeodomain encoded by Barx2 is 87% identical to that of Barx1, and both genes are related to genes at the Bar locus of Drosophila melanogaster. Barx1 and Barx2 also encode an identical stretch of 17 residues downstream of the homeobox. It is suggested that Barx2 may differentially control the expression of L1 and other target genes during embryonic development. | Homeobox transcription factor; Other DNA-binding protein | mRNA transcription regulation; Neurogenesis; Skeletal development | N/A |
| ***BCL3*** | 602 | [19q13.1-q13.2](http://www.ncbi.nlm.nih.gov/mapview/maps.cgi?ORG=hum&chr=19&maps=cntg-r,clone,model,ugHs,loc&VERBOSE=ON&cmd=focus&fill=40&size=40&query=BCL3%5BSYM%5D) | B-cell CLL/lymphoma 3. This gene is a proto-oncogene candidate. It is identified by its translocation into the immunoglobulin alpha-locus in some cases of B-cell leukemia. The protein encoded by this gene contains seven ankyrin repeats, which are most closely related to those found in I kappa B proteins. This protein functions as a transcriptional co-activator that activates through its association with NF-kappa B homodimers. The expression of this gene can be induced by NF-kappa B, which forms a part of the autoregulatory loop that controls the nuclear residence of p50 NF-kappa B. | Molecular function unclassified | mRNA transcription regulation | Inflammation mediated by chemokine and cytokine signaling pathway->Nuclear factor kappa-B |
| ***BCOR*** | 54880 | Xp11.4 | BCL6 co-repressor. The protein encoded by this gene was identified as an interacting corepressor of BCL6, a POZ/zinc finger transcription repressor that is required for germinal center formation and may influence apoptosis. This protein selectively interacts with the POZ domain of BCL6, but not with eight other POZ proteins. Specific class I and II histone deacetylases (HDACs) have been shown to interact with this protein, which suggests a possible link between the two classes of HDACs. At least two alternatively spliced transcript variants, which encode different isoforms, have been reported for this gene. | Molecular function unclassified | Biological process unclassified | N/A |
| ***BHMT*** | 635 | 5q13.1-q15 | Betaine-homocysteine methyltransferase. Betaine-homocysteine methyltransferase is a cytosolic enzyme that catalyzes the conversion of betaine and homocysteine to dimethylglycine and methionine, respectively. Defects in BHMT could lead to hyperhomocyst(e)inemia,but such a defect has not yet been observed. | Methyltransferase | Amino acid metabolism | N/A |
| ***BHMT2*** | 23743 | 5q13 | Betaine-homocysteine methyltransferase 2. Homocysteine is a sulfur-containing amino acid that plays a crucial role in methylation reactions. Transfer of the methyl group from betaine to homocysteine creates methionine, which donates the methyl group to methylate DNA, proteins, lipids, and other intracellular metabolites. The protein encoded by this gene is one of two methyl transferases that can catalyze the transfer of the methyl group from betaine to homocysteine. Anomalies in homocysteine metabolism have been implicated in disorders ranging from vascular disease to neural tube birth defects such as spina bifida. | Methyltransferase | Amino acid metabolism | N/A |
| ***BMP10*** | 27302 | 2p13.3 | Bone morphogenetic protein 10. The protein encoded by this gene is a member of the TGF-beta family of growth factors. Data suggest that the similar protein in mouse plays an important role in trabeculation of the embryonic heart. In human, this protein may signal through receptor serine/threonine kinases. | Growth factor | Other receptor mediated signaling pathway; Cell communication; Heart development | TGF-beta signaling pathway->Transforming growth factor beta |
| ***BMP2*** | 650 | 20p12.3 | Bone morphogenetic protein 2. The protein encoded by this gene belongs to the transforming growth factor-beta (TGFB) superfamily. The encoded protein acts as a disulfide-linked homodimer and induces bone and cartilage formation. | Other signaling molecule | Skeletal development | TGF-beta signaling pathway->Transforming growth factor beta |
| ***BMP4*** | 652 | 14q22.2 | Bone morphogenetic protein 4. The protein encoded by this gene is a member of the bone morphogenetic protein family which is part of the transforming growth factor-beta superfamily. The superfamily includes large families of growth and differentiation factors. Bone morphogenetic proteins were originally identified by an ability of demineralized bone extract to induce endochondral osteogenesis in vivo in an extraskeletal site. This particular family member plays an important role in the onset of endochondral bone formation in humans, and a reduction in expression has been associated with a variety of bone diseases, including the heritable disorder Fibrodysplasia Ossificans Progressiva. Alternative splicing in the 5' untranslated region of this gene has been described and three variants are described, all encoding an identical protein. | Other signaling molecule | Skeletal development | TGF-beta signaling pathway->Transforming growth factor beta |
| ***BMP6*** | 654 | 6p24-p23 | Bone morphogenetic protein 6. The bone morphogenetic proteins (BMPs) are a family of secreted signaling molecules that can induce ectopic bone growth. Many BMPs are part of the transforming growth factor-beta (TGFB) superfamily. BMPs were originally identified by an ability of demineralized bone extract to induce endochondral osteogenesis in vivo in an extraskeletal site. Based on its expression early in embryogenesis, the BMP encoded by this gene has a proposed role in early development. In addition, the fact that this BMP is closely related to BMP5 and BMP7 has lead to speculation of possible bone inductive activity. | Other signaling molecule | Skeletal development | TGF-beta signaling pathway->Transforming growth factor beta |
| ***BMPR2*** | 659 | 2q33-q34 | Bone morphogenetic protein receptor, type II (serine/threonine kinase). This gene encodes a member of the bone morphogenetic protein (BMP) receptor family of transmembrane serine/threonine kinases. The ligands of this receptor are BMPs, which are members of the TGF-beta superfamily. BMPs are involved in endochondral bone formation and embryogenesis. These proteins transduce their signals through the formation of heteromeric complexes of 2 different types of serine (threonine) kinase receptors: type I receptors of about 50-55 kD and type II receptors of about 70-80 kD. Type II receptors bind ligands in the absence of type I receptors, but they require their respective type I receptors for signaling, whereas type I receptors require their respective type II receptors for ligand binding. Mutations in this gene have been associated with primary pulmonary hypertension. | TGF-beta receptor; Serine/threonine protein kinase receptor; Protein kinase | Protein phosphorylation; Cytokine and chemokine mediated signaling pathway; Receptor protein serine/threonine kinase signaling pathway; Skeletal development | TGF-beta signaling pathway->TGFbeta receptor II; TGF-beta signaling pathway->TGFbeta receptors |
| ***MKX*** | 283078 | 10p12.1 | Mohawk homeobox (also known as C10orf48). | Homeobox transcription factor; Nucleic acid binding | mRNA transcription regulation | N/A |
| ***CASR*** | 846 | 3q21-q24 | Calcium-sensing receptor (hypocalciuric hypercalcemia 1, severe neonatal hyperparathyroidism) . The calcium-sensing receptor (CASR) functions as a sensor for parathyroid and kidney to determine the extracellular calcium concentration and thus helps to maintain a stable calcium concentration. Mutations that inactivate CASR cause familial hypocalciuric hypercalcemia, whereas mutations that activate CASR are the cause of autosomal dominant hypocalcemia. An altenatively spliced transcript variant encoding 1088 aa has been found for this gene, but its full-length nature has not been defined. | G-protein coupled receptor | G-protein mediated signaling; Skeletal development; Calcium ion homeostasis | N/A |
| ***CBS*** | 875 | 21q22.3 | Cystathionine-beta-synthase. The protein encoded by this gene is involved in the transsulfuration pathway. The first step of this pathway, from homocysteine to cystathionine, is catalyzed by this protein. CBS deficiency can cause homocystinuria which affects many organs and tissues, including the eyes and the skeletal, vascular and central nervous systems. | Synthase; Lyase | Amino acid biosynthesis | Cysteine biosynthesis->O-Acetylserine-lyase |
| ***CCDC6*** | 8030 | 10q21 | Coiled-coil domain containing 6. | Molecular function unclassified | Biological process unclassified | N/A |
| ***CCR1*** | 1230 | 3p21 | Chemokine (C-C motif) receptor 1. This gene encodes a member of the beta chemokine receptor family, which is predicted to be a seven transmembrane protein similar to G protein-coupled receptors. The ligands of this receptor include macrophage inflammatory protein 1 alpha (MIP-1 alpha), regulated on activation normal T expressed and secreted protein (RANTES), monocyte chemoattractant protein 3 (MCP-3), and myeloid progenitor inhibitory factor-1 (MPIF-1). Chemokines and their receptors mediated signal transduction are critical for the recruitment of effector immune cells to the site of inflammation. Knockout studies of the mouse homolog suggested the roles of this gene in host protection from inflammatory response, and susceptibility to virus and parasite. This gene and other chemokine receptor genes, including CCR2, CCRL2, CCR3, CCR5 and CCXCR1, are found to form a gene cluster on chromosome 3p. | G-protein coupled receptor | G-protein mediated signaling; Cytokine and chemokine mediated signaling pathway; Cytokine/chemokine mediated immunity; Granulocyte-mediated immunity; Cell motility | Inflammation mediated by chemokine and cytokine signaling pathway->Chemokine receptor |
| ***CCR6*** | 1235 | 6q27 | Chemokine (C-C motif) receptor 6. This gene encodes a member of the beta chemokine receptor family, which is predicted to be a seven transmembrane protein similar to G protein-coupled receptors. The gene is preferentially expressed by immature dendritic cells and memory T cells. The ligand of this receptor is macrophage inflammatory protein 3 alpha (MIP-3 alpha). This receptor has been shown to be important for B-lineage maturation and antigen-driven B-cell differentiation, and it may regulate the migration and recruitment of dentritic and T cells during inflammatory and immunological responses. Alternatively spliced transcript variants that encode the same protein have been described for this gene. | G-protein coupled receptor | G-protein mediated signaling; Cytokine and chemokine mediated signaling pathway; Cytokine/chemokine mediated immunity; Cell motility | Inflammation mediated by chemokine and cytokine signaling pathway->Chemokine receptor |
| ***CCT3*** | 7203 | 1q23 | Chaperonin containing TCP1, subunit 3 (gamma). This gene encodes a molecular chaperone that is member of the chaperonin containing TCP1 complex (CCT), also known as the TCP1 ring complex (TRiC). This complex consists of two identical stacked rings, each containing eight different proteins. Unfolded polypeptides enter the central cavity of the complex and are folded in an ATP-dependent manner. The complex folds various proteins, including actin and tubulin. Alternate transcriptional splice variants, encoding different isoforms, have been characterized. | Chaperonin | Protein folding; Protein complex assembly | N/A |
| ***CDH1*** | 999 | 16q22.1 | Cadherin 1, type 1, E-cadherin (epithelial). This gene is a classical cadherin from the cadherin superfamily. The encoded protein is a calcium dependent cell-cell adhesion glycoprotein comprised of five extracellular cadherin repeats, a transmembrane region and a highly conserved cytoplasmic tail. Mutations in this gene are correlated with gastric, breast, colorectal, thyroid and ovarian cancer. Loss of function is thought to contribute to progression in cancer by increasing proliferation, invasion, and/or metastasis. The ectodomain of this protein mediates bacterial adhesion to mammalian cells and the cytoplasmic domain is required for internalization. Identified transcript variants arise from mutation at consensus splice sites. | Cadherin | Cell adhesion-mediated signaling; Cell adhesion | Wnt signaling pathway->Cadherin; Cadherin signaling pathway->Cadherin |
| ***CDH2*** | 1000 | 18q11.2 | Cadherin 2, type 1, N-cadherin (neuronal). This gene is a classical cadherin from the cadherin superfamily. The encoded protein is a calcium dependent cell-cell adhesion glycoprotein comprised of five extracellular cadherin repeats, a transmembrane region and a highly conserved cytoplasmic tail. The protein functions during gastrulation and is required for establishment of left-right asymmetry. At certain central nervous system synapses, presynaptic to postsynaptic adhesion is mediated at least in part by this gene product. | Cadherin | Cell adhesion-mediated signaling; Cell adhesion | Wnt signaling pathway->Cadherin; Cadherin signaling pathway->Cadherin |
| ***CDKN1C*** | 1028 | 11p15.5 | Cyclin-dependent kinase inhibitor 1C (p57, Kip2). Cyclin-dependent kinase inhibitor 1C is a tight-binding inhibitor of several G1 cyclin/Cdk complexes and a negative regulator of cell proliferation. Mutations of CDKN1C are implicated in sporadic cancers and Beckwith-Wiedemann syndorome suggesting that it is a tumor suppressor candidate. | Kinase inhibitor | Cell cycle control; Cell proliferation and differentiation; Tumor suppressor | N/A |
| ***CDX4*** | 1046 | Xq13.2 | Caudal type homeobox transcription factor 4 | Homeobox transcription factor; Other DNA-binding protein | mRNA transcription regulation; Embryogenesis; Anterior/posterior patterning | N/A |
| ***CEAL1*** | 1049 | 19q13.31 | Carcinoembryonic antigen-like 1. This record was discontinued in Entrez Gene. New name CEACAM19. | CAM family adhesion molecule | Cell adhesion | N/A |
| ***CETP*** | 1071 | 16q21 | CETP: cholesteryl ester transfer protein, plasma. Cholestery ester transfer protein (CETP) transfers cholesteryl esters between lipoproteins. CETP may effect susceptibility to atherosclerosis. | Other transfer/carrier protein | Lipid and fatty acid transport; Cholesterol metabolism; Transport | N/A |
| ***CFC1*** | 55997 | 2q21.1 | Cripto, FRL-1, cryptic family 1. This gene encodes a member of the epidermal growth factor (EGF)- Cripto, Frl-1, and Cryptic (CFC) family. EGF-CFC family member proteins share a variant EGF-like motif, a conserved cysteine-rich domain, and a C-terminal hydrophobic region. These proteins play key roles in intercellular signaling pathways during vertebrate embryogenesis. Mutations in this gene can cause autosomal visceral heterotaxy. This protein is involved in left-right asymmetric morphogenesis during organ development. | Other select calcium binding proteins | Cell motility | N/A |
| ***CHD7*** | 55636 | 8q12.2 | Chromodomain helicase DNA binding protein 7. Sequenced selected CHD7 exons in non-syndromic clefting cases from Iowa and Philippines populations, as well as matched controls. Variants in non-syndromic cases were found, however, the numbers were not statistically different from the controls. Disruption of this gene is the cause of CHARGE syndrome in twins and independently confirms the role of CHD7 in CHARGE syndrome. | DNA helicase | mRNA transcription regulation | N/A |
| ***CHES1*** | 1112 | 14q24.3-q32.11 | Checkpoint suppressor 1. Checkpoint suppressor 1 is a member of the forkhead/winged helix transcription factor family. Checkpoints are eukaryotic DNA damage-inducible cell cycle arrests at G1 and G2. Checkpoint suppressor 1 suppresses multiple yeast checkpoint mutations including mec1, rad9, rad53 and dun1 by activating a MEC1-independent checkpoint pathway. | Other transcription factor; Nucleic acid binding | Carbohydrate metabolism; mRNA transcription regulation; Other receptor mediated signaling pathway; Cell communication; Vision; Embryogenesis; Anterior/posterior patterning; Segment specification; Neurogenesis; Mesoderm development; Cell cycle control; Cell proliferation and differentiation; Cell structure | Insulin/IGF pathway-protein kinase B signaling cascade->Forkhead transcription factor; PI3 kinase pathway->FOXO; Interleukin signaling pathway->Forkhead in Rhabdomyosarcoma-like 1; TGF-beta signaling pathway->Co-activators or corepressors |
| ***CHL1*** | 10752 | 3p26.1 | Cell adhesion molecule with homology to L1CAM (close homolog of L1). DEAD box proteins, characterized by the conserved motif Asp-Glu-Ala-Asp (DEAD), are putative RNA helicases. They are implicated in a number of cellular processes involving alteration of RNA secondary structure such as translation initiation, nuclear and mitochondrial splicing, and ribosome and spliceosome assembly. Based on their distribution patterns, some members of this family are believed to be involved in embryogenesis, spermatogenesis, and cellular growth and division. This gene encodes a DEAD box protein, which is an enzyme that possesses both ATPase and DNA helicase activities. This gene is a homolog of the yeast CHL1 gene, and may function to maintain chromosome transmission fidelity and genome stability. Alternative splicing results in multiple transcript variants encoding distinct isoforms. | CAM family adhesion molecule | Cell adhesion-mediated signaling; Cell adhesion; Neurogenesis | N/A |
| ***CHRNA4*** | 1137 | 20q13.2-q13.3 | Cholinergic receptor, nicotinic, alpha polypeptide 4. The nicotinic acetylcholine receptors (nAChRs) are members of a superfamily of ligand-gated ion channels that mediate fast signal transmission at synapses. After binding acetylcholine, these pentameric receptors respond by undergoing an extensive change in conformation that affects all subunits and leads to opening of an ion-conducting channel across the plasma membrane. The protein encoded by this gene is an integral membrane receptor subunit that can interact with either nAChR beta-2 or nAChR beta-4 to form a functional receptor. Mutations in this gene appear to account for a small proportion of the cases of nocturnal frontal lobe epilepsy. | Acetylcholine receptor; Ion channel | Cell communication; Cation transport; Nerve-nerve synaptic transmission; Sensory perception | Nicotinic acetylcholine receptor signaling pathway->nicotinic Acetylcholine Receptor alpha; Nicotinic acetylcholine receptor signaling pathway->Nicotinic Acetylcholine Receptor |
| ***CKM*** | 1158 | 19q13.2-q13.3 | Creatine kinase, muscle. The protein encoded by this gene is a cytoplasmic enzyme involved in energy homeostasis and is an important serum marker for myocardial infarction. The encoded protein reversibly catalyzes the transfer of phosphate between ATP and various phosphogens such as creatine phosphate. It acts as a homodimer in striated muscle as well as in other tissues, and as a heterodimer with a similar brain isozyme in heart. The encoded protein is a member of the ATP: guanido phosphotransferase protein family. | Other kinase | Muscle contraction | N/A |
| ***CLPTM1*** | 1209 | [19q13.2-q13.3](http://www.ncbi.nlm.nih.gov/mapview/maps.cgi?ORG=hum&chr=19&maps=cntg-r,clone,model,ugHs,loc&VERBOSE=ON&cmd=focus&fill=40&size=40&query=CLPTM1%5BSYM%5D) | Cleft lip and palate associated transmembrane protein 1. | Other lyase | Amino acid biosynthesis | N/A |
| ***COL11A1*** | 1301 | 1p21 | Collagen, type XI, alpha 1. This gene encodes one of the two alpha chains of type XI collagen, a minor fibrillar collagen. Type XI collagen is a heterotrimer but the third alpha chain is a post-translationally modified alpha 1 type II chain. Mutations in this gene are associated with type II Stickler syndrome and with Marshall syndrome. Three transcript variants encoding different isoforms have been identified for this gene. | Extracellular matrix structural protein | Mesoderm development | Integrin signalling pathway->Collagen |
| ***COL11A2*** | 1302 | 6p21.3 | Collagen, type XI, alpha 2. This gene encodes one of the two alpha chains of type XI collagen, a minor fibrillar collagen. It is located on chromosome 6 very close to but separate from the gene for retinoid X receptor beta. Type XI collagen is a heterotrimer but the third alpha chain is a post-translationally modified alpha 1 type II chain. Proteolytic processing of this type XI chain produces PARP, a proline/arginine-rich protein that is an amino terminal domain. Mutations in this gene are associated with type III Stickler syndrome, otospondylomegaepiphyseal dysplasia (OSMED syndrome), Weissenbacher-Zweymuller syndrome, and autosomal dominant nonsyndromic sensorineural 13 deafness. Three transcript variants encoding different isoforms have been identified for this gene. | Extracellular matrix structural protein | Mesoderm development | Integrin signalling pathway->Collagen |
| ***COL2A1*** | 1280 | 12q13.11 | Collagen, type II, alpha 1 (primary osteoarthritis, spondyloepiphyseal dysplasia, congenital). Alpha 1 type II collagen isoform 1. This gene encodes the alpha-1 chain of type II collagen, a fibrillar collagen found in cartilage and the vitreous humor of the eye. Mutations in this gene are associated with achondrogenesis, chondrodysplasia, early onset familial osteoarthritis, SED congenita, Langer-Saldino achondrogenesis, Kniest dysplasia, Stickler syndrome type I, and spondyloepimetaphyseal dysplasia Strudwick type. In addition, defects in processing chondrocalcin, a calcium binding protein that is the C-propeptide of this collagen molecule, are also associated with chondrodysplasia. There are two transcripts identified for this gene. | Extracellular matrix structural protein | Cell adhesion; Cell structure | Integrin signalling pathway->Collagen |
| ***CORS26*** | 81699 | 5p13-p12 | Same as C1QTNF3. This record was replaced with GeneID: 114899. C1q and tumor necrosis factor related protein 3 | Complement component | Complement-mediated immunity | N/A |
| ***CRABP1*** | 1381 | 15q24 | Cellular retinoic acid binding protein 1. A number of specific carrier proteins for members of the vitamin A family have been discovered. Cellular retinoic acid-binding protein is assumed to play an important role in retinoic acid-mediated differentiation and proliferation processes. CRABP1 is structurally similar to the cellular retinol-binding proteins, but binds only retinoic acid. CRABP1 is constitutively expressed and is believed to have different functions in the cell than the related CRABP2. | Other transfer/carrier protein | Lipid and fatty acid transport; Lipid and fatty acid binding; Vitamin/cofactor transport; Steroid hormone-mediated signaling; Transport; Ectoderm development | N/A |
| ***CRELD1*** | 78987 | 3p25.3 | Cysteine-rich with EGF-like domains 1. Epidermal growth factor (EGF; MIM 131530)-like repeats are a class of cysteine-rich domains that mediate interactions between proteins of diverse function. EGF domains are found in proteins that are either completely secreted or have transmembrane regions that tether the protein to the cell surface. CRELD1 is the founding member of a family of matricellular proteins. | Molecular function unclassified | Biological process unclassified | N/A |
| ***CTH*** | 1491 | 1p31.1 | Cystathionase (cystathionine gamma-lyase). This gene encodes a cytoplasmic enzyme in the trans-sulfuration pathway that converts cystathione derived from methionine into cysteine. Glutathione synthesis in the liver is dependent upon the availability of cysteine. Mutations in this gene cause cystathioninuria. Alternative splicing of this gene results in two transcript variants encoding different isoforms. | Other lyase | Amino acid metabolism | N/A |
| ***CTNNB1*** | 1499 | 3p22.1 | Catenin (cadherin-associated protein), beta 1, 88kDa. B-catenin. Beta-catenin is an adherens junction protein. Adherens junctions (AJs; also called the zonula adherens) are critical for the establishment and maintenance of epithelial layers, such as those lining organ surfaces. AJs mediate adhesion between cells, communicate a signal that neighboring cells are present, and anchor the actin cytoskeleton. In serving these roles, AJs regulate normal cell growth and behavior. At several stages of embryogenesis, wound healing, and tumor cell metastasis, cells form and leave epithelia. This process, which involves the disruption and reestablishment of epithelial cell-cell contacts, may be regulated by the disassembly and assembly of AJs. AJs may also function in the transmission of the 'contact inhibition' signal, which instructs cells to stop dividing once an epithelial sheet is complete. | Other signaling molecule; Cell adhesion molecule; Cytoskeletal protein | Cell adhesion-mediated signaling | Angiogenesis->Beta-catenin; Cadherin signaling pathway->Beta-catenin; Wnt signaling pathway->Beta-Catenin |
| ***CUX2*** | 23316 | 12q24.11 | Homeobox protein Cux-2 (Cut-like 2). | Homeobox transcription factor; Nucleic acid binding | mRNA transcription regulation; Developmental processes | N/A |
| ***CX43*** | 2697 | [6q21-q23.2](http://www.ncbi.nlm.nih.gov/mapview/maps.cgi?ORG=hum&chr=6&maps=cntg-r,clone,model,ugHs,loc&VERBOSE=ON&cmd=focus&fill=40&size=40&query=GJA1%5BSYM%5D) | Same as GJA1: gap junction protein, alpha 1, 43kDa (connexin 43). This gene is a member of the connexin gene family. The encoded protein is a component of gap junctions, which are composed of arrays of intercellular channels that provide a route for the diffusion of low molecular weight materials from cell to cell. The encoded protein is the major protein of gap junctions in the heart that are thought to have a crucial role in the synchronized contraction of the heart and in embryonic development. A related intronless pseudogene has been mapped to chromosome 5. Mutations in this gene have been associated with oculodentodigital dysplasia and heart malformations. | Gap junction | Signal transduction | N/A |
| ***CXORF5*** | 8481 | Xp22.2-p22.3 | Same as OFD1 or oral-facial-digital syndrome 1. Human chromosomal region Xp22.3-p21.3 comprises the area between the pseudoautosomal boundary and the Duchenne muscular dystrophy gene (MIM 300377). This region harbors several disease loci, including OFD1 (MIM 311200), CFNS (MIM 304110), DFN6 (MIM 300066), and SEDT (MIM 313400). It also contains a region of homology with both the short and the long arms of the Y chromosome and undergoes frequent chromosomal rearrangements.. Human chromosomal region Xp22.3-p21.3 comprises the area between the pseudoautosomal boundary and the Duchenne muscular dystrophy gene (MIM 300377). This region harbors several disease loci, including OFD1 (MIM 311200), CFNS (MIM 304110), DFN6 (MIM 300066), and SEDT (MIM 313400). It also contains a region of homology with both the short and the long arms of the Y chromosome and undergoes frequent chromosomal rearrangements. | Molecular function unclassified | Biological process unclassified | N/A |
| ***CYP1A1*** | 1543 | 15q24.1 | Cytochrome P450, family 1, subfamily A, polypeptide 1. This gene, CYP1A1, encodes a member of the cytochrome P450 superfamily of enzymes. The cytochrome P450 proteins are monooxygenases which catalyze many reactions involved in drug metabolism and synthesis of cholesterol, steroids and other lipids. This protein localizes to the endoplasmic reticulum and its expression is induced by some polycyclic aromatic hydrocarbons (PAHs), some of which are found in cigarette smoke. The enzyme's endogenous substrate is unknown; however, it is able to metabolize some PAHs to carcinogenic intermediates. The gene has been associated with lung cancer risk. A related family member, CYP1A2, is located approximately 25 kb away from CYP1A1 on chromosome 15. | Oxygenase | Fatty acid metabolism; Steroid metabolism; Electron transport | N/A |
| ***CYP1A2*** | 1544 | 15q24.1 | Cytochrome P450, family 1, subfamily A, polypeptide 2. This gene encodes a member of the cytochrome P450 superfamily of enzymes. The cytochrome P450 proteins are monooxygenases which catalyze many reactions involved in drug metabolism and synthesis of cholesterol, steroids and other lipids. The protein encoded by this gene localizes to the endoplasmic reticulum and its expression is induced by some polycyclic aromatic hydrocarbons (PAHs), some of which are found in cigarette smoke. The enzyme's endogenous substrate is unknown; however, it is able to metabolize some PAHs to carcinogenic intermediates. Other xenobiotic substrates for this enzyme include caffeine, aflatoxin B1, and acetaminophen. The transcript from this gene contains four Alu sequences flanked by direct repeats in the 3' untranslated region. | Oxygenase | Fatty acid metabolism; Steroid metabolism; Electron transport | N/A |
| ***CYP1B1*** | 1545 | 2p22.2 | Cytochrome P450, family 1, subfamily B, polypeptide 1. This gene encodes a member of the cytochrome P450 superfamily of enzymes. The cytochrome P450 proteins are monooxygenases which catalyze many reactions involved in drug metabolism and synthesis of cholesterol, steroids and other lipids. The enzyme encoded by this gene localizes to the endoplasmic reticulum and metabolizes procarcinogens such as polycyclic aromatic hydrocarbons and 17beta-estradiol. Mutations in this gene have been associated with primary congenital glaucoma; therefore it is thought that the enzyme also metabolizes a signaling molecule involved in eye development, possibly a steroid. | Oxygenase | Fatty acid metabolism; Steroid metabolism; Electron transport | N/A |
| ***CYP2D6*** | 1565 | 22q13.2 | Cytochrome P450, family 2, subfamily D, polypeptide 6. This gene encodes a member of the cytochrome P450 superfamily of enzymes. The cytochrome P450 proteins are monooxygenases which catalyze many reactions involved in drug metabolism and synthesis of cholesterol, steroids and other lipids. This protein localizes to the endoplasmic reticulum and is known to metabolize as many as 20% of commonly prescribed drugs. Its substrates include debrisoquine, an adrenergic-blocking drug; sparteine and propafenone, both anti-arrythmic drugs; and amitryptiline, an anti-depressant. The gene is highly polymorphic in the population; certain alleles result in the poor metabolizer phenotype, characterized by a decreased ability to metabolize the enzyme's substrates. The gene is located near two cytochrome P450 pseudogenes on chromosome 22q13.1. Alternatively spliced transcript variants encoding different isoforms have been found for this gene. | Oxygenase | Other lipid, fatty acid and steroid metabolism; Steroid metabolism; Electron transport | Vitamin D metabolism and pathway->Vitamin D 25-Hydroxylase |
| ***CYP2E1*** | 1571 | 10q26.3 | Cytochrome P450, family 2, subfamily E, polypoptide 1. This gene encodes a member of the cytochrome P450 superfamily of enzymes. The cytochrome P450 proteins are monooxygenases which catalyze many reactions involved in drug metabolism and synthesis of cholesterol, steroids and other lipids. This protein localizes to the endoplasmic reticulum and is induced by ethanol, the diabetic state, and starvation. The enzyme metabolizes both endogenous substrates, such as ethanol, acetone, and acetal, as well as exogenous substrates including benzene, carbon tetrachloride, ethylene glycol, and nitrosamines which are premutagens found in cigarette smoke. Due to its many substrates, this enzyme may be involved in such varied processes as gluconeogenesis, hepatic cirrhosis, diabetes, and cancer. | Oxygenase | Fatty acid metabolism; Steroid metabolism; Electron transport | N/A |
| ***CYP3A7*** | 1551 | 7q22.1 | Cytochrome P450, family 3, subfamily A, polypeptide 7. This gene, CYP3A7, encodes a member of the cytochrome P450 superfamily of enzymes. The cytochrome P450 proteins are monooxygenases which catalyze many reactions involved in drug metabolism and synthesis of cholesterol, steroids and other lipids. This enzyme hydroxylates testosterone and dehydroepiandrosterone 3-sulphate, which is involved in the formation of estriol during pregnancy. The enzyme also metabolizes some drugs such as aflatoxin B1. This gene is part of a cluster of cytochrome P450 genes on chromosome 7q21.1. Transcript variants have been described, but it is not known whether these transcripts are normally produced. | Oxygenase | Steroid hormone metabolism; Electron transport | N/A |
| ***DHAND*** | 9464 | 4q34.1 | Heart and neural crest derivatives expressed 2. Also called HAND2 or DHAND2. The protein encoded by this gene belongs to the basic helix-loop-helix family of transcription factors. This gene product is one of two closely related family members, the HAND proteins, which are asymmetrically expressed in the developing ventricular chambers and play an essential role in cardiac morphogenesis. Working in a complementary fashion, they function in the formation of the right ventricle and aortic arch arteries, implicating them as mediators of congenital heart disease. In addition, this transcription factor plays an important role in limb and branchial arch development. | Basic helix-loop-helix transcription factor; Nucleic acid binding | mRNA transcription regulation; Angiogenesis; Heart development | N/A |
| ***DHCR24*** | 1718 | 1p33-p31.1 | DHCR24: 24-dehydrocholesterol reductase. This gene encodes a flavin adenine dinucleotide (FAD)-dependent oxidoreductase which catalyzes the reduction of the delta-24 double bond of sterol intermediates during cholesterol biosynthesis. The protein contains a leader sequence that directs it to the endoplasmic reticulum membrane. Missense mutations in this gene have been associated with desmosterolosis. Also, reduced expression of the gene occurs in the temporal cortex of Alzheimer disease patients and overexpression has been observed in adrenal gland cancer cells. | Reductase | Cholesterol metabolism | N/A |
| ***DHCR7*** | 1717 | 11q13.2-q13.5 | Mutational analysis of the DHCR7 gene led to the identification of seven distinct mutations, three of which are new (F174S, H301R, and Q98X) for Smith-Lemli-Opitz syndrome. | Reductase | Lipid, fatty acid and steroid metabolism | N/A |
| ***DHFR*** | 1719 | 5q11.2-q13.2 | Dihydrofolate reductase. This gene converts dihydrofolate into tetrahydrofolate, a methyl group shuttle required for the de novo synthesis of purines, thymidylic acid, and certain amino acids. While the functional dihydrofolate reductase gene has been mapped to chromosome 5, multiple intronless processed pseudogenes or dihydrofolate reductase-like genes have been identified on separate chromosomes. Dihydrofolate reductase deficiency has been linked to megaloblastic anemia. | Molecular function unclassified | Pyrimidine metabolism; DNA metabolism; Other carbon metabolism | Formyltetrahydroformate biosynthesis->Dihydrofolate reductase; Folate biosynthesis->Dihydrofolate reductase; Folate biosynthesis->Dihydrofolate synthase |
| ***DKK1*** | 22943 | 10q11.2 | Dickkopf homolog 1 (Xenopus laevis). This gene encodes a protein that is a member of the dickkopf family. It is a secreted protein with two cysteine rich regions and is involved in embryonic development through its inhibition of the WNT signaling pathway. Elevated levels of DKK1 in bone marrow plasma and peripheral blood is associated with the presence of osteolytic bone lesions in patients with multiple myeloma. | Molecular function unclassified | Biological process unclassified | N/A |
| ***DLX1*** | 1745 | 2q31.1 | Distal-less homeo box 1. This gene encodes a member of a homeobox transcription factor gene family similiar to the Drosophila distal-less gene. The encoded protein is localized to the nucleus where it may function as a transcriptional regulator of signals from multiple TGF-{beta} superfamily members. The encoded protein may play a role in the control of craniofacial patterning and the differentiation and survival of inhibitory neurons in the forebrain. This gene is located in a tail-to-tail configuration with another member of the family on the long arm of chromosome 2. Alternatively spliced transcript variants encoding different isoforms have been described. | Homeobox transcription factor; Other DNA-binding protein | mRNA transcription regulation; Segment specification; Skeletal development; Muscle development | N/A |
| ***DLX2*** | 1746 | 2q31.1 | Distal-less homeo box 2. Many vertebrate homeo box-containing genes have been identified on the basis of their sequence similarity with Drosophila developmental genes. Members of the Dlx gene family contain a homeobox that is related to that of Distal-less (Dll), a gene expressed in the head and limbs of the developing fruit fly. The Distal-less (Dlx) family of genes comprises at least 6 different members, DLX1-DLX6. The DLX proteins are postulated to play a role in forebrain and craniofacial development. This gene is located in a tail-to-tail configuration with another member of the gene family on the long arm of chromosome 2. | Homeobox transcription factor; Other DNA-binding protein | mRNA transcription regulation; Segment specification; Skeletal development; Muscle development | N/A |
| ***DLX3*** | 1747 | 17q21 | Distal-less homeo box 3. Many vertebrate homeo box-containing genes have been identified on the basis of their sequence similarity with Drosophila developmental genes. Members of the Dlx gene family contain a homeobox that is related to that of Distal-less (Dll), a gene expressed in the head and limbs of the developing fruit fly. The Distal-less (Dlx) family of genes comprises at least 6 different members, DLX1-DLX6. Trichodentoosseous syndrome (TDO), an autosomal dominant condition, has been correlated with DLX3 gene mutation. This gene is located in a tail-to-tail configuration with another member of the gene family on the long arm of chromosome 17. Mutations in this gene have been associated with the autosomal dominant conditions trichodentoosseous syndrome and amelogenesis imperfecta with taurodontism. | Homeobox transcription factor; Other DNA-binding protein | mRNA transcription regulation; Segment specification; Skeletal development; Muscle development | N/A |
| ***DLX5*** | 1749 | 7q21.3 | Distal-less homeo box 5. This gene encodes a member of a homeobox transcription factor gene family similiar to the Drosophila distal-less gene. The encoded protein may play a role in bone development and fracture healing. Mutation in this gene, which is located in a tail-to-tail configuration with another member of the family on the long arm of chromosome 7, may be associated with split-hand/split-foot malformation. | Homeobox transcription factor; Other DNA-binding protein | mRNA transcription regulation; Segment specification; Skeletal development; Muscle development | N/A |
| ***DLX6*** | 1750 | 7q21.3 | Distal-less homeo box 6. This gene encodes a member of a homeobox transcription factor gene family similiar to the Drosophila distal-less gene. This family is comprised of at least 6 different members that encode proteins with roles in forebrain and craniofacial development. This gene is in a tail-to-tail configuration with another member of the family on the long arm of chromosome 7. | Homeobox transcription factor; Other DNA-binding protein | mRNA transcription regulation; Segment specification; Skeletal development; Muscle development | N/A |
| ***DLX7*** | 1748 | 17q21.33 | Distal-less homeo box 7. Same as DLX4. Many vertebrate homeo box-containing genes have been identified on the basis of their sequence similarity with Drosophila developmental genes. Members of the Dlx gene family contain a homeobox that is related to that of Distal-less (Dll), a gene expressed in the head and limbs of the developing fruit fly. The Distal-less (Dlx) family of genes comprises at least 6 different members, DLX1-DLX6. The DLX proteins are postulated to play a role in forebrain and craniofacial development. Three transcript variants have been described for this gene, however, the full length nature of one variant has not been described. Studies of the two splice variants revealed that one encoded isoform functions as a repressor of the beta-globin gene while the other isoform lacks that function. | Homeobox transcription factor; Other DNA-binding protein | mRNA transcription regulation; Segment specification; Skeletal development; Muscle development | N/A |
| ***DMGDH*** | 29958 | 5q14.1 | Dimethylglycine dehydrogenase. This gene encodes an enzyme involved in the catabolism of choline, catalyzing the oxidative demethylation of dimethylglycine to form sarcosine. The enzyme is found as a monomer in the mitochondrial matrix, and uses flavin adenine dinucleotide and folate as cofactors. Mutation in this gene causes dimethylglycine dehydrogenase deficiency, characterized by a fishlike body odor, chronic muscle fatigue, and elevated levels of the muscle form of creatine kinase in serum. | Dehydrogenase | Protein metabolism and modification; Electron transport; Other metabolism | N/A |
| ***DSP*** | 1832 | 6p24 | Desmoplakin. Desmosomes are intercellular junctions that tightly link adjacent cells. Desmoplakin is an obligate component of functional desmosomes that anchors intermediate filaments to desmosomal plaques. The N-terminus of desmoplakin is required for localization to the desmosome and interacts with the N-terminal region of plakophilin 1 and plakoglobin. The C-terminus of desmoplakin binds with intermediate filaments. In the mid-region of desmoplakin, a coiled-coiled rod domain is responsible for homodimerization. Mutations in this gene are the cause of several cardiomyopathies and keratodermas as well as the autoimmune disease paraneoplastic pemphigus. | Molecular function unclassified | Biological process unclassified | N/A |
| ***DTDST*** | 1836 | 5q33.1 | Same as SLC26A2 or solute carrier family 26 (sulfate transporter), member 2. The diastrophic dysplasia sulfate transporter is a transmembrane glycoprotein implicated in the pathogenesis of several human chondrodysplasias. It apparently is critical in cartilage for sulfation of proteoglycans and matrix organization. | Other transporter | Other sulfur metabolism; Anion transport; Extracellular transport and import | N/A |
| ***DVL1*** | 1855 | 1p36 | Dishevelled, dsh homolog 1 (Drosophila). This gene encodes a cytoplasmic phosphoprotein that regulates cell proliferation, acting as a transducer molecule for developmental processes, including segmentation and neuroblast specification. DVL1 is a candidate gene for neuroblastomatous transformation. The Schwartz-Jampel syndrome and Charcot-Marie-Tooth disease type 2A have been mapped to the same region as DVL1. The phenotypes of these diseases may be consistent with defects which might be expected from aberrant expression of a DVL gene during development. Three transcript variants encoding three different isoforms have been found for this gene. | Signaling molecule; Other enzyme regulator | Other receptor mediated signaling pathway; Other intracellular signaling cascade; Neurogenesis | Wnt signaling pathway->Dishevelled |
| ***EDN1*** | 1906 | 6p24.1 | Endothelin 1 | Peptide hormone | G-protein mediated signaling; Ligand-mediated signaling; Regulation of vasoconstriction, dilation; Cell proliferation and differentiation; Homeostasis | Wnt signaling pathway->NFAT Target Genes; Endothelin signaling pathway |
| ***EFNB1*** | 1947 | Xq12 | Ephrin-B1. This gene encodes a member of the ephrin family. The encoded protein is a type I membrane protein and a ligand of Eph-related receptor tyrosine kinases. It may play a role in cell adhesion and function in the development or maintenance of the nervous system. | Membrane-bound signaling molecule | Receptor protein tyrosine kinase signaling pathway; Ligand-mediated signaling; Neurogenesis | Angiogenesis->Ephrin |
| ***EGF*** | 1950 | 4q25 | Epidermal growth factor (beta-urogastrone). EGF has a profound effect on the differentiation of specific cells in vivo and is a potent mitogenic factor for a variety of cultured cells of both ectodermal and mesodermal origin. The EGF precursor is believed to exist as a membrane-bound molecule which is proteolytically cleaved to generate the 53-amino acid peptide hormone that stimulates cells to divide. | Growth factor | Nucleoside, nucleotide and nucleic acid metabolism; Receptor protein tyrosine kinase signaling pathway; Ligand-mediated signaling; Cell cycle control; Cell proliferation and differentiation | N/A |
| ***EGFR*** | 1956 | 7p11.2 | Epidermal growth factor receptor (erythroblastic leukemia viral (v-erb-b) oncogene homolog, avian) | Tyrosine protein kinase receptor; Growth factor; Protein kinase | Protein phosphorylation; Receptor protein tyrosine kinase signaling pathway; Cell cycle control; Cell proliferation and differentiation; Oncogenesis | EGF receptor signaling pathway->EGFR; Cadherin signaling pathway->Epidermal growth factor receptor |
| ***EGR3*** | 1960 | 8p23-p21 | Early growth response 3. The gene encodes a transcriptional regulator that belongs to the EGR family of C2H2-type zinc-finger proteins. It is an immediate-early growth response gene which is induced by mitogenic stimulation. The protein encoded by this gene participates in the transcriptional regulation of genes in controling biological rhythm. It may also plays a role in muscle development. | KRAB box transcription factor; Nucleic acid binding | mRNA transcription regulation; Muscle development | N/A |
| ***EMX2*** | 2018 | 10q26.1 | Empty spiracles homolog 2 (Drosophila). The homeodomain transcription factor EMX2 is critical for central nervous system and urogenital development. EMX1 (MIM 600034) and EMX2 are related to the 'empty spiracles' gene expressed in the developing Drosophila head. | Homeobox transcription factor; Other DNA-binding protein | mRNA transcription regulation; Neurogenesis | N/A |
| ***EPHB2*** | 2048 | 1p36.12 | Ephrin receptor EphB2 isoform 2 precursor. Ephrin receptors and their ligands, the ephrins, mediate numerous developmental processes, particularly in the nervous system. Based on their structures and sequence relationships, ephrins are divided into the ephrin-A (EFNA) class, which are anchored to the membrane by a glycosylphosphatidylinositol linkage, and the ephrin-B (EFNB) class, which are transmembrane proteins. The Eph family of receptors are divided into 2 groups based on the similarity of their extracellular domain sequences and their affinities for binding ephrin-A and ephrin-B ligands. Ephrin receptors make up the largest subgroup of the receptor tyrosine kinase (RTK) family. The protein encoded by this gene is a receptor for ephrin-B family members. | Tyrosine protein kinase receptor; Protein kinase | Protein phosphorylation; Receptor protein tyrosine kinase signaling pathway; Neurogenesis; Mesoderm development; Cell proliferation and differentiation | Angiogenesis->Ephrin Receptor |
| ***EPHB3*** | 2049 | 3q27.1 | Ephrin receptor EphB3 precursor. Ephrin receptors and their ligands, the ephrins, mediate numerous developmental processes, particularly in the nervous system. Based on their structures and sequence relationships, ephrins are divided into the ephrin-A (EFNA) class, which are anchored to the membrane by a glycosylphosphatidylinositol linkage, and the ephrin-B (EFNB) class, which are transmembrane proteins. The Eph family of receptors are divided into 2 groups based on the similarity of their extracellular domain sequences and their affinities for binding ephrin-A and ephrin-B ligands. Ephrin receptors make up the largest subgroup of the receptor tyrosine kinase (RTK) family. The protein encoded by this gene is a receptor for ephrin-B family members. | Tyrosine protein kinase receptor; Protein kinase | Protein phosphorylation; Receptor protein tyrosine kinase signaling pathway; Neurogenesis; Mesoderm development; Cell proliferation and differentiation | Angiogenesis->Ephrin Receptor |
| ***EPHX1*** | 2052 | 1q42.12 | Epoxide hydrolase 1, microsomal (xenobiotic). Epoxide hydrolase plays an important role in both the activation and detoxification of exogenous chemicals such as polycyclic aromatic hydrocarbons. | Other hydrolase | Lipid metabolism; Detoxification | N/A |
| ***EPS15*** | 2060 | 1p31-p32 | Epidermal growth factor receptor pathway substrate 15. This gene encodes a protein that is part of the EGFR pathway. The protein is present at clatherin-coated pits and is involved in receptor-mediated endocytosis of EGF. Notably, this gene is rearranged with the HRX/ALL/MLL gene in acute myelogeneous leukemias. Alternate transcriptional splice variants of this gene have been observed but have not been thoroughly characterized. | Other G-protein modulator; Select calcium binding protein; Membrane traffic protein | Endocytosis; Neurotransmitter release | N/A |
| ***ERCC4*** | 2072 | 16p13.3-p13.11 | Excision repair cross-complementing rodent repair deficiency, complementation group 4. Involved inDNA strand incision during nucleotide excision repair. XPF is required to form gamma-H2AX and likely double strand breaks in response to interstrand crosslinks in human cells. | Endodeoxyribonuclease | DNA repair | N/A |
| ***ESR1*** | 2099 | 6q25.1 | Estrogen receptor 1. The estrogen receptor (ESR) is a ligand-activated transcription factor composed of several domains important for hormone binding, DNA binding, and activation of transcription. Alternative splicing results in several ESR1 mRNA transcripts, which differ primarily in their 5-prime untranslated regions. The translated receptors show less variability. | Nuclear hormone receptor; Transcription factor; Nucleic acid binding | mRNA transcription regulation; Steroid hormone-mediated signaling; Other neuronal activity; Oogenesis; Cell cycle control; Mitosis; Cell proliferation and differentiation; Cell motility | N/A |
| ***ESR2*** | 2100 | 14q | Estrogen receptor 2 (ER beta). This gene encodes a member of the family of estrogen receptors and superfamily of nuclear receptor transcription factors. The gene product contains an N-terminal DNA binding domain and C-terminal ligand binding domain and is localized to the nucleus, cytoplasm, and mitochondria. Upon binding to 17beta-estradiol or related ligands, the encoded protein forms homo- or hetero-dimers that interact with specific DNA sequences to activate transcription. Some isoforms dominantly inhibit the activity of other estrogen receptor family members. Several alternatively spliced transcript variants of this gene have been described, but the full-length nature of some of these variants has not been fully characterized. | Nuclear hormone receptor; Transcription factor; Nucleic acid binding | mRNA transcription regulation; Steroid hormone-mediated signaling; Other neuronal activity; Oogenesis; Cell cycle control; Mitosis; Cell proliferation and differentiation; Cell motility | N/A |
| ***ESRRB*** | 2103 | 14q24.3 | Estrogen-related receptor beta. This gene encodes a protein with similarity to the estrogen receptor. Its function is unknown; however, a similar protein in mouse plays an essential role in placental development. | Nuclear hormone receptor; Transcription factor; Nucleic acid binding | Biological process unclassified | N/A |
| ***ETV5*** | 2119 | 3q28 | Ets variant gene 5 (ets-related molecule). Functional role for the ERMs (ezrin/radixin/moesin) as adaptor molecules in the interactions of adhesion receptors and intracellular tyrosine kinases. ERM is subject to SUMO modification and this post-translational modification causes inhibition of transcription-enhancing activity. | Other transcription factor | mRNA transcription; Developmental processes; Oncogene | N/A |
| ***EVC*** | 2121 | 4p16 | Ellis van Creveld syndrome. This gene encodes a protein containing a leucine zipper and a transmembrane domain. This gene has been implicated in both Ellis-van Creveld syndrome (EvC) and Weyers acrodental dysostosis. | Molecular function unclassified | Muscle development | N/A |
| ***EVI1*** | 2122 | 3q24-q28 | Ecotropic viral integration site 1. Evi-1 acts as a general Smad corepressor to inhibit TGF-beta-, activin-, and BMP-inducible transcription. | KRAB box transcription factor | Biological process unclassified | N/A |
| ***EYA1*** | 2138 | 8q13.3 | Eyes absent homolog 1 (Drosophila). This gene encodes a member of the eyes absent (EYA) family of proteins. The encoded protein may play a role in the developing kidney, branchial arches, eye, and ear. Mutations of this gene have been associated with branchiootorenal dysplasia syndrome, branchiootic syndrome, and sporadic cases of congenital cataracts and ocular anterior segment anomalies. A similar protein in mice can act as a transcriptional activator. Four transcript variants encoding three distinct isoforms have been identified for this gene. | Hydrolase | Vision; Developmental processes | N/A |
| ***F13A1*** | 2162 | 6p25.3-p24.3 | Coagulation factor XIII, A1 polypeptide. This gene encodes the coagulation factor XIII A subunit. Coagulation factor XIII is the last zymogen to become activated in the blood coagulation cascade. Plasma factor XIII is a heterotetramer composed of 2 A subunits and 2 B subunits. The A subunits have catalytic function, and the B subunits do not have enzymatic activity and may serve as plasma carrier molecules. Platelet factor XIII is comprised only of 2 A subunits, which are identical to those of plasma origin. Upon cleavage of the activation peptide by thrombin and in the presence of calcium ion, the plasma factor XIII dissociates its B subunits and yields the same active enzyme, factor XIIIa, as platelet factor XIII. This enzyme acts as a transglutaminase to catalyze the formation of gamma-glutamyl-epsilon-lysine crosslinking between fibrin molecules, thus stabilizing the fibrin clot. It also crosslinks alpha-2-plasmin inhibitor, or fibronectin, to the alpha chains of fibrin. Factor XIII deficiency is classified into two categories: type I deficiency, characterized by the lack of both the A and B subunits; and type II deficiency, characterized by the lack of the A subunit alone. These defects can result in a lifelong bleeding tendency, defective wound healing, and habitual abortion. | Other transferase | Blood clotting | Blood coagulation->Factor XIII; Blood coagulation->Factor XIIIaA |
| ***FGF1*** | 2246 | 5q31 | Fibroblast growth factor 1. The protein encoded by this gene is a member of the fibroblast growth factor (FGF) family. FGF family members possess broad mitogenic and cell survival activities, and are involved in a variety of biological processes, including embryonic development, cell growth, morphogenesis, tissue repair, tumor growth and invasion. This protein functions as a modifier of endothelial cell migration and proliferation, as well as an angiogenic factor. It acts as a mitogen for a variety of mesoderm- and neuroectoderm-derived cells in vitro, thus is thought to be involved in organogenesis. Three alternatively spliced variants encoding different isoforms have been described. | Growth factor | Cell surface receptor mediated signal transduction; MAPKKK cascade; Ligand-mediated signaling; Angiogenesis; Cell cycle control; Cell proliferation and differentiation | FGF signaling pathway->fibroblast growth factor; Angiogenesis->Fibroblast Growth Factor |
| ***FGF10*** | 2255 | 5p12 | Fibroblast growth factor 10 precursor. The protein encoded by this gene is a member of the fibroblast growth factor (FGF) family. FGF family members possess broad mitogenic and cell survival activities, and are involved in a variety of biological processes, including embryonic development, cell growth, morphogenesis, tissue repair, tumor growth and invasion. This protein exhibits mitogenic activity for keratinizing epidermal cells, but essentially no activity for fibroblasts, which is similar to the biological activity of FGF7. Studies of the mouse homolog of suggested that this gene is required for embryonic epidermal morphogenesis including brain development, lung morphogenesis, and initiation of lim bud formation. This gene is also implicated to be a primary factor in the process of wound healing. | Growth factor | Receptor protein tyrosine kinase signaling pathway | FGF signaling pathway->fibroblast growth factor |
| ***FGF12*** | 2257 | 3q28 | Fibroblast growth factor 12. The protein encoded by this gene is a member of the fibroblast growth factor (FGF) family. FGF family members possess broad mitogenic and cell survival activities, and are involved in a variety of biological processes, including embryonic development, cell growth, morphogenesis, tissue repair, tumor growth, and invasion. This growth factor lacks the N-terminal signal sequence present in most of the FGF family members, but it contains clusters of basic residues that have been demonstrated to act as a nuclear localization signal. When transfected into mammalian cells, this protein accumulated in the nucleus, but was not secreted. The specific function of this gene has not yet been determined. Two alternatively spliced transcript variants encoding distinct isoforms have been reported. | Growth factor | Receptor protein tyrosine kinase signaling pathway | FGF signaling pathway->fibroblast growth factor |
| ***FGF2*** | 2247 | 4q26-q27 | Fibroblast growth factor 2 (basic). The protein encoded by this gene is a member of the fibroblast growth factor (FGF) family. FGF family members bind heparin and possess broad mitogenic and angiogenic activities. This protein has been implicated in diverse biological processes, such as limb and nervous system development, wound healing, and tumor growth. The mRNA for this gene contains multiple polyadenylation sites, and is alternatively translated from AUG and non-AUG (CUG) initiation codons resulting in five different isoforms with distinct properties. The CUG-initiated isoforms are localized in the nucleus and are responsible for the intracrine effect, whereas, the AUG-initiated form is mostly cytosolic and is responsible for the paracrine and autocrine effects of this FGF. | Growth factor | Cell surface receptor mediated signal transduction; MAPKKK cascade; Ligand-mediated signaling; Angiogenesis; Cell cycle control; Cell proliferation and differentiation | FGF signaling pathway->fibroblast growth factor; Angiogenesis->Fibroblast Growth Factor |
| ***FGF4*** | 2249 | 11q13.3 | Fibroblast growth factor 4 (heparin secretory transforming protein 1, Kaposi sarcoma oncogene). The protein encoded by this gene is a member of the fibroblast growth factor (FGF) family. FGF family members possess broad mitogenic and cell survival activities and are involved in a variety of biological processes including embryonic development, cell growth, morphogenesis, tissue repair, tumor growth and invasion. This gene was identified by its oncogenic transforming activity. This gene and FGF3, another oncogenic growth factor, are located closely on chromosome 11. Co-amplification of both genes was found in various kinds of human tumors. Studies on the mouse homolog suggested a function in bone morphogenesis and limb development through the sonic hedgehog (SHH) signaling pathway. | Growth factor | Receptor protein tyrosine kinase signaling pathway; Developmental processes | FGF signaling pathway->fibroblast growth factor |
| ***FGF5*** | 2250 | 4q21 | Fibroblast growth factor 4 (heparin secretory transforming protein 1, Kaposi sarcoma oncogene). The protein encoded by this gene is a member of the fibroblast growth factor (FGF) family. FGF family members possess broad mitogenic and cell survival activities and are involved in a variety of biological processes including embryonic development, cell growth, morphogenesis, tissue repair, tumor growth and invasion. This gene was identified by its oncogenic transforming activity. This gene and FGF3, another oncogenic growth factor, are located closely on chromosome 11. Co-amplification of both genes was found in various kinds of human tumors. Studies on the mouse homolog suggested a function in bone morphogenesis and limb development through the sonic hedgehog (SHH) signaling pathway. | Growth factor | Receptor protein tyrosine kinase signaling pathway; MAPKKK cascade; Ligand-mediated signaling; Neurogenesis; Cell cycle control; Cell proliferation and differentiation; Oncogene | FGF signaling pathway->fibroblast growth factor |
| ***FGF7*** | 2252 | 15q15-q21.1 | Fibroblast growth factor 7 (keratinocyte growth factor). The protein encoded by this gene is a member of the fibroblast growth factor (FGF) family. FGF family members possess broad mitogenic and cell survival activities, and are involved in a variety of biological processes, including embryonic development, cell growth, morphogenesis, tissue repair, tumor growth and invasion. This protein is a potent epithelial cell-specific growth factor, whose mitogenic activity is predominantly exhibited in keratinocytes but not in fibroblasts and endothelial cells. Studies of mouse and rat homologs of this gene implicated roles in morphogenesis of epithelium, reepithelialization of wounds, hair development and early lung organogenesis. | Growth factor | Cell surface receptor mediated signal transduction; MAPKKK cascade; Ligand-mediated signaling; Ectoderm development; Cell cycle control; Cell proliferation and differentiation; Oncogene | FGF signaling pathway->fibroblast growth factor |
| ***FGF8*** | 2253 | 10q24.32 | Fibroblast growth factor 8 (androgen-induced). The protein encoded by this gene is a member of the fibroblast growth factor (FGF) family. FGF family members possess broad mitogenic and cell survival activities, and are involved in a variety of biological processes, including embryonic development, cell growth, morphogenesis, tissue repair, tumor growth and invasion. This protein is known to be a factor that supports androgen and anchorage independent growth of mammary tumor cells. Overexpression of this gene has been shown to increase tumor growth and angiogensis. The adult expression of this gene is restricted to testes and ovaries. Temporal and spatial pattern of this gene expression suggests its function as an embryonic epithelial factor. Studies of the mouse and chick homologs revealed roles in midbrain and limb development, organogenesis, embryo gastrulation and left-right axis determination. The alternative splicing of this gene results in four transcript variants. | Growth factor | Cell surface receptor mediated signal transduction | FGF signaling pathway->fibroblast growth factor |
| ***FGF9*** | 2254 | 13q11-q12 | Fibroblast growth factor 9 (glia-activating factor). The protein encoded by this gene is a member of the fibroblast growth factor (FGF) family. FGF family members possess broad mitogenic and cell survival activities, and are involved in a variety of biological processes, including embryonic development, cell growth, morphogenesis, tissue repair, tumor growth and invasion. This protein was isolated as a secreted factor that exhibits a growth-stimulating effect on cultured glial cells. In nervous system, this protein is produced mainly by neurons and may be important for glial cell development. Expression of the mouse homolog of this gene was found to be dependent on Sonic hedgehog (Shh) signaling. Mice lacking the homolog gene displayed a male-to-female sex reversal phenotype, which suggested a role in testicular embryogenesis. | Growth factor | Receptor protein tyrosine kinase signaling pathway | FGF signaling pathway->fibroblast growth factor |
| ***FGFBP1*** | 9982 | 4p16-p15 | Fibroblast growth factor binding protein 1. FGFBP1, or HBP17, binds to both acidic (FGF1; MIM 131220) and basic (FGF2; MIM 134920) fibroblast growth factors in a reversible manner. It also binds to perlecan (HSPG2; MIM 142461). | Other miscellaneous function protein | Biological process unclassified | N/A |
| ***FGFR1*** | 2260 | 8p12 | Fibroblast growth factor receptor 1 (fms-related tyrosine kinase 2, Pfeiffer syndrome). The protein encoded by this gene is a member of the fibroblast growth factor receptor (FGFR) family, where amino acid sequence is highly conserved between members and throughout evolution. FGFR family members differ from one another in their ligand affinities and tissue distribution. A full-length representative protein consists of an extracellular region, composed of three immunoglobulin-like domains, a single hydrophobic membrane-spanning segment and a cytoplasmic tyrosine kinase domain. The extracellular portion of the protein interacts with fibroblast growth factors, setting in motion a cascade of downstream signals, ultimately influencing mitogenesis and differentiation. This particular family member binds both acidic and basic fibroblast growth factors and is involved in limb induction. Mutations in this gene have been associated with Pfeiffer syndrome, Jackson-Weiss syndrome, Antley-Bixler syndrome, osteoglophonic dysplasia, and autosomal dominant Kallmann syndrome 2. Chromosomal aberrations involving this gene are associated with stem cell myeloproliferative disorder and stem cell leukemia lymphoma syndrome. Alternatively spliced variants which encode different protein isoforms have been described; however, not all variants have been fully characterized. | Tyrosine protein kinase receptor; Protein kinase | Protein phosphorylation; Receptor protein tyrosine kinase signaling pathway; Neurogenesis; Cell proliferation and differentiation; Other oncogenesis | FGF signaling pathway->FGFR1-4; Angiogenesis->Fibroblast Growth Factor Receptor-1 |
| ***FGFR2*** | 2263 | 10q26.13 | Fibroblast growth factor receptor 2 (bacteria-expressed kinase, keratinocyte growth factor receptor, craniofacial dysostosis 1, Crouzon syndrome, Pfeiffer syndrome, Jackson-Weiss syndrome). The protein encoded by this gene is a member of the fibroblast growth factor receptor family, where amino acid sequence is highly conserved between members and throughout evolution. FGFR family members differ from one another in their ligand affinities and tissue distribution. A full-length representative protein consists of an extracellular region, composed of three immunoglobulin-like domains, a single hydrophobic membrane-spanning segment and a cytoplasmic tyrosine kinase domain. The extracellular portion of the protein interacts with fibroblast growth factors, setting in motion a cascade of downstream signals, ultimately influencing mitogenesis and differentiation. This particular family member is a high-affinity receptor for acidic, basic and/or keratinocyte growth factor, depending on the isoform. Mutations in this gene are associated with Crouzon syndrome, Pfeiffer syndrome, Craniosynostosis, Apert syndrome, Jackson-Weiss syndrome, Beare-Stevenson cutis gyrata syndrome, Saethre-Chotzen syndrome, and syndromic craniosynostosis. Alternatively spliced variants which encode different protein isoforms have been described; however, not all variants have been fully characterized. | Tyrosine protein kinase receptor; Protein kinase | Protein phosphorylation; Receptor protein tyrosine kinase signaling pathway; Neurogenesis; Cell proliferation and differentiation; Other oncogenesis | FGF signaling pathway->FGFR1-4; Angiogenesis->Fibroblast Growth Factor Receptor-1 |
| ***FGFR3*** | 2261 | 4p16.3 | Fibroblast growth factor receptor 3 (achondroplasia, thanatophoric dwarfism). The protein encoded by this gene is a member of the fibroblast growth factor receptor family, where amino acid sequence is highly conserved between members and throughout evolution. FGFR family members differ from one another in their ligand affinities and tissue distribution. A full-length representative protein would consist of an extracellular region, composed of three immunoglobulin-like domains, a single hydrophobic membrane-spanning segment and a cytoplasmic tyrosine kinase domain. The extracellular portion of the protein interacts with fibroblast growth factors, setting in motion a cascade of downstream signals, ultimately influencing mitogenesis and differentiation. This particular family member binds acidic and basic fibroblast growth hormone and plays a role in bone development and maintenance. Mutations in this gene lead to craniosynostosis and multiple types of skeletal dysplasia. Alternative splicing occurs and additional variants have been described, including those utilizing alternate exon 8 rather than 9, but their full-length nature has not been determined. | Tyrosine protein kinase receptor; Protein kinase | Protein phosphorylation; Receptor protein tyrosine kinase signaling pathway; Neurogenesis; Cell proliferation and differentiation; Other oncogenesis | FGF signaling pathway->FGFR1-4; Angiogenesis->Fibroblast Growth Factor Receptor-1 |
| ***FGFR4*** | 2264 | [5q35.1-qter](http://www.ncbi.nlm.nih.gov/mapview/maps.cgi?ORG=hum&chr=5&maps=cntg-r,clone,model,ugHs,loc&VERBOSE=ON&cmd=focus&fill=40&size=40&query=FGFR4%5BSYM%5D) | Fibroblast growth factor receptor 4. The protein encoded by this gene is a member of the fibroblast growth factor receptor family, where amino acid sequence is highly conserved between members and throughout evolution. FGFR family members differ from one another in their ligand affinities and tissue distribution. A full-length representative protein would consist of an extracellular region, composed of three immunoglobulin-like domains, a single hydrophobic membrane-spanning segment and a cytoplasmic tyrosine kinase domain. The extracellular portion of the protein interacts with fibroblast growth factors, setting in motion a cascade of downstream signals, ultimately influencing mitogenesis and differentiation. The genomic organization of this gene, compared to members 1-3, encompasses 18 exons rather than 19 or 20. Although alternative splicing has been observed, there is no evidence that the C-terminal half of the IgIII domain of this protein varies between three alternate forms, as indicated for members 1-3. This particular family member preferentially binds acidic fibroblast growth factor and, although its specific function is unknown, it is overexpressed in gynecological tumor samples, suggesting a role in breast and ovarian tumorigenesis. | Tyrosine protein kinase receptor; Protein kinase | Protein phosphorylation; Receptor protein tyrosine kinase signaling pathway; Neurogenesis; Cell proliferation and differentiation; Other oncogenesis | FGF signaling pathway->FGFR1-4; Angiogenesis->Fibroblast Growth Factor Receptor-1 |
| ***FLNA*** | 2316 | Xq28 | Filamin A, alpha (actin binding protein 280). Actin-binding protein, or filamin, is a 280-kD protein that crosslinks actin filaments into orthogonal networks in cortical cytoplasm and participates in the anchoring of membrane proteins for the actin cytoskeleton. Remodeling of the cytoskeleton is central to the modulation of cell shape and migration. Filamin A, encoded by the FLNA gene, is a widely expressed protein that regulates reorganization of the actin cytoskeleton by interacting with integrins, transmembrane receptor complexes, and second messengers. | Non-motor actin binding protein | Cell structure; Cell motility | Integrin signalling pathway->Filamin; Dopamine receptor mediated signaling pathway->filamin A |
| ***FLNB*** | 2317 | 3p14.3 | Filamin B, beta (actin binding protein 278). Different splice variants of filamin-B affect myogenesis, subcellular distribution, and determine binding to integrin [beta] subunits | Non-motor actin binding protein | Cell structure; Cell motility | Integrin signalling pathway->Filamin; Dopamine receptor mediated signaling pathway->filamin A |
| ***FOLH1*** | 2346 | 11p11.2 | Folate hydrolase (prostate-specific membrane antigen) 1. This gene encodes a type II transmembrane glycoprotein belonging to the M28 peptidase family. The protein acts as a glutamate carboxypeptidase on different alternative substrates, including the nutrient folate and the neuropeptide N-acetyl-l-aspartyl-l-glutamate and is expressed in a number of tissues such as prostate, central and peripheral nervous system and kidney. A mutation in this gene may be associated with impaired intestinal absorption of dietary folates, resulting in low blood folate levels and consequent hyperhomocysteinemia. Expression of this protein in the brain may be involved in a number of pathological conditions associated with glutamate excitotoxicity. In the prostate the protein is up-regulated in cancerous cells and is used as an effective diagnostic and prognostic indicator of prostate cancer. This gene likely arose from a duplication event of a nearby chromosomal region. Alternative splicing gives rise to multiple transcript variants. | Other proteases | Proteolysis | N/A |
| ***FOLR3*** | 2352 | 11q13.4 | Folate receptor 3 (gamma). This gene encodes a member of the folate receptor (FOLR) family, members of which have a high affinity for folic acid and for several reduced folic acid derivatives, and mediate delivery of 5-methyltetrahydrofolate to the interior of cells. This gene includes two polymorphic variants; the shorter one has two base deletion in the CDS, resulting in a truncated polypeptide, compared to the longer one. Both protein products are constitutively secreted in hematopoietic tissues and are potential serum marker for certain hematopoietic malignancies. The longer protein has a 71% and 79% sequence homology with the FOLR1 and FOLR2 proteins, respectively. | Other receptor; Other transporter | Vitamin/cofactor transport; Transport | N/A |
| ***FOLRA*** | 2348 | 11q13.4 | Same as FOLR1 or Folate receptor 1 precursor. The protein encoded by this gene is a member of the folate receptor (FOLR) family. Members of this gene family have a high affinity for folic acid and for several reduced folic acid derivatives, and mediate delivery of 5-methyltetrahydrofolate to the interior of cells. This gene is composed of 7 exons; exons 1 through 4 encode the 5' UTR and exons 4 through 7 encode the open reading frame. Due to the presence of 2 promoters, multiple transcription start sites, and alternative splicing of exons, several transcript variants are derived from this gene. These variants differ in the lengths of 5' and 3' UTR, but they encode an identical amino acid sequence. Same as FOLR1. | Other receptor; Other transporter | Vitamin/cofactor transport; Transport | N/A |
| ***FOLRB*** | 2350 | 11q13.4 | Same as FOLR2 or Folate receptor 2 precursor. The protein encoded by this gene is a member of the folate receptor (FOLR) family. Members of this gene family have a high affinity for folic acid and for several reduced folic acid derivatives, and mediate delivery of 5-methyltetrahydrofolate to the interior of cells. This protein has a 68% and 79% sequence homology with the FOLR1 and FOLR3 proteins, respectively. The FOLR2 protein was originally thought to exist only in placenta, but is also detected in spleen, bone marrow, and thymus. | Other receptor; Other transporter | Vitamin/cofactor transport; Transport | N/A |
| ***FOXC2*** | 2303 | 16q24.1 | Forkhead box C2 (MFH-1, mesenchyme forkhead 1). This gene belongs to the forkhead family of transcription factors which is characterized by a distinct DNA-binding forkhead domain. The specific function of this gene has not yet been determined; however, it may play a role in the development of mesenchymal tissues. | Other transcription factor; Nucleic acid binding | Carbohydrate metabolism; mRNA transcription regulation; Other receptor mediated signaling pathway; Cell communication; Vision; Embryogenesis; Anterior/posterior patterning; Segment specification; Neurogenesis; Mesoderm development; Cell cycle control; Cell proliferation and differentiation; Cell structure | Insulin/IGF pathway-protein kinase B signaling cascade->Forkhead transcription factor; PI3 kinase pathway->FOXO; Interleukin signaling pathway->Forkhead in Rhabdomyosarcoma-like 1; TGF-beta signaling pathway->Co-activators or corepressors |
| ***FOXE1*** | 2304 | 9q22 | Forkhead box E1 (thyroid transcription factor 2). This intronless gene belongs to the forkhead family of transcription factors, which is characterized by a distinct forkhead domain. This gene functions as a thyroid transcription factor which likely plays a crucial role in thyroid morphogenesis. Mutations in this gene are associated with congenital hypothyroidism and cleft palate with thyroid dysgenesis. The map localization of this gene suggests it may also be a candidate gene for squamous cell epithelioma and hereditary sensory neuropathy type I. | Other transcription factor; Nucleic acid binding | Carbohydrate metabolism; mRNA transcription regulation; Other receptor mediated signaling pathway; Cell communication; Vision; Embryogenesis; Anterior/posterior patterning; Segment specification; Neurogenesis; Mesoderm development; Cell cycle control; Cell proliferation and differentiation; Cell structure | Insulin/IGF pathway-protein kinase B signaling cascade->Forkhead transcription factor; PI3 kinase pathway->FOXO; Interleukin signaling pathway->Forkhead in Rhabdomyosarcoma-like 1; TGF-beta signaling pathway->Co-activators or corepressors |
| ***FOXF2*** | 2295 | 6p25.3 | Forkhead box F2. FOXF2 encodes forkhead box F2, one of many human homologues of the Drosophila melanogaster transcription factor forkhead. FOXF2 is expressed in lung and placenta, and has been shown to transcriptionally activate several lung-specific genes. | Other transcription factor; Nucleic acid binding | Carbohydrate metabolism; mRNA transcription regulation; Other receptor mediated signaling pathway; Cell communication; Vision; Embryogenesis; Anterior/posterior patterning; Segment specification; Neurogenesis; Mesoderm development; Cell cycle control; Cell proliferation and differentiation; Cell structure | Insulin/IGF pathway-protein kinase B signaling cascade->Forkhead transcription factor; PI3 kinase pathway->FOXO; Interleukin signaling pathway->Forkhead in Rhabdomyosarcoma-like 1; TGF-beta signaling pathway->Co-activators or corepressors |
| ***FOXH1*** | 8928 | 8q24.3 | Forkhead box H1. FOXH1 encodes a human homolog of Xenopus forkhead activin signal transducer-1. FOXH1 protein binds SMAD2 and activates an activin response element via binding the DNA motif TGT(G/T)(T/G)ATT. | Other transcription factor; Nucleic acid binding | Carbohydrate metabolism; mRNA transcription regulation; Other receptor mediated signaling pathway; Cell communication; Vision; Embryogenesis; Anterior/posterior patterning; Segment specification; Neurogenesis; Mesoderm development; Cell cycle control; Cell proliferation and differentiation; Cell structure | Insulin/IGF pathway-protein kinase B signaling cascade->Forkhead transcription factor; PI3 kinase pathway->FOXO; Interleukin signaling pathway->Forkhead in Rhabdomyosarcoma-like 1; TGF-beta signaling pathway->Co-activators or corepressors |
| ***FOXN1*** | 8456 | 17q11-q12 | Forkhead box N1. Mutations in the winged-helix transcription factor gene at the nude locus in mice and rats produce the pleiotropic phenotype of hairlessness and athymia, resulting in a severely compromised immune system. This gene is orthologous to the mouse and rat genes and encodes a similar DNA-binding transcription factor that is thought to regulate keratin gene expression. A mutation in this gene has been correlated with T-cell immunodeficiency, the skin disorder congenital alopecia, and nail dystrophy. Alternative splicing in the 5' UTR of this gene has been observed. | Other transcription factor; Nucleic acid binding | Carbohydrate metabolism; mRNA transcription regulation; Other receptor mediated signaling pathway; Cell communication; Vision; Embryogenesis; Anterior/posterior patterning; Segment specification; Neurogenesis; Mesoderm development; Cell cycle control; Cell proliferation and differentiation; Cell structure | Insulin/IGF pathway-protein kinase B signaling cascade->Forkhead transcription factor; PI3 kinase pathway->FOXO; Interleukin signaling pathway->Forkhead in Rhabdomyosarcoma-like 1; TGF-beta signaling pathway->Co-activators or corepressors |
| ***FOXP2*** | 93986 | 7q31 | Forkhead box P2. This gene encodes an evolutionarily conserved transcription factor expressed in fetal and adult brain. This transcription factor is a member of the forkhead/winged-helix (FOX) family of transcription factors, and contains a FOX DNA-binding domain and a large polyglutamine tract. Members of the FOX family of transcription factors are regulators of embryogenesis. The product of this gene is thought to be required for proper development of speech and language regions of the brain during embryogenesis. Although a point mutation in this gene has been associated with the KE pedigree segregating developmental verbal dyspraxia, no association between mutations in this gene and another speech disorder, autism, has been found. Four alternative transcripts encoding three different isoforms have been identified. | Other transcription factor; Nucleic acid binding | Carbohydrate metabolism; mRNA transcription regulation; Other receptor mediated signaling pathway; Cell communication; Vision; Embryogenesis; Anterior/posterior patterning; Segment specification; Neurogenesis; Mesoderm development; Cell cycle control; Cell proliferation and differentiation; Cell structure | Insulin/IGF pathway-protein kinase B signaling cascade->Forkhead transcription factor; PI3 kinase pathway->FOXO; Interleukin signaling pathway->Forkhead in Rhabdomyosarcoma-like 1; TGF-beta signaling pathway->Co-activators or corepressors |
| ***FRAS1*** | 80144 | 4q21.21 | Fraser syndrome 1. The FRAS1 gene encodes a putative extracellular matrix (ECM) protein and is mutated in Fraser syndrome (MIM 219000). | Molecular function unclassified | Biological process unclassified | N/A |
| ***FSCN1*** | 6624 | 7p22 | Fascin homolog 1, actin-bundling protein (Strongylocentrotus purpuratus). | Non-motor actin binding protein | Oncogenesis; Cell motility | N/A |
| ***FST*** | 10468 | 5q11.2 | Follistatin. Follistatin is a single-chain gonadal protein that specifically inhibits follicle-stimulating hormone release. The single FST gene encodes two isoforms, FST317 and FST344 containing 317 and 344 amino acids respectively, resulting from alternative splicing of the precursor mRNA. In a study in which 37 candidate genes were tested for linkage and association with polycystic ovary syndrome (PCOS) or hyperandrogenemia in 150 families, evidence was found for linkage between PCOS and follistatin. | Select regulatory molecule | Homeostasis | N/A |
| ***FTCD*** | 10841 | 21q22.3 | Formiminotransferase cyclodeaminase. FTCD is a bifunctional enzyme that channels 1-carbon units from formiminoglutamate, a metabolite of the histidine degradation pathway, to the folate pool. | Other transferase; Deaminase | Amino acid catabolism | N/A |
| ***FTHFD*** | 10840 | 3q21.2 | Same as ALDH1L1 or aldehyde dehydrogenase 1 family, member L1. Formyltetrahydrofolate dehydrogenase, same as ALDH1L1, or aldehyde dehydrogenase 1 family, member L1. The protein encoded by this gene catalyzes the conversion of 10-formyltetrahydrofolate, NADP, and water to tetrahydrofolate, NADPH, and carbon dioxide. The encoded protein belongs to the aldehyde dehydrogenase family and is responsible for formate oxidation in vivo. Deficiencies in this gene can result in an accumulation of formate and subsequent methanol poisoning. | Dehydrogenase | Other carbon metabolism | 5-Hydroxytryptamine degredation->Aldehyde Dehydrogenase; Phenylethylamine degradation->Phenylacetaldehyde dehydrogenase |
| ***FZD1*** | 8321 | 7q21 | Frizzled homolog 1 (Drosophila). Members of the 'frizzled' gene family encode 7-transmembrane domain proteins that are receptors for Wnt signaling proteins. The FZD1 protein contains a signal peptide, a cysteine-rich domain in the N-terminal extracellular region, 7 transmembrane domains, and a C-terminal PDZ domain-binding motif. The FZD1 transcript is expressed in various tissues. | Molecular function unclassified | Biological process unclassified | Angiogenesis->Frizzled; Alzheimer disease-presenilin pathway->Frizzled; Angiogenesis->Frizzled-Related Protein; Cadherin signaling pathway->Frizzled; Wnt signaling pathway->Frizzled |
| ***FZD10*** | 11211 | 12q24.33 | Frizzled homolog 10 (Drosophila). This gene is a member of the frizzled gene family. Members of this family encode 7-transmembrane domain proteins that are receptors for the Wingless type MMTV integration site family of signaling proteins. Most frizzled receptors are coupled to the beta-catenin canonical signaling pathway. Using array analysis, expression of this intronless gene is significantly up-regulated in two cases of primary colon cancer. | Molecular function unclassified | Biological process unclassified | Alzheimer disease-presenilin pathway->Frizzled; Cadherin signaling pathway->Frizzled; Wnt signaling pathway->Frizzled |
| ***FZD2*** | 2535 | 17q21.31 | Frizzled homolog 2 (Drosophila). Members of the 'frizzled' gene family encode 7-transmembrane domain proteins that are receptors for Wnt signaling proteins. The expression of the FZD2 gene appears to be developmentally regulated, with high levels of expression in fetal kidney and lung and in adult colon and ovary. | Molecular function unclassified | Biological process unclassified | Angiogenesis->Frizzled; Alzheimer disease-presenilin pathway->Frizzled; Cadherin signaling pathway->Frizzled; Wnt signaling pathway->Frizzled |
| ***FZD4*** | 8322 | 11q14.2 | Frizzled homolog 4 (Drosophila). This gene is a member of the frizzled gene family. Members of this family encode seven-transmembrane domain proteins that are receptors for the Wingless type MMTV integration site family of signaling proteins. Most frizzled receptors are coupled to the beta-catenin canonical signaling pathway. This protein may play a role as a positive regulator of the Wingless type MMTV integration site signaling pathway. A transcript variant retaining intronic sequence and encoding a shorter isoform has been described; however, its expression is not supported by other experimental evidence. | Molecular function unclassified | Biological process unclassified | Alzheimer disease-presenilin pathway->Frizzled; Cadherin signaling pathway->Frizzled; Wnt signaling pathway->Frizzled |
| ***FZD7*** | 8324 | 2q33.1 | Frizzled homolog 7 (Drosophila). Members of the 'frizzled' gene family encode 7-transmembrane domain proteins that are receptors for Wnt signaling proteins. The FZD7 protein contains an N-terminal signal sequence, 10 cysteine residues typical of the cysteine-rich extracellular domain of Fz family members, 7 putative transmembrane domains, and an intracellular C-terminal tail with a PDZ domain-binding motif. FZD7 gene expression may downregulate APC function and enhance beta-catenin-mediated signals in poorly differentiated human esophageal carcinomas. | Molecular function unclassified | Biological process unclassified | Alzheimer disease-presenilin pathway->Frizzled; Cadherin signaling pathway->Frizzled; Wnt signaling pathway->Frizzled |
| ***FZD8*** | 8325 | 10p11.21 | Frizzled homolog 8 (Drosophila). This intronless gene is a member of the frizzled gene family. Members of this family encode seven-transmembrane domain proteins that are receptors for the Wingless type MMTV integration site family of signaling proteins. Most frizzled receptors are coupled to the beta-catenin canonical signaling pathway. This gene is highly expressed in two human cancer cell lines, indicating that it may play a role in several types of cancer. The crystal structure of the extracellular cysteine-rich domain of a similar mouse protein has been determined. | Molecular function unclassified | Biological process unclassified | Angiogenesis->Frizzled; Alzheimer disease-presenilin pathway->Frizzled; Cadherin signaling pathway->Frizzled; Wnt signaling pathway->Frizzled |
| ***GABRB3*** | 2562 | 15q12 | Gamma-aminobutyric acid (GABA) A receptor, beta 3. This gene encodes a member of the ligand-gated ionic channel family. The encoded protein is one of at least 13 distinct subunits of a multisubunit chloride channel that serves as the receptor for gamma-aminobutyric acid, the major inhibitory transmitter of the nervous system. This gene is located on the long arm of chromosome 15 in a cluster with two genes encoding related subunits of the family. Mutations in this gene may be associated with the pathogenesis of Angelman syndrome, Prader-Willi syndrome, and autism. Alternatively spliced transcript variants encoding isoforms with distinct signal peptides have been described. | GABA receptor; Ion channel | Ligand-mediated signaling; Anion transport; Nerve-nerve synaptic transmission | N/A |
| ***GAD1*** | 2571 | 2q31 | Glutamate decarboxylase 1 (brain, 67kDa). This gene encodes one of several forms of glutamic acid decarboxylase, identified as a major autoantigen in insulin-dependent diabetes. The enzyme encoded is responsible for catalyzing the production of gamma-aminobutyric acid from L-glutamic acid. A pathogenic role for this enzyme has been identified in the human pancreas since it has been identified as an autoantigen and an autoreactive T cell target in insulin-dependent diabetes. This gene may also play a role in the stiff man syndrome. Deficiency in this enzyme has been shown to lead to pyridoxine dependency with seizures. Alternative splicing of this gene results in two products, the predominant 67-kD form and a less-frequent 25-kD form. | Decarboxylase | Other amino acid metabolism | Gamma-aminobutyric acid synthesis->GAD |
| ***GAD2*** | 2572 | 10p11.23 | Glutamate decarboxylase 2 (pancreatic islets and brain, 65kDa). This gene encodes one of several forms of glutamic acid decarboxylase, identified as a major autoantigen in insulin-dependent diabetes. The enzyme encoded is responsible for catalyzing the production of gamma-aminobutyric acid from L-glutamic acid. A pathogenic role for this enzyme has been identified in the human pancreas since it has been identified as an autoantibody and an autoreactive T cell target in insulin-dependent diabetes. This gene may also play a role in the stiff man syndrome. | Decarboxylase | Other amino acid metabolism | Gamma-aminobutyric acid synthesis->GAD |
| ***GART*** | 2618 | 21q22.1 | Phosphoribosylglycinamide formyltransferase, phosphoribosylglycinamide synthetase, phosphoribosylaminoimidazole synthetase. The protein encoded by this gene is a trifunctional polypeptide. It has phosphoribosylglycinamide formyltransferase, phosphoribosylglycinamide synthetase, phosphoribosylaminoimidazole synthetase activity which is required for de novo purine biosynthesis. This enzyme is highly conserved in vertebrates. Alternative splicing of this gene results in two transcript variants encoding different isoforms. | Other ligase | Purine metabolism | De novo purine biosynthesis->Phosphoribosylamine glycine ligase |
| ***GDF1*** | 2657 | 19p12 | Growth differentiation factor 1. This gene encodes a member of the bone morphogenetic protein (BMP) family and the TGF-beta superfamily. This group of proteins is characterized by a polybasic proteolytic processing site that is cleaved to produce a mature protein containing seven conserved cysteine residues. The members of this family are regulators of cell growth and differentiation in both embryonic and adult tissues. Studies in rodents suggest that this protein is involved in the establishment of left-right asymmetry in early embryogenesis and in neural development in later embryogenesis. This protein is transcribed from a bicistronic mRNA that also encodes the longevity assurance gene. | Growth factor | Mesoderm development | TGF-beta signaling pathway->Transforming growth factor beta |
| ***GJB2*** | 2706 | 13q11-q12 | Gap junction protein, beta 2, 26kDa. Gap junctions were first characterized by electron microscopy as regionally specialized structures on plasma membranes of contacting adherent cells. These structures were shown to consist of cell-to-cell channels. Proteins, called connexins, purified from fractions of enriched gap junctions from different tissues differ. The connexins are designated by their molecular mass. Another system of nomenclature divides gap junction proteins into 2 categories, alpha and beta, according to sequence similarities at the nucleotide and amino acid levels. For example, CX43 (MIM 121014) is designated alpha-1 gap junction protein, whereas CX32 (GJB1; MIM 304040) and CX26 are called beta-1 and beta-2 gap junction proteins, respectively. This nomenclature emphasizes that CX32 and CX26 are more homologous to each other than either of them is to CX43. | Gap junction | Signal transduction | N/A |
| ***GLI2*** | 2736 | 2q14.2 | GLI-Kruppel family member GLI2. This gene encodes a protein which belongs to the C2H2-type zinc finger protein subclass of the Gli family. Members of this subclass are characterized as transcription factors which bind DNA through zinc finger motifs. These motifs contain conserved H-C links. Gli family zinc finger proteins are mediators of Sonic hedgehog (Shh) signaling and they are implicated as potent oncogenes in the embryonal carcinoma cell. The protein encoded by this gene localizes to the cytoplasm and activates patched Drosophila homolog (PTCH) gene expression. It is also thought to play a role during embryogenesis. The encoded protein is associated with several phenotypes- Greig cephalopolysyndactyly syndrome, Pallister-Hall syndrome, preaxial polydactyly type IV, postaxial polydactyly types A1 and B. | Zinc finger transcription factor | mRNA transcription regulation; Embryogenesis | Hedgehog signaling pathway->Cubitus interruptus | Hedgehog signaling pathway->Cubitus interruptus repressor |
| ***GLI3*** | 2737 | 7p14.1 | GLI-Kruppel family member GLI3 (Greig cephalopolysyndactyly syndrome). This gene encodes a protein which belongs to the C2H2-type zinc finger proteins subclass of the Gli family. They are characterized as DNA-binding transcription factors and are mediators of Sonic hedgehog (Shh) signaling. The protein encoded by this gene localizes in the cytoplasm and activates patched Drosophila homolog (PTCH) gene expression. It is also thought to play a role during embryogenesis. Mutations in this gene have been associated with several diseases, including Greig cephalopolysyndactyly syndrome, Pallister-Hall syndrome, preaxial polydactyly type IV, and postaxial polydactyly types A1 and B. | Zinc finger transcription factor | mRNA transcription regulation; Neurogenesis; Mesoderm development | Hedgehog signaling pathway->Cubitus interruptus | Hedgehog signaling pathway->Cubitus interruptus repressor |
| ***GNMT*** | 27232 | 6p12 | Glycine N-methyltransferase. Glycine N-methyltransferase (GNMT; EC 2.1.1.20) catalyzes the synthesis of N-methylglycine (sarcosine) from glycine using S-adenosylmethionine (AdoMet) as the methyl donor. The enzyme was first described by Blumenstein and Williams (1960) in guinea pig liver. GNMT acts as an enzyme to regulate the ratio of S-adenosylmethionine to S-adenosylhomocysteine (AdoHcy) and participates in the detoxification pathway in liver cells. | Molecular function unclassified | Biological process unclassified | N/A |
| ***GPC3*** | 2719 | Xq26.1 | Glypican 3. Cell surface heparan sulfate proteoglycans are composed of a membrane-associated protein core substituted with a variable number of heparan sulfate chains. Members of the glypican-related integral membrane proteoglycan family (GRIPS) contain a core protein anchored to the cytoplasmic membrane via a glycosyl phosphatidylinositol linkage. These proteins may play a role in the control of cell division and growth regulation. Deletion mutations in this gene are associated with Simpson-Golabi-Behmel syndrome. | Cell adhesion molecule; Extracellular matrix glycoprotein | Cell adhesion | N/A |
| ***GPR51*** | 9568 | 9q22.1-q22.3 | Same as GABBR2 or gamma-aminobutyric acid (GABA) B receptor, 2. B-type receptors for the neurotransmitter GABA (gamma-aminobutyric acid) inhibit neuronal activity through G protein-coupled second-messenger systems, which regulate the release of neurotransmitters and the activity of ion channels and adenylyl cyclase. See GABBR1 (MIM 603540) for additional background information on GABA-B receptors. | G-protein coupled receptor | G-protein mediated signaling; Nerve-nerve synaptic transmission | GABA-B_receptor_II_signaling->Gamma-aminobutyric acid B receptor |
| ***GRLF1*** | 2909 | 19q13.3 | Glucocorticoid receptor DNA binding factor 1. The human glucocorticoid receptor DNA binding factor, which associates with the promoter region of the glucocorticoid receptor gene (hGR gene), is a repressor of glucocorticoid receptor transcription. The amino acid sequence deduced from the cDNA sequences show the presence of three sequence motifs characteristic of a zinc finger and one motif suggestive of a leucine zipper in which 1 cysteine is found instead of all leucines. The GRLF1 enhances the homologous down-regulation of wild-type hGR gene expression. Biochemical analysis suggests that GRLF1 interaction is sequence specific and that transcriptional efficacy of GRLF1 is regulated through its interaction with specific sequence motif. The level of expression is regulated by glucocorticoids. | Other G-protein modulator | mRNA transcription regulation | N/A |
| ***GSTA4*** | 2941 | 6p12.2 | Glutathione S-transferase A4. Cytosolic and membrane-bound forms of glutathione S-transferase are encoded by two distinct supergene families. These enzymes are involved in cellular defense against toxic, carcinogenic, and pharmacologically active electrophilic compounds. At present, eight distinct classes of the soluble cytoplasmic mammalian glutathione S-transferases have been identified: alpha, kappa, mu, omega, pi, sigma, theta and zeta. This gene encodes a glutathione S-tranferase belonging to the alpha class. The alpha class genes, which are located in a cluster on chromosome 6, are highly related and encode enzymes with glutathione peroxidase activity that function in the detoxification of lipid peroxidation products. Reactive electrophiles produced by oxidative metabolism have been linked to a number of degenerative diseases including Parkinson's disease, Alzheimer's disease, cataract formation, and atherosclerosis. | Other transferase | Detoxification | N/A |
| ***GSTM1*** | 2944 | 1p13.3 | Glutathione S-transferase M1. Cytosolic and membrane-bound forms of glutathione S-transferase are encoded by two distinct supergene families. At present, eight distinct classes of the soluble cytoplasmic mammalian glutathione S-transferases have been identified: alpha, kappa, mu, omega, pi, sigma, theta and zeta. This gene encodes a glutathione S-transferase that belongs to the mu class. The mu class of enzymes functions in the detoxification of electrophilic compounds, including carcinogens, therapeutic drugs, environmental toxins and products of oxidative stress, by conjugation with glutathione. The genes encoding the mu class of enzymes are organized in a gene cluster on chromosome 1p13.3 and are known to be highly polymorphic. These genetic variations can change an individual's susceptibility to carcinogens and toxins as well as affect the toxicity and efficacy of certain drugs. Null mutations of this class mu gene have been linked with an increase in a number of cancers, likely due to an increased susceptibility to environmental toxins and carcinogens. Multiple protein isoforms are encoded by transcript variants of this gene. | Other transferase | Detoxification | N/A |
| ***GSTM3*** | 2947 | 1p13.3 | Glutathione S-transferase M3 (brain). Cytosolic and membrane-bound forms of glutathione S-transferase are encoded by two distinct supergene families. At present, eight distinct classes of the soluble cytoplasmic mammalian glutathione S-transferases have been identified: alpha, kappa, mu, omega, pi, sigma, theta and zeta. This gene encodes a glutathione S-transferase that belongs to the mu class. The mu class of enzymes functions in the detoxification of electrophilic compounds, including carcinogens, therapeutic drugs, environmental toxins and products of oxidative stress, by conjugation with glutathione. The genes encoding the mu class of enzymes are organized in a gene cluster on chromosome 1p13.3 and are known to be highly polymorphic. These genetic variations can change an individual's susceptibility to carcinogens and toxins as well as affect the toxicity and efficacy of certain drugs. Mutations of this class mu gene have been linked with a slight increase in a number of cancers, likely due to exposure with environmental toxins. | Other transferase | Detoxification | N/A |
| ***GSTP1*** | 2950 | 11q13.2 | Glutathione S-transferase pi. Glutathione S-transferases (GSTs) are a family of enzymes that play an important role in detoxification by catalyzing the conjugation of many hydrophobic and electrophilic compounds with reduced glutathione. Based on their biochemical, immunologic, and structural properties, the soluble GSTs are categorized into 4 main classes: alpha, mu, pi, and theta. The glutathione S-transferase pi gene (GSTP1) is a polymorphic gene encoding active, functionally different GSTP1 variant proteins that are thought to function in xenobiotic metabolism and play a role in susceptibility to cancer, and other diseases. | Other transferase | Detoxification | N/A |
| ***GSTT1*** | 2952 | 22q11.23 | Glutathione S-transferase theta 1. Glutathione S-transferase (GST) theta 1 (GSTT1) is a member of a superfamily of proteins that catalyze the conjugation of reduced glutathione to a variety of electrophilic and hydrophobic compounds. Human GSTs can be divided into five main classes: alpha, mu, pi, theta, and zeta. The theta class includes GSTT1 and GSTT2. The GSTT1 and GSTT2 share 55% amino acid sequence identity and both of them were claimed to have an important role in human carcinogenesis. The GSTT1 gene is located approximately 50kb away from the GSTT2 gene. The GSTT1 and GSTT2 genes have a similar structure, being composed of five exons with identical exon/intron boundaries. | Other transferase; Epimerase/racemase | Detoxification; Antioxidation and free radical removal | N/A |
| ***HIC1*** | 3090 | 17p13.3 | Hypermethylated in cancer 1.Data indicate that the intracellular amounts of HIC1 protein can modulate the level of the transcriptional stimulation of the genes regulated by canonical Wnt/beta-catenin signaling. | Zinc finger transcription factor; Other DNA-binding protein | mRNA transcription regulation | N/A |
| ***HIF1A*** | 3091 | 14q23.2 | Hypoxia-inducible factor 1, alpha subunit (basic helix-loop-helix transcription factor). Hypoxia-inducible factor-1 (HIF1) is a transcription factor found in mammalian cells cultured under reduced oxygen tension that plays an essential role in cellular and systemic homeostatic responses to hypoxia. HIF1 is a heterodimer composed of an alpha subunit and a beta subunit. The beta subunit has been identified as the aryl hydrocarbon receptor nuclear translocator (ARNT). This gene encodes the alpha subunit of HIF-1. Overexpression of a natural antisense transcript (aHIF) of this gene has been shown to be associated with nonpapillary renal carcinomas. Two alternative transcripts encoding different isoforms have been identified. | Basic helix-loop-helix transcription factor; Nucleic acid binding | mRNA transcription regulation; Neurogenesis | Hypoxia response via HIF activation->Hypoxia inducible factor-1 alpha subunit; VEGF signaling pathway->Hypoxia-Inducible Factor 1; Angiogenesis->Hypoxia Inducible Factor-1 |
| ***HOGG1*** | 4968 | 3p26.2 | Same as OGG1 or 8-oxoguanine DNA glycosylase. This gene encodes the enzyme responsible for the excision of 8-oxoguanine, a mutagenic base byproduct which occurs as a result of exposure to reactive oxygen. The action of this enzyme includes lyase activity for chain cleavage. Alternative splicing of the C-terminal region of this gene classifies splice variants into two major groups, type 1 and type 2, depending on the last exon of the sequence. Type 1 alternative splice variants end with exon 7 and type 2 end with exon 8. All variants share the N-terminal region in common. Many alternative splice variants for this gene have been described, but the full-length nature for every variant has not been determined. The N-terminus of this gene contains a mitochondrial targetting signal, essential for mitochondrial localization. | DNA glycosylase | DNA repair | N/A |
| ***HOXA7*** | 3204 | 7p15-p14 | Homeobox A7. In vertebrates, the genes encoding the class of transcription factors called homeobox genes are found in clusters named A, B, C, and D on four separate chromosomes. Expression of these proteins is spatially and temporally regulated during embryonic development. This gene is part of the A cluster on chromosome 7 and encodes a DNA-binding transcription factor which may regulate gene expression, morphogenesis, and differentiation. For example, the encoded protein represses the transcription of differentiation-specific genes during keratinocyte proliferation, but this repression is then overcome by differentiation signals. This gene is highly similar to the antennapedia (Antp) gene of Drosophila. | Homeobox transcription factor; Nucleic acid binding | mRNA transcription regulation; Segment specification; Neurogenesis | Wnt signaling pathway->Wnt Target Genes |
| ***HOXB6*** | 3216 | 17q21.3 | Homeobox B6. This gene is a member of the Antp homeobox family and encodes a protein with a homeobox DNA-binding domain. It is included in a cluster of homeobox B genes located on chromosome 17. The encoded protein functions as a sequence-specific transcription factor that is involved in development, including that of lung and skin, and has been localized to both the nucleus and cytoplasm. Altered expression of this gene or a change in the subcellular localization of its protein is associated with some cases of acute myeloid leukemia and colorectal cancer. | Homeobox transcription factor; Nucleic acid binding | mRNA transcription regulation; Segment specification; Neurogenesis | Wnt signaling pathway->Wnt Target Genes |
| ***HSP90*** | 3320 | 14q32.33 | Heat shock protein 90kDa alpha (cytosolic), class A member 1. See Entrez Gene for details. | Hsp 90 family chaperone | Protein folding; Stress response | N/A |
| ***HYAL1*** | 3373 | 3p21.3-p21.2 | Hyaluronoglucosaminidase 1. This gene encodes a lysosomal hyaluronidase. Hyaluronidases intracellularly degrade hyaluronan, one of the major glycosaminoglycans of the extracellular matrix. Hyaluronan is thought to be involved in cell proliferation, migration and differentiation. This enzyme is active at an acidic pH and is the major hyaluronidase in plasma. Mutations in this gene are associated with mucopolysaccharidosis type IX, or hyaluronidase deficiency. The gene is one of several related genes in a region of chromosome 3p21.3 associated with tumor suppression. Eight alternatively spliced transcript variants of this gene encoding six distinct isoforms have been described. | Receptor; Glycosidase | Other polysaccharide metabolism; Cell adhesion | N/A |
| ***IDH1*** | 3417 | 2q33.3 | Isocitrate dehydrogenase 1 (NADP+), soluble. Isocitrate dehydrogenases catalyze the oxidative decarboxylation of isocitrate to 2-oxoglutarate. These enzymes belong to two distinct subclasses, one of which utilizes NAD(+) as the electron acceptor and the other NADP(+). Five isocitrate dehydrogenases have been reported: three NAD(+)-dependent isocitrate dehydrogenases, which localize to the mitochondrial matrix, and two NADP(+)-dependent isocitrate dehydrogenases, one of which is mitochondrial and the other predominantly cytosolic. Each NADP(+)-dependent isozyme is a homodimer. The protein encoded by this gene is the NADP(+)-dependent isocitrate dehydrogenase found in the cytoplasm and peroxisomes. It contains the PTS-1 peroxisomal targeting signal sequence. The presence of this enzyme in peroxisomes suggests roles in the regeneration of NADPH for intraperoxisomal reductions, such as the conversion of 2, 4-dienoyl-CoAs to 3-enoyl-CoAs, as well as in peroxisomal reactions that consume 2-oxoglutarate, namely the alpha-hydroxylation of phytanic acid. The cytoplasmic enzyme serves a significant role in cytoplasmic NADPH production. | Dehydrogenase | Tricarboxylic acid pathway | TCA cycle->Isocitrate Dehydrogenase |
| ***IFNK*** | 56832 | 9 | Interferon, kappa. This gene encodes a member of the type I interferon family. Type I interferons are a group of related glycoproteins that play an important role in host defenses against viral infections. This protein is expressed in keratinocytes and the gene is found on chromosome 9, adjacent to the type I interferon cluster. | Interferon | Cytokine and chemokine mediated signaling pathway; Interferon-mediated immunity | N/A |
| ***IKKA*** | 1147 | 10q24-q25 | Same as CHUK or conserved helix-loop-helix ubiquitous kinase. This gene encodes a member of the serine/threonine protein kinase family. The encoded protein, a component of a cytokine-activated protein complex that is an inhibitor of the essential transcription factor NF-kappa-B complex, phosphorylates sites that trigger the degradation of the inhibitor via the ubiquination pathway, thereby activating the transcription factor. | Non-receptor serine/threonine protein kinase | Protein phosphorylation; NF-kappaB cascade; Cytokine/chemokine mediated immunity | Interleukin signaling pathway->Inhibitor of kappa-B kinase; T cell activation->IKK; Apoptosis signaling pathway->I-kappa B kinase; Toll receptor signaling pathway->IkappaB_alpha Kinase; B cell activation->IKK |
| ***IKKE*** | 9641 | 1q32.1 | Same as IKBKE or inhibitor of kappa light polypeptide gene enhancer in B-cells, kinase epsilon. Too many to list up here; see Entrez Gene. | Non-receptor serine/threonine protein kinase | Protein phosphorylation; NF-kappaB cascade; Cytokine/chemokine mediated immunity | Inflammation mediated by chemokine and cytokine signaling pathway->I-kappa B kinase; Toll receptor signaling pathway->IkappaB_epsilon Kinase |
| ***INHBA*** | 3624 | 7p15-p13 | Inhibin, beta A (activin A, activin AB alpha polypeptide). The inhibin beta A subunit joins the alpha subunit to form a pituitary FSH secretion inhibitor. Inhibin has been shown to regulate gonadal stromal cell proliferation negatively and to have tumor-suppressor activity. In addition, serum levels of inhibin have been shown to reflect the size of granulosa-cell tumors and can therefore be used as a marker for primary as well as recurrent disease. Because expression in gonadal and various extragonadal tissues may vary severalfold in a tissue-specific fashion, it is proposed that inhibin may be both a growth/differentiation factor and a hormone. Furthermore, the beta A subunit forms a homodimer, activin A, and also joins with a beta B subunit to form a heterodimer, activin AB, both of which stimulate FSH secretion. Finally, it has been shown that the beta A subunit mRNA is identical to the erythroid differentiation factor subunit mRNA and that only one gene for this mRNA exists in the human genome. | Growth factor | Other receptor mediated signaling pathway; Developmental processes; Cell proliferation and differentiation | TGF-beta signaling pathway->Transforming growth factor beta |
| ***INHBB*** | 3625 | 2cen-q13 | Inhibin, beta B (activin AB beta polypeptide). The inhibin beta B subunit joins the alpha subunit to form a pituitary FSH secretion inhibitor. Inhibin has been shown to regulate gonadal stromal cell proliferation negatively and to have tumour-suppressor activity. In addition, serum levels of inhibin have been shown to reflect the size of granulosa-cell tumors and can therefore be used as a marker for primary as well as recurrent disease. Because expression in gonadal and various extragonadal tissues may vary severalfold in a tissue-specific fashion, it is proposed that inhibin may be both a growth/differentiation factor and a hormone. Furthermore, the beta B subunit forms a homodimer, activin B, and also joins with the beta A subunit to form a heterodimer, activin AB, both of which stimulate FSH secretion. | Growth factor | Other receptor mediated signaling pathway; Developmental processes; Cell proliferation and differentiation | TGF-beta signaling pathway->Transforming growth factor beta |
| ***IRF6*** | 3664 | 1q32.2 | Interferon regulatory factor 6. This gene encodes a member of the interferon regulatory transcription factor (IRF) family. Family members share a highly-conserved N-terminal helix-turn-helix DNA-binding domain and a less conserved C-terminal protein-binding domain. Mutations in this gene can cause van der Woude syndrome and popliteal pterygium syndrome. This protein is involved in palate formation. | Other transcription factor; Nucleic acid binding | mRNA transcription regulation; Interferon-mediated immunity; Oncogenesis | N/A |
| ***IRF9*** | 10379 | 14q11.2 | Same as ISGF3G or interferon-stimulated transcription factor 3, gamma 48kDa. IRF9 functions to recruit RNA polymerase II to the promoter of interferon-stimulated genes and requires histone deacetylases | Other transcription factor; Nucleic acid binding | mRNA transcription regulation; Interferon-mediated immunity; Oncogenesis | N/A |
| ***ITGB3*** | 3690 | 17q21.32 | Integrin, beta 3 (platelet glycoprotein IIIa, antigen CD61). The ITGB3 protein product is the integrin beta chain beta 3. Integrins are integral cell-surface proteins composed of an alpha chain and a beta chain. A given chain may combine with multiple partners resulting in different integrins. Integrin beta 3 is found along with the alpha IIb chain in platelets. Integrins are known to participate in cell adhesion as well as cell-surface mediated signalling. | Receptor; Cell adhesion molecule; Extracellular matrix glycoprotein | Cell adhesion-mediated signaling; Cell adhesion; Blood clotting; Cell motility | Integrin signalling pathway->Integrin beta; Blood coagulation->Glycoprotein IIIa |
| ***JAG1*** | 182 | 20p12.2 | Jagged 1 (Alagille syndrome). The jagged 1 protein encoded by JAG1 is the human homolog of the Drosophilia jagged protein. Human jagged 1 is the ligand for the receptor notch 1, the latter a human homolog of the Drosophilia jagged receptor notch. Mutations that alter the jagged 1 protein cause Alagille syndrome. Jagged 1 signalling through notch 1 has also been shown to play a role in hematopoiesis. | Receptor; Membrane-bound signaling molecule; Transcription factor; Defense/immunity protein | mRNA transcription regulation; Immunity and defense; Neurogenesis; Cell proliferation and differentiation; Cell motility | Notch signaling pathway->Serrate; Angiogenesis->Delta; Notch signaling pathway->Serrate and Notch extracellular fragment |
| ***JAG2*** | 3714 | 14q32.33 | Notch signaling pathway->Serrate; Angiogenesis->Delta; Notch signaling pathway->Serrate and Notch extracellular fragment | Receptor; Membrane-bound signaling molecule; Transcription factor; Defense/immunity protein | mRNA transcription regulation; Immunity and defens; Neurogenesis; Cell proliferation and differentiation; Cell motility |
| ***KCNJ2*** | 3759 | 17q24.3 | Potassium inwardly-rectifying channel, subfamily J, member 2. Potassium channels are present in most mammalian cells, where they participate in a wide range of physiologic responses. The protein encoded by this gene is an integral membrane protein and inward-rectifier type potassium channel. The encoded protein, which has a greater tendency to allow potassium to flow into a cell rather than out of a cell, probably participates in establishing action potential waveform and excitability of neuronal and muscle tissues. Mutations in this gene have been associated with Andersen syndrome, which is characterized by periodic paralysis, cardiac arrhythmias, and dysmorphic features. | Voltage-gated potassium channel | Cation transport; Action potential propagation; Muscle contraction | N/A |
| ***KLHL4*** | 56062 | Xq21.3 | Kelch-like 4 (Drosophila). This gene encodes a member of the kelch family of proteins, which are characterized by kelch repeat motifs and a POZ/BTB protein-binding domain. It is thought that kelch repeats are actin binding domains. However, the specific function of this protein has not been determined. Alternative splicing of this gene results in two transcript variants encoding different isoforms. | Non-motor actin binding protein | Cell structure | N/A |
| ***KREMEN1*** | 83999 | 22q12.1 | Kringle containing transmembrane protein 1. This gene encodes a high-affinity dickkopf homolog 1 (DKK1) transmembrane receptor that functionally cooperates with DKK1 to block wingless (WNT)/beta-catenin signaling. The encoded protein is a component of a membrane complex that modulates canonical WNT signaling through lipoprotein receptor-related protein 6 (LRP6). It contains extracellular kringle, WSC, and CUB domains. Alternatively spliced transcript variants encoding distinct isoforms have been observed for this gene. | Other receptor | Cell surface receptor mediated signal transduction | Wnt signaling pathway->Kremen |
| ***KRT14*** | 3861 | 17q12-q21 | Keratin 14 (epidermolysis bullosa simplex, Dowling-Meara, Koebner). This gene encodes a member of the keratin family, the most diverse group of intermediate filaments. This gene product, a type I keratin, is usually found as a heterotetramer with two keratin 5 molecules, a type II keratin. Together they form the cytoskeleton of epithelial cells. Mutations in the genes for these keratins are associated with epidermolysis bullosa simplex. At least one pseudogene has been identified at 17p12-p11. | Intermediate filament; Structural protein | Cell structure | N/A |
| ***KRT18*** | 3875 | 12q13 | Keratin 18. KRT18 encodes the type I intermediate filament chain keratin 18. Keratin 18, together with its filament partner keratin 8, are perhaps the most commonly found members of the intermediate filament gene family. They are expressed in single layer epithelial tissues of the body. Mutations in this gene have been linked to cryptogenic cirrhosis. Two transcript variants encoding the same protein have been found for this gene. | Intermediate filament; Structural protein | Cell structure | N/A |
| ***L1CAM*** | 3897 | Xq28 | L1 cell adhesion molecule. The protein encoded by this gene is an axonal glycoprotein belonging to the immunoglobulin supergene family. The ectodomain, consisting of several immunoglobulin-like domains and fibronectin-like repeats (type III), is linked via a single transmembrane sequence to a conserved cytoplasmic domain. This cell adhesion molecule plays an important role in nervous system development, including neuronal migration and differentiation. Mutations in the gene cause three X-linked neurological syndromes known by the acronym CRASH (corpus callosum hypoplasia, retardation, aphasia, spastic paraplegia and hydrocephalus). Alternative splicing of a neuron-specific exon is thought to be functionally relevant. | CAM family adhesion molecule | Cell adhesion-mediated signaling; Cell adhesion; Neurogenesis | N/A |
| ***LCAT*** | 3931 | 16q22.1 | Lecithin-cholesterol acyltransferase. This gene encodes the extracellular cholesterol esterifying enzyme, lecithin-cholesterol acyltransferase. The esterification of cholesterol is required for cholesterol transport. Mutations in this gene have been found to cause fish-eye disease as well as LCAT deficiency. | Acyltransferase | Cholesterol metabolism | N/A |
| ***LDLR*** | 3949 | 19p13.3 | Low density lipoprotein receptor (familial hypercholesterolemia). The low density lipoprotein receptor (LDLR) gene family consists of cell surface proteins involved in receptor-mediated endocytosis of specific ligands. Low density lipoprotein (LDL) is normally bound at the cell membrane and taken into the cell ending up in lysosomes where the protein is degraded and the cholesterol is made available for repression of microsomal enzyme 3-hydroxy-3-methylglutaryl coenzyme A (HMG CoA) reductase, the rate-limiting step in cholesterol synthesis. At the same time, a reciprocal stimulation of cholesterol ester synthesis takes place. Mutations in this gene cause the autosomal dominant disorder, familial hypercholesterolemia. | Other receptor | Oogenesis | Alzheimer disease-presenilin pathway |
| ***LEF1*** | 51176 | 4q25 | Lymphoid enhancer-binding factor 1.Lymphoid enhancer-binding factor-1 (LEF1) is a 48-kD nuclear protein that is expressed in pre-B and T cells. It binds to a functionally important site in the T-cell receptor-alpha (TCRA; MIM 186880) enhancer and confers maximal enhancer activity. LEF1 belongs to a family of regulatory proteins that share homology with high mobility group protein-1 (HMG1; MIM 163905). | Nucleic acid binding | mRNA transcription regulation; Developmental processes | Cadherin signaling pathway->Transcription factor 4/Lymphoid enhancer-binding factor; Alzheimer disease-presenilin pathway->T-cell factor/lymphoid enhancer factor; Wnt signaling pathway->T cell factor |
| ***LEFTY2*** | 7044 | 1q42.1 | Left-right determination factor 2. This gene encodes a member of the TGF-beta family of proteins. The encoded protein is secreted and plays a role in left-right asymmetry determination of organ systems during development. The protein may also play a role in endometrial bleeding. Mutations in this gene have been associated with left-right axis malformations, particularly in the heart and lungs. Some types of infertility have been associated with dysregulated expression of this gene in the endometrium. Alternative processing of this protein can yield three different products. This gene is closely linked to both a related family member and a related pseudogene. | Growth factor | Other receptor mediated signaling pathway; Developmental processes; Cell proliferation and differentiation | TGF-beta signaling pathway->Transforming growth factor beta |
| ***LHX8*** | 431707 | 1p31.1 | LIM homeobox 8. Sohlh1 and Lhx8 are two germ cell-specific, critical regulators of oogenesis. | Homeobox transcription factor; Other zinc finger transcription factor; Nucleic acid binding | mRNA transcription regulation; Neurogenesis | N/A |
| ***LIMK1*** | 3984 | 7q11.23 | LIM domain kinase 1. There are approximately 40 known eukaryotic LIM proteins, so named for the LIM domains they contain. LIM domains are highly conserved cysteine-rich structures containing 2 zinc fingers. Although zinc fingers usually function by binding to DNA or RNA, the LIM motif probably mediates protein-protein interactions. LIM kinase-1 and LIM kinase-2 belong to a small subfamily with a unique combination of 2 N-terminal LIM motifs and a C-terminal protein kinase domain. LIMK1 is likely to be a component of an intracellular signaling pathway and may be involved in brain development. LIMK1 hemizygosity is implicated in the impaired visuospatial constructive cognition of Williams syndrome. | Non-receptor serine/threonine protein kinase | Protein phosphorylation; Other intracellular signaling cascade | Cytoskeletal regulation by Rho GTPase->LIM domain kinase 1 |
| ***LIPC*** | 3990 | 15q21-q23 | Lipase, hepatic. LIPC encodes hepatic triglyceride lipase, which is expressed in liver. LIPC has the dual functions of triglyceride hydrolase and ligand/bridging factor for receptor-mediated lipoprotein uptake. | Lipase | Lipid metabolism | N/A |
| ***LMX1B*** | 4010 | 9q34 | LIM homeobox transcription factor 1, beta. Lmx1b is a homeodomain transcription factor required for glomerular basement membrane collagen expression by podocytes. Its absence in nail-patella syndrome causes abnormalities in many organ systems. | Homeobox transcription factor; Other zinc finger transcription factor; Nucleic acid binding | mRNA transcription regulation; Developmental processes | N/A |
| ***LOR*** | 4014 | 1q21 | Loricrin. This gene encodes loricrin, a major protein component of the cornified cell envelope found in terminally differentiated epidermal cells. Mutations in this gene are associated with Vohwinkel's syndrome and progressive symmetric erythrokeratoderma, both inherited skin diseases. | Molecular function unclassified | Biological process unclassified | N/A |
| ***LPL*** | 4023 | 8p22 | Lipoprotein lipase. LPL encodes lipoprotein lipase, which is expressed in heart, muscle, and adipose tissue. LPL functions as a homodimer, and has the dual functions of triglyceride hydrolase and ligand/bridging factor for receptor-mediated lipoprotein uptake. Severe mutations that cause LPL deficiency result in type I hyperlipoproteinemia, while less extreme mutations in LPL are linked to many disorders of lipoprotein metabolism. | Lipase | Lipid and fatty acid transport; Transport | 2-arachidonoylglycerol biosynthesis->Diacylglycerol lipase |
| ***MARK4*** | 57787 | 19q13.3 | MAP/microtubule affinity-regulating kinase 4. MARK4 is likely to be directly involved in microtubule organization in neuronal cells. | Non-motor microtubule binding protein; Non-receptor serine/threonine protein kinase | Protein phosphorylation; Cell structure | N/A |
| ***MAT1A*** | 4143 | 10q22 | Methionine adenosyltransferase I, alpha. This gene encodes methionine adenosyltransferase I (alpha isoform), which catalyzes the formation of S-adenosylmethionine from methionine and ATP. Methionine adenosyltransferase deficiency is caused by recessive and dominant mutations, the latter identified in autosomal dominant persistant hypermethioninemia. | Nucleotidyltransferase | Other amino acid metabolism | S adenosyl methionine biosynthesis->S-Adenosyl methionine synthetase |
| ***MAT2A*** | 4144 | 2p11.2 | Methionine adenosyltransferase II, alpha. NF-kappa B and AP-1 are required for basal MAT2A expression in HepG2 cells and mediate the increase in MAT2A expression in response to TNF-alpha. beta subunit associates with cirrhosis and cancer providing a proliferative advantage in hepatoma cells through its interaction with methionine adenosyltransferase II alpha2 and down-regulation of S-adenosylmethionine levels. | Nucleotidyltransferase | Other amino acid metabolism | S adenosyl methionine biosynthesis->S-Adenosyl methionine synthetase |
| ***MAT2B*** | 27430 | 5q34-q35.1 | Methionine adenosyltransferase II, beta. The protein encoded by this gene belongs to the methionine adenosyltransferase (MAT) family. MAT catalyzes the biosynthesis of S-adenosylmethionine from methionine and ATP. This protein is the regulatory beta subunit of MAT. Alternative splicing occurs at this locus and two transcript variants encoding distinct isoforms have been identified. | Dehydrogenase; Reductase | Carbohydrate metabolism | O-antigen biosynthesis->dTDP-4-dehydrorhamnose reductase |
| ***MDCR*** | 4186 | 17p13.3 | Miller-Dieker syndrome chromosome region. MDCR is defined as the region of chromosome 17 that, when mutated or lost, results in Miller-Dieker Syndrome (MDS). Mutations causing MDS have been mapped to multiple genes in this region, including ABR, 14-3-3-epsilon, CRK, MYO1C, SKIP, PITPNA, SCARF1, RILP, PRP8, and SERPINF1. MDS is sometimes accompanied by additional mutations in the LIS1/PAFAH1B1 gene, which results in a more severe form of lissencephaly seen only in patients with MDS. | Homeobox transcription factor; Other DNA-binding protein | mRNA transcription regulation; Neurogenesis | N/A |
| ***MDR1*** | 5243 | 7q21.1 | Same as ABCB1 or ATP-binding cassette, sub-family B (MDR/TAP), member 1. The membrane-associated protein encoded by this gene is a member of the superfamily of ATP-binding cassette (ABC) transporters. ABC proteins transport various molecules across extra- and intra-cellular membranes. ABC genes are divided into seven distinct subfamilies (ABC1, MDR/TAP, MRP, ALD, OABP, GCN20, White). This protein is a member of the MDR/TAP subfamily. Members of the MDR/TAP subfamily are involved in multidrug resistance. The protein encoded by this gene is an ATP-dependent drug efflux pump for xenobiotic compounds with broad substrate specificity. It is responsible for decreased drug accumulation in multidrug-resistant cells and often mediates the development of resistance to anticancer drugs. This protein also functions as a transporter in the blood-brain barrier. | ATP-binding cassette (ABC) transporter | Extracellular transport and import | N/A |
| ***MID1*** | 4281 | [Xp22](http://www.ncbi.nlm.nih.gov/mapview/maps.cgi?ORG=hum&chr=X&maps=cntg-r,clone,model,ugHs,loc&VERBOSE=ON&cmd=focus&fill=40&size=40&query=MID1%5BSYM%5D) | Midline 1 (Opitz/BBB syndrome). The protein encoded by this gene is a member of the tripartite motif (TRIM) family, also known as the 'RING-B box-coiled coil' (RBCC) subgroup of RING finger proteins. The TRIM motif includes three zinc-binding domains, a RING, a B-box type 1 and a B-box type 2, and a coiled-coil region. This protein forms homodimers which associate with microtubules in the cytoplasm. The protein is likely involved in the formation of multiprotein structures acting as anchor points to microtubules. Mutations in this gene have been associated with the X-linked form of Opitz syndrome, which is characterized by midline abnormalities such as cleft lip, laryngeal cleft, heart defects, hypospadias, and agenesis of the corpus callosum. This gene was also the first example of a gene subject to X inactivation in human while escaping it in mouse. Several different transcript variants are generated by alternate splicing; however, the full length nature of two variants has not been determined. | Non-motor microtubule binding protein; Ubiquitin-protein ligase | Proteolysis; Developmental processes | N/A |
| ***MMEL2*** | 79258 | 1p36 | Membrane metallo-endopeptidase-like 1. The protein encoded by this gene is a member of the neutral endopeptidase (NEP) or membrane metallo-endopeptidase (MME) family. Family members play important roles in pain perception, arterial pressure regulation, phosphate metabolism and homeostasis. This protein is a type II transmembrane protein and is thought to be expressed as a secreted protein. This gene is expressed mainly in testis with weak expression in the brain, kidney, and heart. | Metalloprotease | Proteolysis; Cell surface receptor mediated signal transduction; Cell communication; Oncogenesis | N/A |
| ***MMP13*** | 4322 | [11q22.3](http://www.ncbi.nlm.nih.gov/mapview/maps.cgi?ORG=hum&chr=11&maps=cntg-r,clone,model,ugHs,loc&VERBOSE=ON&cmd=focus&fill=40&size=40&query=MMP13%5BSYM%5D) | Matrix metallopeptidase 13 (collagenase 3). Proteins of the matrix metalloproteinase (MMP) family are involved in the breakdown of extracellular matrix in normal physiological processes, such as embryonic development, reproduction, and tissue remodeling, as well as in disease processes, such as arthritis and metastasis. Most MMP's are secreted as inactive proproteins which are activated when cleaved by extracellular proteinases. The protein encoded by this gene cleaves type II collagen more efficiently than types I and III. It may be involved in articular cartilage turnover and cartilage pathophysiology associated with osteoarthritis. The gene is part of a cluster of MMP genes which localize to chromosome 11q22.3. | Metalloprotease; Other extracellular matrix | Proteolysis | Plasminogen activating cascade->Matrix metalloprotease 13; Alzheimer disease-presenilin pathway->Matrix metalloprotease; Plasminogen activating cascade->pro-matrix metalloprotease 13 |
| ***MMP14*** | 4323 | [14q11-q12](http://www.ncbi.nlm.nih.gov/mapview/maps.cgi?ORG=hum&chr=14&maps=cntg-r,clone,model,ugHs,loc&VERBOSE=ON&cmd=focus&fill=40&size=40&query=MMP14%5BSYM%5D) | Matrix metallopeptidase 14 (membrane-inserted). Proteins of the matrix metalloproteinase (MMP) family are involved in the breakdown of extracellular matrix in normal physiological processes, such as embryonic development, reproduction, and tissue remodeling, as well as in disease processes, such as arthritis and metastasis. Most MMP's are secreted as inactive proproteins which are activated when cleaved by extracellular proteinases. However, the protein encoded by this gene is a member of the membrane-type MMP (MT-MMP) subfamily; each member of this subfamily contains a potential transmembrane domain suggesting that these proteins are expressed at the cell surface rather than secreted. This protein activates MMP2 protein, and this activity may be involved in tumor invasion. | Metalloprotease; Other extracellular matrix | Proteolysis | Alzheimer disease-presenilin pathway->Matrix metalloprotease; Alzheimer disease-presenilin pathway->Membrane metalloprotease |
| ***MMP2*** | 4313 | [16q13-q21](http://www.ncbi.nlm.nih.gov/mapview/maps.cgi?ORG=hum&chr=16&maps=cntg-r,clone,model,ugHs,loc&VERBOSE=ON&cmd=focus&fill=40&size=40&query=MMP2%5BSYM%5D) | Matrix metallopeptidase 2 (gelatinase A, 72kDa gelatinase, 72kDa type IV collagenase). Proteins of the matrix metalloproteinase (MMP) family are involved in the breakdown of extracellular matrix in normal physiological processes, such as embryonic development, reproduction, and tissue remodeling, as well as in disease processes, such as arthritis and metastasis. Most MMP's are secreted as inactive proproteins which are activated when cleaved by extracellular proteinases. This gene encodes an enzyme which degrades type IV collagen, the major structural component of basement membranes. The enzyme plays a role in endometrial menstrual breakdown, regulation of vascularization and the inflammatory response. Mutations in this gene have been associated with Winchester syndrome and Nodulosis-Arthropathy-Osteolysis (NAO) syndrome. | Metalloprotease; Other extracellular matrix | Proteolysis | Alzheimer disease-presenilin pathway->Matrix metalloprotease |
| ***MSC*** | 9242 | 8q21 | Musculin (activated B-cell factor-1). The protein encoded by this gene is a transcriptional repressor capable of binding an E-box element either as a homodimer or as a heterodimer with E2A in vitro. The encoded protein also forms heterodimers with E2A proteins in vivo. This protein is capable of inhibiting the transactivation capability of E47, an E2A protein, in mammalian cells. This gene is a downstream target of the B-cell receptor signal transduction pathway. | Basic helix-loop-helix transcription factor; Nucleic acid binding | mRNA transcription regulation | N/A |
| ***MSX1*** | 4487 | 4p16.3-p16.1 | msh homeobox 1. This gene encodes a member of the muscle segment homeobox gene family. The encoded protein functions as a transcriptional repressor during embryogenesis through interactions with components of the core transcription complex and other homeoproteins. It may also have roles in limb-pattern formation, craniofacial development, particularly odontogenesis, and tumor growth inhibition. Mutations in this gene, which was once known as homeobox 7, have been associated with nonsyndromic cleft lip with or without cleft palate 5, Witkop syndrome, Wolf-Hirschom syndrome, and autosomoal dominant hypodontia. | Homeobox transcription factor; Other DNA-binding protein | mRNA transcription regulation; Skeletal development | N/A |
| ***MSX2*** | 4488 | 5q34-35 | msh homeobox 2. This gene encodes a member of the muscle segment homeobox gene family. The encoded protein is a transcriptional repressor whose normal activity may establish a balance between survival and apoptosis of neural crest-derived cells required for proper craniofacial morphogenesis. The encoded protein may also have a role in promoting cell growth under certain conditions and may be an important target for the RAS signaling pathways. Mutations in this gene are associated with parietal foramina 1 and craniosynostosis type 2. | Homeobox transcription factor; Other DNA-binding protein | mRNA transcription regulation; Skeletal development | N/A |
| ***MT1A*** | 4489 | 16q13 | Metallothionein 1A. Metallothionein, potential interaction partner for ECRG2, might be involved in regulation of cell proliferation and apoptosis and in various physiological processes. | Molecular function unclassified | Biological process unclassified | N/A |
| ***MT4*** | 84560 | 16q13 | Metallothionein 4 | Molecular function unclassified | Biological process unclassified | N/A |
| ***MTHFD1*** | 4522 | 14q24 | Methylenetetrahydrofolate dehydrogenase (NADP+ dependent) 1, methenyltetrahydrofolate cyclohydrolase, formyltetrahydrofolate synthetase. This gene encodes a protein that possesses three distinct enzymatic activities, 5,10-methylenetetrahydrofolate dehydrogenase, 5,10-methenyltetrahydrofolate cyclohydrolase and 10-formyltetrahydrofolate synthetase. Each of these activities catalyzes one of three sequential reactions in the interconversion of 1-carbon derivatives of tetrahydrofolate, which are substrates for methionine, thymidylate, and de novo purine syntheses. The trifunctional enzymatic activities are conferred by two major domains, an aminoterminal portion containing the dehydrogenase and cyclohydrolase activities and a larger synthetase domain. | Synthase; Other oxidoreductase; Other hydrolase; Other ligase | Amino acid biosynthesis; Other metabolism | Formyltetrahydroformate biosynthesis->Methylenetetrahydrofolate dehydrogenase |
| ***MTHFD2*** | 10797 | 2p13.1 | Methylenetetrahydrofolate dehydrogenase (NADP+ dependent) 2, methenyltetrahydrofolate cyclohydrolase. This gene encodes a nuclear-encoded mitochondrial bifunctional enzyme with methylenetetrahydrofolate dehydrogenase and methenyltetrahydrofolate cyclohydrolase activities. The enzyme functions as a homodimer and is unique in its absolute requirement for magnesium and inorganic phosphate. Formation of the enzyme-magnesium complex allows binding of NAD. Alternative splicing results in multiple transcripts encoding different isoforms. This gene has a pseudogene on chromosome 7. | Molecular function unclassified | Biological process unclassified | N/A |
| ***MTHFR*** | 4524 | 1p36.3 | 5,10-methylenetetrahydrofolate reductase (NADPH). Methylenetetrahydrofolate reductase (EC 1.5.1.20) catalyzes the conversion of 5,10-methylenetetrahydrofolate to 5-methyltetrahydrofolate, a cosubstrate for homocysteine remethylation to methionine. | Reductase | Coenzyme and prosthetic group metabolism | Formyltetrahydroformate biosynthesis->5,10-Methylene tetrahydrofolate reductase |
| ***MTHFS*** | 10588 | 15q25.1 | 5,10-methenyltetrahydrofolate synthetase (5-formyltetrahydrofolate cyclo-ligase). Methenyltetrahydrofolate synthetase regulates folate turnover and accumulation | Other ligase | Other carbon metabolism | N/A |
| ***MTR*** | 4548 | 1q43 | 5-methyltetrahydrofolate-homocysteine methyltransferase. MTR encodes the enzyme 5-methyltetrahydrofolate-homocysteine methyltransferase. This enzyme, also known as cobalamin-dependent methionine synthase, catalyzes the final step in methionine biosynthesis. Mutations in MTR have been identified as the underlying cause of methylcobalamin deficiency complementation group G. | Methyltransferase | Amino acid metabolism | Formyltetrahydroformate biosynthesis->Cobalamin dependent homocysteine transmethylase; S adenosyl methionine biosynthesis->Cobalamin-dependent homocysteine transmethylase; Methionine biosynthesis->Homocysteine transmethylase |
| ***MTRR*** | 4552 | 5p15.3-p15.2 | 5-methyltetrahydrofolate-homocysteine methyltransferase reductase. Methionine is an essential amino acid required for protein synthesis and one carbon metabolism. Its synthesis is catalyzed by the enzyme methionine synthase. Methionine synthase eventually becomes inactive due to the oxidation of its cob(I)alamin cofactor. The protein encoded by this gene regenerates a functional methionine synthase via reductive methylation. It is a member of the ferredoxin-NADP(+) reductase (FNR) family of electron transferases. Patients of the cbl-E complementation group of disorders of folate/cobalamin metabolism are defective in reductive activation of methionine synthase. This gene produces two transcripts. | Reductase | Amino acid biosynthesis; Electron transport | N/A |
| ***MYL2*** | 4633 | 12q23-q24.3 | Myosin, light chain 2, regulatory, cardiac, slow. MYL2 encodes the regulatory light chain associated with cardiac myosin beta (or slow) heavy chain. Ca+ triggers the phosphorylation of regulatory light chain that in turn triggers contraction. Mutations in MYL2 are associated with mid-left ventricular chamber type hypertrophic cardiomyopathy. | Other actin family cytoskeletal protein; Calmodulin related protein | Muscle contraction; Muscle development; Cell structure and motility | N/A |
| ***NAT1*** | 9 | 8p23.1-p21.3 | N-acetyltransferase 1 (arylamine N-acetyltransferase). Too many to enumerate here; see Entrez Gene for details. | Acetyltransferase | Other metabolism | N/A |
| ***NAT2*** | 10 | 8p23.1-21.3 | N-acetyltransferase 2 (arylamine N-acetyltransferase). This gene encodes N-acetyltransferase 2 (arylamine N-acetyltransferase 2). This enzyme functions to both activate and deactivate arylamine and hydrazine drugs and carcinogens. Polymorphisms in this gene are reponsible for the N-acetylation polymorphism in which human populations segregate into rapid,intermediate, and slow acetylator phenotypes. Polymorphisms in NAT2 are also associated with higher incidences of cancer and drug toxicity. A second arylamine N-acetyltransferase gene (NAT1) is located near NAT2. | Acetyltransferase | Other metabolism | N/A |
| ***NBS1*** | 4683 | 8q21 | Same as NBN or Nibrin. Mutations in this gene are associated with Nijmegen breakage syndrome, an autosomal recessive chromosomal instability syndrome characterized by microcephaly, growth retardation, immunodeficiency, and cancer predisposition. The encoded protein is a member of the MRE11/RAD50 double-strand break repair complex which consists of 5 proteins. This gene product is thought to be involved in DNA double-strand break repair and DNA damage-induced checkpoint activation. Two alternatively spliced transcript variants encoding different isoforms have been found for this gene. | Damaged DNA-binding protein | DNA repair; DNA recombination | N/A |
| ***NIPBL*** | 25836 | 5p13.2 | Nipped-B homolog (Drosophila). This gene encodes the homolog of the Drosophila melanogaster Nipped-B gene product and fungal Scc2-type sister chromatid cohesion proteins. The Drosophila protein facilitates enhancer-promoter communication of remote enhancers and plays a role in developmental regulation. It is also homologous to a family of chromosomal adherins with broad roles in sister chromatid cohesion, chromosome condensation, and DNA repair. The human protein has a bipartite nuclear targeting sequence and a putative HEAT repeat. Condensins, cohesins and other complexes with chromosome-related functions also contain HEAT repeats. Mutations in this gene result in Cornelia de Lange syndrome. | Chromatin/chromatin-binding protein | DNA repair; Chromatin packaging and remodeling | N/A |
| ***NNMT*** | 4837 | 11q23.1 | Nicotinamide N-methyltransferase. N-methylation is one method by which drug and other xenobiotic compounds are metabolized by the liver. This gene encodes the protein responsible for this enzymatic activity which uses S-adenosyl methionine as the methyl donor. | Methyltransferase | Other metabolism | N/A |
| ***NOTCH3*** | 4854 | 19p13.2-p13.1 | Notch homolog 3 (Drosophila). This gene encodes the third discovered human homologue of the Drosophilia melanogaster type I membrane protein notch. In Drosophilia, notch interaction with its cell-bound ligands (delta, serrate) establishes an intercellular signalling pathway that plays a key role in neural development. Homologues of the notch-ligands have also been identified in human, but precise interactions between these ligands and the human notch homologues remains to be determined. Mutations in NOTCH3 have been identified as the underlying cause of cerebral autosomal dominant arteriopathy with subcortical infarcts and leukoencephalopathy (CADASIL). | Membrane-bound signaling molecule; Defense/immunity protein | Cell adhesion-mediated signaling; Immunity and defense; Neurogenesis; Cell proliferation and differentiation; Cell motility | Alzheimer disease-presenilin pathway->Notch N-terminal fragment; Alzheimer disease-presenilin pathway->Notch transmembrane fragment; Notch signaling pathway->Notch intracellular soluble fragment; Angiogenesis->Notch Receptor; Alzheimer disease-presenilin pathway->Notch extracellular fragment; Alzheimer disease-presenilin pathway->Notch; Alzheimer disease-presenilin pathway->Notch fragment; Alzheimer disease-presenilin pathway->Notch intracellular domain; Alzheimer disease-presenilin pathway->Notch C-terminal fragment; Notch signaling pathway->Notch intracellular tethered fragment; Notch signaling pathway->Serrate and Notch extracellular fragment; Notch signaling pathway->Notch; Notch signaling pathway->Notch transmembrane domain fragment |
| ***NQO1*** | 1728 | 16q21.1 | NAD(P)H dehydrogenase, quinone 1. This gene is a member of the NAD(P)H dehydrogenase (quinone) family and encodes a cytoplasmic 2-electron reductase. This FAD-binding protein forms homodimers and reduces quinones to hydroquinones. This protein's enzymatic activity prevents the one electron reduction of quinones that results in the production of radical species. Mutations in this gene have been associated with tardive dyskinesia (TD), an increased risk of hematotoxicity after exposure to benzene, and susceptibility to various forms of cancer. Altered expression of this protein has been seen in many tumors and is also associated with Alzheimer's disease (AD). Alternate transcriptional splice variants, encoding different isoforms, have been characterized. | Molecular function unclassified | Biological process unclassified | N/A |
| ***NR3C1*** | 2908 | 5q31 | Nuclear receptor subfamily 3, group C, member 1 (glucocorticoid receptor). The protein encoded by this gene is a receptor for glucocorticoids that can act as both a transcription factor and as a regulator of other transcription factors. This protein can also be found in heteromeric cytoplasmic complexes along with heat shock factors and immunophilins. The protein is typically found in the cytoplasm until it binds a ligand, which induces transport into the nucleus. Mutations in this gene are a cause of glucocorticoid resistance, or cortisol, resistance. Alternate splicing, the use of at least three different promoters, and alternate translation initiation sites result in several transcript variants encoding the same protein or different isoforms, but the full-length nature of some variants has not been determined. | Nuclear hormone receptor; Transcription factor; Nucleic acid binding | Biological process unclassified | N/A |
| ***NRXN2*** | 9379 | 11q13 | Neurexin 2. Neurexins are a family of proteins that function in the vertebrate nervous system as cell adhesion molecules and receptors. They are encoded by several unlinked genes of which two, NRXN1 and NRXN3, are among the largest known human genes. Three of the genes (NRXN1-3) utilize two alternate promoters and include numerous alternatively spliced exons to generate thousands of distinct mRNA transcripts and protein isoforms. The majority of transcripts are produced from the upstream promoter and encode alpha-neurexin isoforms; a much smaller number of transcripts are produced from the downstream promoter and encode beta-neurexin isoforms. The alpha-neurexins contain epidermal growth factor-like (EGF-like) sequences and laminin G domains, and have been shown to interact with neurexophilins. The beta-neurexins lack EGF-like sequences and contain fewer laminin G domains than alpha-neurexins. | Other receptor | Cell adhesion-mediated signaling; Cell adhesion; Synaptic transmission | N/A |
| ***NSDHL*** | 50814 | Xq28 | NAD(P) dependent steroid dehydrogenase-like. NSDHL, an enzyme involved in cholesterol synthesis, traffics through the Golgi and accumulates on ER membranes and on the surface of lipid droplets. | Dehydratase; Epimerase/racemase | Cholesterol metabolism | Androgen/estrogene/progesterone biosynthesis->3-beta-hydroxy-delta(5)-steroid dehydrogenase; Androgen/estrogene/progesterone biosynthesis->3beta-Hydroxysteroid dehydrogenase |
| ***OSR2*** | 116039 | 8q22.2 | Odd-skipped related 2 (Drosophila) | Other zinc finger transcription factor; Nucleic acid binding | mRNA transcription; Other developmental process; Segment specification | N/A |
| ***PAX3*** | 5077 | 2q35-q37; 2q35 | Paired box gene 3 (Waardenburg syndrome 1). This gene is a member of the paired box (PAX) family of transcription factors. Members of the PAX family typically contain a paired box domain and a paired-type homeodomain. These genes play critical roles during fetal development. Mutations in paired box gene 3 are associated with Waardenburg syndrome, craniofacial-deafness-hand syndrome, and alveolar rhabdomyosarcoma. The translocation t(2;13)(q35;q14), which represents a fusion between PAX3 and the forkhead gene, is a frequent finding in alveolar rhabdomyosarcoma. Alternative splicing results in transcripts encoding isoforms with different C-termini. | Homeobox transcription factor; Other DNA-binding protein | mRNA transcription regulation; Segment specification; Neurogenesis | N/A |
| ***PAX8*** | 7849 | 2q12-q14 | Paired box gene 8. This gene is a member of the paired box (PAX) family of transcription factors. Members of this gene family typically encode proteins which contain a paired box domain, an octapeptide, and a paired-type homeodomain. This nuclear protein is involved in thyroid follicular cell development and expression of thyroid-specific genes. Mutations in this gene have been associated with thyroid dysgenesis, thyroid follicular carcinomas and atypical follicular thyroid adenomas. Alternate transcriptional splice variants, encoding different isoforms, have been characterized. | Homeobox transcription factor; Other DNA-binding protein | mRNA transcription regulation; Neurogenesis; Gut mesoderm development | N/A |
| ***PAX9*** | 5083 | 14q12-q13 | Paired box gene 9. This gene is a member of the paired box (PAX) family of transcription factors. Members of this gene family typically contain a paired box domain, an octapeptide, and a paired-type homeodomain. These genes play critical roles during fetal development and cancer growth. The specific function of the paired box gene 9 is unknown but it may involve development of stratified squamous epithelia as well as various organs and skeletal elements. | Homeobox transcription factor; Other DNA-binding protein | mRNA transcription regulation; Skeletal development | N/A |
| ***PDGFC*** | 56034 | 4q32 | Platelet derived growth factor C. The protein encoded by this gene is a member of the platelet-derived growth factor family. The four members of this family are mitogenic factors for cells of mesenchymal origin and are characterized by a core motif of eight cysteines. This gene product appears to form only homodimers. It differs from the platelet-derived growth factor alpha and beta polypeptides in having an unusual N-terminal domain, the CUB domain. | Growth factor | Ligand-mediated signaling; Other developmental process; Cell proliferation and differentiation | Angiogenesis->Platelet-Derived Growth Factor |
| ***PDGFRA*** | 5156 | 4q12 | Platelet-derived growth factor receptor, alpha polypeptide. This gene encodes a cell surface tyrosine kinase receptor for members of the platelet-derived growth factor family. These growth factors are mitogens for cells of mesenchymal origin. The identity of the growth factor bound to a receptor monomer determines whether the functional receptor is a homodimer or a heterodimer, composed of both platelet-derived growth factor receptor alpha and beta polypeptides. Studies in knockout mice, where homozygosity is lethal, indicate that the alpha form of the platelet-derived growth factor receptor is particularly important for kidney development since mice heterozygous for the receptor exhibit defective kidney phenotypes. | Tyrosine protein kinase receptor; Protein kinase | Protein phosphorylation; Receptor protein tyrosine kinase signaling pathway; Stress response; Gametogenesis; Embryogenesis; Cell proliferation and differentiation; Oncogenesis; Cell motility | Angiogenesis->Platelet-Derived Growth Factor Receptor |
| ***PEX7*** | 5191 | 6q21-q22.2 | Peroxisomal biogenesis factor 7. This gene codes for the peroxin 7 receptor protein required for peroxisomal import of proteins containing a peroxisomal targeting signal type 2. Mutations may result in a broad clinical spectrum of Refsum disease. | Other receptor | Peroxisome transport; Transport | N/A |
| ***PHF8*** | 23133 | Xp11.22 | PHD finger protein 8. An important function of PHF8 is in midline formation and in the development of cognitive abilities, and may have a role in X linked mental retardation associated with cleft lip/palate | Other receptor | Peroxisome transport; Transport | N/A |
| ***PIGA*** | 5277 | Xp22.1 | Phosphatidylinositol glycan anchor biosynthesis, class A (paroxysmal nocturnal hemoglobinuria). This gene encodes a protein required for synthesis of N-acetylglucosaminyl phosphatidylinositol (GlcNAc-PI), the first intermediate in the biosynthetic pathway of GPI anchor. The GPI anchor is a glycolipid found on many blood cells and which serves to anchor proteins to the cell surface. Paroxysmal nocturnal hemoglobinuria, an acquired hematologic disorder, has been shown to result from mutations in this gene. Alternate splice variants have been characterized. | Glycosyltransferase | Other polysaccharide metabolism | N/A |
| ***PIPOX*** | 51268 | 17q11.2 | Pipecolic acid oxidase | Oxidase | Other metabolism | N/A |
| ***PITX1*** | 5307 | 5q31 | Paired-like homeodomain transcription factor 1. This gene encodes a member of the RIEG/PITX homeobox family, which is in the bicoid class of homeodomain proteins. Members of this family are involved in organ development and left-right asymmetry. This protein acts as a transcriptional regulator involved in basal and hormone-regulated activity of prolactin. | Homeobox transcription factor; Other DNA-binding protein | mRNA transcription regulation | N/A |
| ***PITX2*** | 5308 | 4q25 | Paired-like homeodomain transcription factor 2. This gene encodes a member of the RIEG/PITX homeobox family, which is in the bicoid class of homeodomain proteins. This protein acts as a transcription factor and regulates procollagen lysyl hydroxylase gene expression. Mutations in this gene are associated with Axenfeld-Rieger syndrome (ARS), iridogoniodysgenesis syndrome (IGDS), and sporadic cases of Peters anomaly. This protein plays a role in the terminal differentiation of somatotroph and lactotroph cell phenotypes. This protein is involved in the development of the eye, tooth and abdominal organs. This protein acts as a transcriptional regulator involved in basal and hormone-regulated activity of prolactin. A similar protein in other vertebrates is involved in the determination of left-right asymmetry during development. Three transcript variants encoding distinct isoforms have been identified for this gene. | Homeobox transcription factor; Other DNA-binding protein | mRNA transcription regulation | N/A |
| ***PKP1*** | 5317 | 1q32 | Plakophilin 1 (ectodermal dysplasia/skin fragility syndrome). This gene encodes a member of the arm-repeat (armadillo) and plakophilin gene families. Plakophilin proteins contain numerous armadillo repeats, localize to cell desmosomes and nuclei, and participate in linking cadherins to intermediate filaments in the cytoskeleton. This protein may be involved in molecular recruitment and stabilization during desmosome formation. Mutations in this gene have been associated with the ectodermal dysplasia/skin fragility syndrome. | Intermediate filament binding protein; Other cell junction protein | Cell communication; Cell adhesion | N/A |
| ***POMT1*** | 10585 | 9q34.1 | Protein-O-mannosyltransferase 1. O-mannosylation is an important protein modification in eukaryotes that is initiated by an evolutionarily conserved family of protein O-mannosyltransferases. POMT1 shares sequence similarity with protein O-mannosyltransferases of S. cerevisiae. In yeast, these enzymes are located in the endoplasmic reticulum (ER) and are required for cell integrity and cell wall rigidity. POMT1 also shows similarity to the Drosophila 'rotated abdomen' (rt) gene, which when mutated causes defects in myogenesis and muscle structure. | Glycosyltransferase | Protein glycosylation | N/A |
| ***PON1*** | 5444 | 7q21.3 | Paraoxonase 1. Too many to enumerate here. See Entrez Gene. | Peroxidase; Esterase | Phosphate metabolism; Detoxification | N/A |
| ***PQBP1*** | 10084 | Xq13, Xp11.23 | Polyglutamine binding protein 1. PQBP1 is a nuclear polyglutamine-binding protein that contains a WW domain. | Transcription cofactor; Select regulatory molecule | mRNA transcription regulation | N/A |
| ***PRDM16*** | 63976 | 1p36.23-p3 | PR domain containing 16. The reciprocal translocation t(1;3)(p36;q21) occurs in a subset of myelodysplastic syndrome (MDS) and acute myeloid leukemia (AML). This gene is located near the 1p36.3 breakpoint and has been shown to be specifically expressed in the t(1:3)(p36,q21)-positive MDS/AML. The protein encoded by this gene is a zinc finger transcription factor and contains an N-terminal PR domain. The translocation results in the overexpression of a truncated version of this protein that lacks the PR domain, which may play an important role in the pathogenesis of MDS and AML. Alternatively spliced transcript variants encoding distinct isoforms have been reported. | KRAB box transcription factor | Biological process unclassified | N/A |
| ***PRRX1*** | 5396 | 1q24.2 | Paired related homeobox 1. The DNA-associated protein encoded by this gene is a member of the paired family of homeobox proteins localized to the nucleus. The protein functions as a transcription co-activator, enhancing the DNA-binding activity of serum response factor, a protein required for the induction of genes by growth and differentiation factors. The protein regulates muscle creatine kinase, indicating a role in the establishment of diverse mesodermal muscle types. Alternative splicing yields two isoforms that differ in abundance and expression patterns. | Homeobox transcription factor; Other DNA-binding protein | mRNA transcription regulation; Neurogenesis | N/A |
| ***PRRX2*** | 51450 | 9q34.1 | Paired related homeobox 2. The DNA-associated protein encoded by this gene is a member of the paired family of homeobox proteins. Expression is localized to proliferating fetal fibroblasts and the developing dermal layer, with downregulated expression in adult skin. Increases in expression of this gene during fetal but not adult wound healing suggest a possible role in mechanisms that control mammalian dermal regeneration and prevent formation of scar response to wounding. The expression patterns provide evidence consistent with a role in fetal skin development and a possible role in cellular proliferation. | Homeobox transcription factor; Other DNA-binding protein | mRNA transcription regulation | N/A |
| ***PTCH1*** | 5727 | 9q22.3 | Patched homolog 1 (Drosophila). This gene encodes a member of the patched gene family. The encoded protein is the receptor for sonic hedgehog, a secreted molecule implicated in the formation of embryonic structures and in tumorigenesis. This gene functions as a tumor suppressor. Mutations of this gene have been associated with nevoid basal cell carcinoma syndrome, esophageal squamous cell carcinoma, trichoepitheliomas, transitional cell carcinomas of the bladder, as well as holoprosencephaly. Alternative spliced variants have been described, but their full length sequences have not be determined. | Molecular function unclassified | Biological process unclassified | Hedgehog signaling pathway->Patched |
| ***PTCH2*** | 8643 | 1p33-p34 | Patched homolog 2 (Drosophila). This gene encodes a member of the patched gene family. The patched protein is the receptor for sonic hedgehog, a secreted molecule implicated in the formation of embryonic structures and in tumorigenesis. This gene is shown to be mutated in a medulloblastoma and in a basal cell carcinoma, suggesting that it plays a role in the development of some tumors. Alternative transcript variants have been described, but their biological function has not been determined. | Molecular function unclassified | Biological process unclassified | Hedgehog signaling pathway->Patched |
| ***PTEN*** | 5728 | 10q23.3 | Phosphatase and tensin homolog (mutated in multiple advanced cancers 1). This gene was identified as a tumor suppressor that is mutated in a large number of cancers at high frequency. The protein encoded this gene is a phosphatidylinositol-3,4,5-trisphosphate 3-phosphatase. It contains a tension like domain as well as a catalytic domain similar to that of the dual specificity protein tyrosine phosphatases. Unlike most of the protein tyrosine phosphatases, this protein preferentially dephosphorylates phosphoinositide substrates. It negatively regulates intracellular levels of phosphatidylinositol-3,4,5-trisphosphate in cells and functions as a tumor suppressor by negatively regulating AKT/PKB signaling pathway. | Protein phosphatase; Other phosphatase | Phospholipid metabolism; Protein phosphorylation; Signal transduction; Cell adhesion; Immunity and defense; Induction of apoptosis; Cell cycle control; Cell proliferation and differentiation; Tumor suppressor | Insulin/IGF pathway-protein kinase B signaling cascade->Phosphatase and tensin homolog; Hypoxia response via HIF activation->phospatase and tensin homologue; PI3 kinase pathway->PTEN; p53 pathway->PTEN; Insulin/IGF pathway-protein kinase B signaling cascade->PTEN; Inflammation mediated by chemokine and cytokine signaling pathway->Phosphatase and tensin homologue; p53 pathway->Phosphatase and tensin homolog deleted on chromosome 10; p53 pathway feedback loops 2->PTEN; p53 pathway feedback loops 2->PTEN |
| ***PTPN11*** | 5781 | 12q24 | Protein tyrosine phosphatase, non-receptor type 11 (Noonan syndrome 1). The protein encoded by this gene is a member of the protein tyrosine phosphatase (PTP) family. PTPs are known to be signaling molecules that regulate a variety of cellular processes including cell growth, differentiation, mitotic cycle, and oncogenic transformation. This PTP contains two tandem Src homology-2 domains, which function as phospho-tyrosine binding domains and mediate the interaction of this PTP with its substrates. This PTP is widely expressed in most tissues and plays a regulatory role in various cell signaling events that are important for a diversity of cell functions, such as mitogenic activation, metabolic control, transcription regulation, and cell migration. Mutations in this gene are a cause of Noonan syndrome as well as acute myeloid leukemia. | Protein phosphatase | Protein phosphorylation; Cytokine and chemokine mediated signaling pathway; Receptor protein tyrosine kinase signaling pathwa; JAK-STAT cascade; Cell adhesion-mediated signaling; Protein targeting; Developmental processes; Cell proliferation and differentiation; Cell structure and motility | Angiogenesis->Tyrosine Phosphatase Shp2; FGF signaling pathway->Src homology 2 domain-containing tyrosine phosphatase 2; Interferon-gamma signaling pathway->protein tyrosine phosphatase |
| ***PVR*** | 5817 | 19q13.2 | Poliovirus receptor | Other receptor; Other defense and immunity protein | Cell adhesion-mediated signaling; Immunity and defense | N/A |
| ***PVRL1*** | 5818 | 11q23 | Poliovirus receptor-related 1 (herpesvirus entry mediator C; nectin). Trans-interacting nectin inhibits non-trans-interacting E-cadherin endocytosis through afadin, Rap1, and p120ctn and further accumulates non-trans-interacting E-cadherin to the nectin-based cell-cell adhesion sites for the formation of adherens junctions. The role of PVRL1 in the sporadic forms of orofacial clefting in multiple populations was studied. | Other receptor; Other defense and immunity protein | Cell adhesion-mediated signaling; Immunity and defense | Alzheimer disease-presenilin pathway |
| ***PVRL2*** | 5819 | 19q13.2-q13.4 | Poliovirus receptor-related 2 (herpesvirus entry mediator B). This gene encodes a single-pass type I membrane glycoprotein with two Ig-like C2-type domains and an Ig-like V-type domain. This protein is one of the plasma membrane components of adherens junctions. It also serves as an entry for certain mutant strains of herpes simplex virus and pseudorabies virus, and it is involved in cell to cell spreading of these viruses. Variations in this gene have been associated with differences in the severity of multiple sclerosis. Alternate transcriptional splice variants, encoding different isoforms, have been characterized. | Other receptor; Other defense and immunity protein | Cell adhesion-mediated signaling; Immunity and defense | N/A |
| ***PVRL3*** | 25945 | 3q13.13 | Poliovirus receptor-related 3. Nectins (e.g., PVRL1; MIM 600644) are immunoglobulin-like adhesion molecules that interact with afadin (AF6; MIM 159559). Afadin is an actin filament-binding protein that connects nectins to the actin cytoskeleton. The nectin-afadin system organizes adherens junctions cooperatively with the cadherin (see MIM 192090)-catenin (see MIM 116805) system in epithelial cells. | Other receptor; Other defense and immunity protein | Cell adhesion-mediated signaling; Immunity and defense | N/A |
| ***RAI1*** | 10743 | 17p11.2 | Retinoic acid induced 1. This gene is located within the Smith-Magenis syndrome region on chromosome 17. It is highly similar to its mouse counterpart and is expressed at high levels mainly in neuronal tissues. The protein encoded by this gene includes a polymorphic polyglutamine tract in the N-terminal domain. Expression of the mouse counterpart in neurons is induced by retinoic acid. This gene is associated with both the severity of the phenotype and the response to medication in schizophrenic patients. | Transcription factor | mRNA transcription | N/A |
| ***RARA*** | 5914 | 17q21 | Retinoic acid receptor, alpha. Too many to enumerate here. See Entrez Gene. | Nuclear hormone receptor; Transcription factor; Nucleic acid binding | mRNA transcription regulation; Cell communication; Developmental processes; Oncogenesis | Vitamin D metabolism and pathway->Retinoid X Receptor, Alpha |
| ***RARB*** | 5915 | [3p24](http://www.ncbi.nlm.nih.gov/mapview/maps.cgi?ORG=hum&chr=3&maps=cntg-r,clone,model,ugHs,loc&VERBOSE=ON&cmd=focus&fill=40&size=40&query=RARB%5BSYM%5D) | Retinoic acid receptor, beta. This gene encodes retinoic acid receptor beta, a member of the thyroid-steroid hormone receptor superfamily of nuclear transcriptional regulators. This receptor localizes to the cytoplasm and to subnuclear compartments. It binds retinoic acid, the biologically active form of vitamin A which mediates cellular signalling in embryonic morphogenesis, cell growth and differentiation. It is thought that this protein limits growth of many cell types by regulating gene expression. The gene was first identified in a hepatocellular carcinoma where it flanks a hepatitis B virus integration site. The gene expresses at least two transcript variants; one additional transcript has been described, but its full length nature has not been determined. | Nuclear hormone receptor; Transcription factor; Nucleic acid binding | mRNA transcription regulation; Cell communication; Developmental processes; Oncogenesis | Vitamin D metabolism and pathway->Retinoid X Receptor, Alpha |
| ***RARG*** | 5916 | [12q13](http://www.ncbi.nlm.nih.gov/mapview/maps.cgi?ORG=hum&chr=12&maps=loc-r,morbid,gene&VERBOSE=ON&ZOOM=3&query=RARG%5BSYM%5D) | Retinoic acid receptor, gamma. Retinoid signaling is attenuated by retinoic acid-induced proteasome-mediated degradation of RARG in human keratocytes. Retinoids cause apoptosis in pancreatic cancer cells via activation of RAR-gamma and altered expression of Bcl-2/Bax. Depletion of vitamin A and retinoid receptors by UV irradiation, together with unchanged or even increased c-Jun levels, might seriously interfere with retinoid signaling and thus promote future tumor development, especially in keratinocytes. | Nuclear hormone receptor; Transcription factor; Nucleic acid binding | mRNA transcription regulation; Cell communication; Developmental processes; Oncogenesis | Vitamin D metabolism and pathway->Retinoid X Receptor, Alpha |
| ***RECQL4*** | 9401 | 8q24.3 | RecQ protein-like 4. Findings suggest a role for RECQL4 in the repair of DNA double-strand breaks by homologous recombination and shed new light onto RECQL4's function in human cells. | DNA helicase | DNA repair | N/A |
| ***RET*** | 5979 | 10q11.2 | Ret proto-oncogene. This gene, a member of the cadherin superfamily, encodes one of the receptor tyrosine kinases, which are cell-surface molecules that transduce signals for cell growth and differentiation. This gene plays a crucial role in neural crest development, and it can undergo oncogenic activation in vivo and in vitro by cytogenetic rearrangement. Mutations in this gene are associated with the disorders multiple endocrine neoplasia, type IIA, multiple endocrine neoplasia, type IIB, Hirschsprung disease, and medullary thyroid carcinoma. Two transcript variants encoding different isoforms have been found for this gene. Additional transcript variants have been described but their biological validity has not been confirmed. | Tyrosine protein kinase receptor; Protein kinase | Protein phosphorylation; Receptor protein tyrosine kinase signaling pathway; Developmental processes; Cell cycle control; Cell proliferation and differentiation; Oncogene | Heterotrimeric G-protein signaling pathway-Gi alpha and Gs alpha mediated pathway->Protein kinase A, regulatory subunit; Endothelin signaling pathway->adenosine-3'-5'-cyclic monophosphate -dependent kinase; Metabotropic glutamate receptor group I pathway->Protein Kinase A |
| ***RFC1*** | 5981 | 4p14-p13 | Replication factor C (activator 1) 1, 145kDa. The protein encoded by this gene is the large subunit of replication factor C, which is a five subunit DNA polymerase accessory protein. Replication factor C is a DNA-dependent ATPase that is required for eukaryotic DNA replication and repair. The protein acts as an activator of DNA polymerases, binds to the 3' end of primers, and promotes coordinated synthesis of both strands. It also may have a role in telomere stability. | Double-stranded DNA binding protein | DNA replication | N/A |
| ***RIP3*** | 11035 | 14q11.2 | Same as RIPK3 or receptor-interacting serine-threonine kinase 3. The same as RIPK3 or receptor-interacting serine-threonine kinase 3. The product of this gene is a member of the receptor-interacting protein (RIP) family of serine/threonine protein kinases, and contains a C-terminal domain unique from other RIP family members. The encoded protein is predominantly localized to the cytoplasm, and can undergo nucleocytoplasmic shuttling dependent on novel nuclear localization and export signals. It is a component of the tumor necrosis factor (TNF) receptor-I signaling complex, and can induce apoptosis and weakly activate the NF-kappaB transcription factor. | Non-receptor serine/threonine protein kinase | Protein phosphorylation; NF-kappaB cascade; Induction of apoptosis | Apoptosis signaling pathway->Receptor interacting protein |
| ***ROR1*** | 4919 | 1p32-p31 | Receptor tyrosine kinase-like orphan receptor 1. The protein encoded by this gene is a receptor protein tyrosine kinase whose cellular role has not been determined. It is a type I membrane protein and belongs to the ROR subfamily of cell surface receptors. Studies of a similar protein in mouse suggest that this protein may interact with another receptor protein tyrosine kinase and may be involved in skeletal and cardiac development. | Tyrosine protein kinase receptor; Protein kinase | Protein phosphorylation; Receptor protein tyrosine kinase signaling pathway | N/A |
| ***ROR2*** | 4920 | 9q22.31 | Receptor tyrosine kinase-like orphan receptor 2. The protein encoded by this gene is a receptor protein tyrosine kinase and type I transmembrane protein that belongs to the ROR subfamily of cell surface receptors. The protein may be involved in the early formation of the chondrocytes and may be required for cartilage and growth plate development. Mutations in this gene can cause brachydactyly type B, a skeletal disorder characterized by hypoplasia/aplasia of distal phalanges and nails. In addition, mutations in this gene can cause the autosomal recessive form of Robinow syndrome, which is characterized by skeletal dysplasia with generalized limb bone shortening, segmental defects of the spine, brachydactyly, and a dysmorphic facial appearance. | Tyrosine protein kinase receptor; Protein kinase | Protein phosphorylation; Receptor protein tyrosine kinase signaling pathway | N/A |
| ***RPS19*** | 6223 | 19q13.2 | Ribosomal protein S19. Ribosomes, the organelles that catalyze protein synthesis, consist of a small 40S subunit and a large 60S subunit. Together these subunits are composed of 4 RNA species and approximately 80 structurally distinct proteins. This gene encodes a ribosomal protein that is a component of the 40S subunit. The protein belongs to the S19E family of ribosomal proteins. It is located in the cytoplasm. Mutations in this gene cause Diamond-Blackfan anemia (DBA), a constitutional erythroblastopenia characterized by absent or decreased erythroid precursors, in a subset of patients. This suggests a possible extra-ribosomal function for this gene in erythropoietic differentiation and proliferation, in addition to its ribosomal function. Higher expression levels of this gene in some primary colon carcinomas compared to matched normal colon tissues has been observed. As is typical for genes encoding ribosomal proteins, there are multiple processed pseudogenes of this gene dispersed through the genome. | Ribosomal protein | Protein biosynthesis | N/A |
| ***RUNX2*** | 860 | 6p21 | Runt-related transcription factor 2. This gene is a member of the RUNX family of transcription factors and encodes a nuclear protein with an Runt DNA-binding domain. This protein is essential for osteoblastic differentiation and skeletal morphogenesis and acts as a scaffold for nucleic acids and regulatory factors involved in skeletal gene expression. The protein can bind DNA both as a monomer or, with more affinity, as a subunit of a heterodimeric complex. Mutations in this gene have been associated with the bone development disorder cleidocranial dysplasia (CCD). Transcript variants that encode different protein isoforms result from the use of alternate promoters as well as alternate splicing. | Other transcription factor | Skeletal development; Oncogenesis | N/A |
| ***RXRG*** | 6258 | 1q22-q23 | Retinoid X receptor, gamma. This gene encodes a member of the retinoid X receptor (RXR) family of nuclear receptors which are involved in mediating the antiproliferative effects of retinoic acid (RA). This receptor forms dimers with the retinoic acid, thyroid hormone, and vitamin D receptors, increasing both DNA binding and transcriptional function on their respective response elements. This gene is expressed at significantly lower levels in non-small cell lung cancer cells. Alternate transcriptional splice variants, encoding different isoforms, have been characterized. | Nuclear hormone receptor; Transcription factor; Nucleic acid binding | Regulation of lipid, fatty acid and steroid metabolism; mRNA transcription regulation; Vitamin metabolism; Signal transduction | N/A |
| ***RYK*** | 6259 | 3q22 | RYK receptor-like tyrosine kinase. The protein encoded by this gene is an atypical member of the family of growth factor receptor protein tyrosine kinases, differing from other members at a number of conserved residues in the activation and nucleotide binding domains. This gene product belongs to a subfamily whose members do not appear to be regulated by phosphorylation in the activation segment. It has been suggested that mediation of biological activity by recruitment of a signaling-competent auxiliary protein may occur through an as yet uncharacterized mechanism. Two alternative splice variants have been identified, encoding distinct isoforms. | Tyrosine protein kinase receptor; Protein kinase | Protein phosphorylation; Receptor protein tyrosine kinase signaling pathway; Neurogenesis | N/A |
| ***SALL1*** | 6299 | 16q12.1 | sal-like 1 (Drosophila). sall1 enhances the canonical Wnt signaling by localizing to heterochromatin | Zinc finger transcription factor; Nucleic acid binding | mRNA transcription regulation; Developmental processes | N/A |
| ***SALL2*** | 6297 | 14q11.1-q12 | sal-like 2 (Drosophila). Epidermal growth factor receptor (a potential therapeutic target) and SALL2 stained most cases of synovial sarcoma; staining was significantly less common among other tested sarcomas. Results suggest that p150(Sal2), acting in part as a p53-independent regulator of p21 and BAX, can function in some cell types as a regulator of cell growth and survival [p150(Sal2)] | Zinc finger transcription factor; Nucleic acid binding | mRNA transcription regulation; Developmental processes | N/A |
| ***SALL3*** | 27164 | 18q23 | sal-like 3 (Drosophila) | Zinc finger transcription factor; Nucleic acid binding | mRNA transcription regulation; Developmental processes | N/A |
| ***SALL4*** | 57167 | 20q13.13-q13.2 | sal-like 4 (Drosophila). Okihiro syndrome is caused by SALL4 mutations. The first identified Duane syndrome gene and the second malformation syndrome resulting from mutations in SAL genes. | Zinc finger transcription factor; Nucleic acid binding | mRNA transcription regulation; Developmental processes | N/A |
| ***SARA1*** | 56681 | 10q22.1 | Same as SAR1A or SAR1 gene homolog A (S. cerevisiae). A protein called SARA modulates cellular trafficking and transports cytoplasmic Smad2/3 to the TGFB receptors to form complexes. | Small GTPase | General vesicle transport | N/A |
| ***SATB2*** | 23314 | 2q33.1 | SATB homeobox 2. SATB2 is identified as the cleft palate gene on chromosome pair 2 which undergoes translocation. | Homeobox transcription factor; Other transcription factor; Nucleic acid binding | mRNA transcription regulation | N/A |
| ***SC5DL*** | 6309 | 11q23.3 | Sterol-C5-desaturase (ERG3 delta-5-desaturase homolog, S. cerevisiae)-like. This gene encodes an enzyme of cholesterol biosynthesis. The encoded protein catalyzes the conversion of lathosterol into 7-dehydrocholesterol. Mutations in this gene have been associated with lathosterolosis. Alternatively spliced transcript variants encoding the same protein have been described. | Oxidase | Other steroid metabolism | N/A |
| ***SCD4*** | 79966 | 4q21.3 | Same as SCD5 or stearoyl-CoA desaturase 5. Stearoyl-CoA desaturase (SCD; EC 1.14.99.5) is an integral membrane protein of the endoplasmic reticulum that catalyzes the formation of monounsaturated fatty acids from saturated fatty acids. SCD may be a key regulator of energy metabolism with a role in obesity and dislipidemia. Four SCD isoforms, Scd1 through Scd4, have been identified in mouse. In contrast, only 2 SCD isoforms, SCD1 (MIM 604031) and SCD5, have been identified in human. SCD1 shares about 85% amino acid identity with all 4 mouse SCD isoforms, as well as with rat Scd1 and Scd2. In contrast, SCD5 shares limited homology with the rodent SCDs and appears to be unique to primates. | Other oxidoreductase | Fatty acid metabolism | N/A |
| ***SET*** | 6418 | 9q34 | SET translocation (myeloid leukemia-associated). SET protein regulates G(2)/M transition by modulating cyclin B-CDK1 activity. Set/TAF-Ibeta and pp32 proteins have roles as transducers of chromatin signaling by integrating chromatin hypoacetylation and transcriptional repression. Results suggest that SET is part of both IFN-gamma-mediated and stress-mediated cellular responses and that SET induces cell differentiation via calcium and MAPK/ERK pathways | Phosphatase inhibitor | DNA replication; Chromatin packaging and remodeling; Apoptosis; DNA replication | N/A |
| ***SHFM3*** | 6468 | 10q24 | Same as FBXW4 or F-box and WD repeat domain containing 4. This gene is a member of the F-box/WD-40 gene family, which recruit specific target proteins through their WD-40 protein-protein binding domains for ubiquitin mediated degradation. In mouse, a highly similar protein is thought to be responsible for maintaining the apical ectodermal ridge of developing limb buds; disruption of the mouse gene results in the absence of central digits, underdeveloped or absent metacarpal/metatarsal bones and syndactyly. This phenotype is remarkably similar to split hand-split foot malformation in humans, a clinically heterogeneous condition with a variety of modes of transmission. An autosomal recessive form has been mapped to the chromosomal region where this gene is located, and complex rearrangements involving duplications of this gene and others have been associated with the condition. A pseudogene of this locus has been mapped to one of the introns of the BCR gene on chromosome 22. | Other miscellaneous function protein | Other protein metabolism; Other developmental process | N/A |
| ***SHH*** | 6469 | 7q36 | Sonic hedgehog homolog (Drosophila). This gene encodes a protein that is instrumental in patterning the early embryo. It has been implicated as the key inductive signal in patterning of the ventral neural tube, the anterior-posterior limb axis, and the ventral somites. Of three human proteins showing sequence and functional similarity to the sonic hedgehog protein of Drosophila, this protein is the most similar. The protein is made as a precursor that is autocatalytically cleaved; the N-terminal portion is soluble and contains the signalling activity while the C-terminal portion is involved in precursor processing. More importantly, the C-terminal product covalently attaches a cholesterol moiety to the N-terminal product, restricting the N-terminal product to the cell surface and preventing it from freely diffusing throughout the developing embryo. Defects in this protein or in its signalling pathway are a cause of holoprosencephaly (HPE), a disorder in which the developing forebrain fails to correctly separate into right and left hemispheres. HPE is manifested by facial deformities. It is also thought that mutations in this gene or in its signalling pathway may be responsible for VACTERL syndrome, which is characterized by vertebral defects, anal atresia, tracheoesophageal fistula with esophageal atresia, radial and renal dysplasia, cardiac anomalies, and limb abnormalities. Additionally, mutations in a long range enhancer located approximately 1 megabase upstream of this gene disrupt limb patterning and can result in preaxial polydactyly. | Other signaling molecule; Protease | Proteolysis; Other receptor mediated signaling pathway; Other intracellular signaling cascade; Ligand-mediated signaling; Embryogenesis; Cell cycle; Oncogene; Other oncogenesis | Hedgehog signaling pathway->Hedgehog |
| ***SHMT1*** | 6470 | 17p11.2 | Serine hydroxymethyltransferase 1 (soluble). This gene encodes the cellular form of serine hydroxymethyltransferase, a pyridoxal phosphate-containing enzyme that catalyzes the reversible conversion of serine and tetrahydrofolate to glycine and 5,10-methylene tetrahydrofolate. This reaction provides one carbon units for synthesis of methionine, thymidylate, and purines in the cytoplasm. This gene is located within the Smith-Magenis syndrome region on chromosome 17. Alternative splicing of this gene results in 2 transcript variants encoding 2 different isoforms. Additional transcript variants have been described, but their biological validity has not been determined. | Methyltransferase | Other amino acid metabolism; Other nucleoside, nucleotide and nucleic acid metabolism | Serine glycine biosynthesis->Glycine hydroxymethyltransferase |
| ***SHMT2*** | 6472 | 12q12-q14 | Serine hydroxymethyltransferase 2 (mitochondrial) | Methyltransferase | Other amino acid metabolism; Other nucleoside, nucleotide and nucleic acid metabolism | Serine glycine biosynthesis->Glycine hydroxymethyltransferase |
| ***SIX3*** | 6496 | 2p16-p21 | Sine oculis homeobox homolog 3 (Drosophila). Six3 gene is regulated by Pax6, Prox 1, and Msx2 transcription factors. Occurrence of aprosencephaly/atelencephaly and holoprosencephaly in a family with a SIX3 gene mutation. Different SIX3 mutations in HPE2 may affect different signaling pathways, and that one of these pathways may involve the nuclear receptor NOR1. The homeotic protein Six3 is a coactivator of the nuclear receptor NOR-1 and a corepressor of the fusion protein EWS/NOR-1 in human extraskeletal myxoid chondrosarcomas. | Molecular function unclassified | Biological process unclassified | N/A |
| ***SKI*** | 6497 | 1p36.33 | v-ski sarcoma viral oncogene homolog (avian). Too many to enumerate here. Check Entrez Gene. | Other transcription factor | mRNA transcription regulation; Neurogenesis; Muscle development; Cell cycle control; Oncogene | TGF-beta signaling pathway->Co-activators or corepressors |
| ***SLC7A11*** | 23657 | 4q28-q32 | Solute carrier family 7, (cationic amino acid transporter, y+ system) member 11. SLC7A11 is a member of a heteromeric Na(+)-independent anionic amino acid transport system highly specific for cystine and glutamate. In this system, designated system Xc(-), the anionic form of cystine is transported in exchange for glutamate. | Amino acid transporter | Amino acid transport; Transport | N/A |
| ***SMAD1*** | 4086 | 4q31 | SMAD family member 1. The protein encoded by this gene belongs to the SMAD, a family of proteins similar to the gene products of the Drosophila gene 'mothers against decapentaplegic' (Mad) and the C. elegans gene Sma. SMAD proteins are signal transducers and transcriptional modulators that mediate multiple signaling pathways. This protein mediates the signals of the bone morphogenetic proteins (BMPs), which are involved in a range of biological activities including cell growth, apoptosis, morphogenesis, development and immune responses. In response to BMP ligands, this protein can be phosphorylated and activated by the BMP receptor kinase. The phosphorylated form of this protein forms a complex with SMAD4, which is important for its function in the transcription regulation. This protein is a target for SMAD-specific E3 ubiquitin ligases, such as SMURF1 and SMURF2, and undergoes ubiquitination and proteasome-mediated degradation. Alternatively spliced transcript variants encoding the same protein have been observed. | Other transcription factor | mRNA transcription regulation; Receptor protein serine/threonine kinase signaling pathway; Other intracellular signaling cascade; Ligand-mediated signaling; Developmental processes; Oncogenesis | Wnt signaling pathway->Mothers against decapentaplegic homolog 4; TGF-beta signaling pathway->RSmads |
| ***SMAD2*** | 4087 | 18q21.1 | SMAD family member 2. The protein encoded by this gene belongs to the SMAD, a family of proteins similar to the gene products of the Drosophila gene 'mothers against decapentaplegic' (Mad) and the C. elegans gene Sma. SMAD proteins are signal transducers and transcriptional modulators that mediate multiple signaling pathways. This protein mediates the signal of the transforming growth factor (TGF)-beta, and thus regulates multiple cellular processes, such as cell proliferation, apoptosis, and differentiation. This protein is recruited to the TGF-beta receptors through its interaction with the SMAD anchor for receptor activation (SARA) protein. In response to TGF-beta signal, this protein is phosphorylated by the TGF-beta receptors. The phosphorylation induces the dissociation of this protein with SARA and the association with the family member SMAD4. The association with SMAD4 is important for the translocation of this protein into the nucleus, where it binds to target promoters and forms a transcription repressor complex with other cofactors. This protein can also be phosphorylated by activin type 1 receptor kinase, and mediates the signal from the activin. Alternatively spliced transcript variants encoding the same protein have been observed. | Other transcription factor | mRNA transcription regulation; Receptor protein serine/threonine kinase signaling pathway; Other intracellular signaling cascade; Ligand-mediated signaling; Developmental processes; Oncogenesis | Wnt signaling pathway->Mothers against decapentaplegic homolog 4; TGF-beta signaling pathway->RSmads |
| ***SMAD3*** | 4088 | 15q22.33 | SMAD family member 3. Too many to enumerate here. Refer to Entrez Gene. Smad3 trimerization, induced by phosphorylation, activates the TGF-beta signal by driving Smad3 dissociation from SARA & sets up the negative feedback mechanism by Ski. | Other transcription factor | mRNA transcription regulation; Receptor protein serine/threonine kinase signaling pathway; Other intracellular signaling cascade; Ligand-mediated signaling; Developmental processes; Oncogenesis | Wnt signaling pathway->Mothers against decapentaplegic homolog 4; TGF-beta signaling pathway->RSmads |
| ***SMAD4*** | 4089 | 18q21.1 | SMAD family member 4. Too many to enumerate here. Refer to Entrez Gene. TGF-beta signaling suppresses nuclear export of Smad4 by chromosome region maintenance 1 and targets Smad4 into the nucleus; mutations in Smad4 that affect its interaction with Smad2 or Smad3 impair nuclear accumulation of Smad4 in response to TGF-beta | Other transcription factor; Nucleic acid binding | mRNA transcription regulation; Receptor protein serine/threonine kinase signaling pathway; Other intracellular signaling cascade; Ligand-mediated signaling; Developmental processes; Cell proliferation and differentiation; Oncogenesis | Wnt signaling pathway->Mothers against decapentaplegic homolog 4; TGF-beta signaling pathway->parter Smads |
| ***SMS*** | 6611 | Xp22.1 | Spermine synthase. The protein encoded by this gene belongs to the spermidine/spermine synthases family. This gene encodes a ubiquitous enzyme of polyamine metabolism. | Synthase | Other metabolism | N/A |
| ***SNAI2*** | 6591 | 8q11 | Snail homolog 2 (Drosophila). This gene encodes a member of the Snail family of C2H2-type zinc finger transcription factors. The encoded protein acts as a transcriptional repressor that binds to E-box motifs and is also likely to repress E-cadherin transcription in breast carcinoma. This protein is involved in epithelial-mesenchymal transitions and has antiapoptotic activity. Mutations in this gene may be associated with sporatic cases of neural tube defects. | Zinc finger transcription factor; Other DNA-binding protein | mRNA transcription regulation | N/A |
| ***SNAI1*** | 6615 | 20q13.1-q13.2 | Snail homolog 1 (Drosophila). The Drosophila embryonic protein snail is a zinc finger transcriptional repressor which downregulates the expression of ectodermal genes within the mesoderm. The nuclear protein encoded by this gene is structurally similar to the Drosophila snail protein, and is also thought to be critical for mesoderm formation in the developing embryo. At least two variants of a similar processed pseudogene have been found on chromosome 2. | Zinc finger transcription factor; Other DNA-binding protein | mRNA transcription regulation | N/A |
| ***SNX3*** | 8724 | 6q21 | Sorting nexin 3. This gene encodes a member of the sorting nexin family. Members of this family contain a phox (PX) domain, which is a phosphoinositide binding domain, and are involved in intracellular trafficking. This protein does not contain a coiled coil region, like most family members. This protein interacts with phosphatidylinositol-3-phosphate, and is involved in protein trafficking. | Molecular function unclassified | Biological process unclassified | N/A |
| ***SOX1*** | 6656 | 13q34 | SRY (sex determining region Y)-box 1. This intronless gene encodes a member of the SOX (SRY-related HMG-box) family of transcription factors involved in the regulation of embryonic development and in the determination of the cell fate. The encoded protein may act as a transcriptional activator after forming a protein complex with other proteins. In mice, a similar protein regulates the gamma-crystallin genes and is essential for lens development. | HMG box transcription factor; Nucleic acid binding | mRNA transcription regulation | N/A |
| ***SOX5*** | 6660 | 12p12.1 | SRY (sex determining region Y)-box 5. This gene encodes a member of the SOX (SRY-related HMG-box) family of transcription factors involved in the regulation of embryonic development and in the determination of the cell fate. The encoded protein may act as a transcriptional regulator after forming a protein complex with other proteins. The encoded protein may play a role in chondrogenesis. A pseudogene of this gene is located on chromosome 8. Multiple transcript variants encoding distinct isoforms have been identified for this gene. | HMG box transcription factor; Nucleic acid binding | mRNA transcription regulation | N/A |
| ***SOX9*** | 6662 | 17q24.3-q25.1 | SRY (sex determining region Y)-box 9 (campomelic dysplasia, autosomal sex-reversal). The protein encoded by this gene recognizes the sequence CCTTGAG along with other members of the HMG-box class DNA-binding proteins. It acts during chondrocyte differentiation and, with steroidogenic factor 1, regulates transcription of the anti-Muellerian hormone (AMH) gene. Deficiencies lead to the skeletal malformation syndrome campomelic dysplasia, frequently with sex reversal. | HMG box transcription factor; Nucleic acid binding | mRNA transcription regulation | N/A |
| ***SP8*** | 221833 | 7p21.2 | Sp8 transcription factor | Zinc finger transcription factor | mRNA transcription regulation | Huntington disease->Sp1 |
| ***SPAM1*** | 6677 | 7q31.3 | Sperm adhesion molecule 1 (PH-20 hyaluronidase, zona pellucida binding). Hyaluronidase degrades hyaluronic acid, a major structural proteoglycan found in extracellular matrices and basement membranes. Six members of the hyaluronidase family are clustered into two tightly linked groups on chromosome 3p21.3 and 7q31.3. This gene was previously referred to as HYAL1 and HYA1 and has since been assigned the official symbol SPAM1; another family member on chromosome 3p21.3 has been assigned HYAL1. This gene encodes a GPI-anchored enzyme located on the human sperm surface and inner acrosomal membrane. This multifunctional protein is a hyaluronidase that enables sperm to penetrate through the hyaluronic acid-rich cumulus cell layer surrounding the oocyte, a receptor that plays a role in hyaluronic acid induced cell signaling, and a receptor that is involved in sperm-zona pellucida adhesion. Abnormal expression of this gene in tumors has implicated this protein in degradation of basement membranes leading to tumor invasion and metastasis. Multiple protein isoforms are encoded by transcript variants of this gene. | Receptor; Glycosidase | Other polysaccharide metabolism; Cell adhesion; Fertilization | N/A |
| ***SPP1*** | 6696 | 4q21-q25 | Secreted phosphoprotein 1 (osteopontin, bone sialoprotein I, early T-lymphocyte activation 1) | Other cytokine; Cell adhesion molecule; Defense/immunity protein; Other extracellular matrix | Cell adhesion; Cell structure N/A |
| ***SPPL3*** | 121665 | 12q24.31 | Signal peptide peptidase 3. SPPL2b is targeted through the secretory pathway to endosomes/lysosomes, whereas SPP and SPPL3 are restricted to the ER. SPPL3 cleaves a SPP substrate, but a more distantly related homologue SPPL2b does not, providing strong evidence that the malaria SPP and human SPPL3 have conserved active sites, while the active sites SPPL2b is distinct. SPP, SPPL2a, -2b, -2c, and -3 probably cleave type II-oriented substrate peptides as shown by consensus analysis. | Aspartic protease | Other intracellular signaling cascade | N/A |
| ***SPRY2*** | 10253 | 13q31.1 | Sprouty homolog 2 (Drosophila). This gene encodes a protein belonging to the sprouty family. The encoded protein contains a carboxyl-terminal cysteine-rich domain essential for the inhibitory activity on receptor tyrosine kinase signaling proteins and is required for growth factor stimulated translocation of the protein to membrane ruffles. In primary dermal endothelial cells this gene is transiently upregulated in response to fibroblast growth factor two. This protein is indirectly involved in the non-cell autonomous inhibitory effect on fibroblast growth factor two signaling. The protein interacts with Cas-Br-M (murine) ectropic retroviral transforming sequence, and can function as a bimodal regulator of epidermal growth factor receptor/mitogen-activated protein kinase signaling. This protein may play a role in alveoli branching during lung development as shown by a similar mouse protein. | Other signaling molecule; Other transcription factor | Receptor protein tyrosine kinase signaling pathway; MAPKKK cascade; Mesoderm development | FGF signaling pathway->sprouty; EGF receptor signaling pathway->SPRY; EGF receptor signaling pathway->sprouty |
| ***SPTLC1*** | 10558 | 9q22.2 | Serine palmitoyltransferase, long chain base subunit 1. Serine palmitoyltransferase, which consists of two different subunits, is the key enzyme in sphingolipid biosynthesis. It converts L-serine and palmitoyl-CoA to 3-oxosphinganine with pyridoxal 5'-phosphate as a cofactor. The product of this gene is the long chain base subunit 1 of serine palmitoyltransferase. Mutations in this gene were identified in patients with hereditary sensory neuropathy type 1. Alternatively spliced variants encoding different isoforms have been identified. | Other transferase | Lipid metabolism | N/A |
| ***STAT3*** | 6774 | 17q21.31 | Signal transducer and activator of transcription 3 (acute-phase response factor). The protein encoded by this gene is a member of the STAT protein family. In response to cytokines and growth factors, STAT family members are phosphorylated by the receptor associated kinases, and then form homo- or heterodimers that translocate to the cell nucleus where they act as transcription activators. This protein is activated through phosphorylation in response to various cytokines and growth factors including IFNs, EGF, IL5, IL6, HGF, LIF and BMP2. This protein mediates the expression of a variety of genes in response to cell stimuli, and thus plays a key role in many cellular processes such as cell growth and apoptosis. The small GTPase Rac1 has been shown to bind and regulate the activity of this protein. PIAS3 protein is a specific inhibitor of this protein. Three alternatively spliced transcript variants encoding distinct isoforms have been described. | Other transcription factor; Nucleic acid binding | mRNA transcription regulation; JAK-STAT cascade; Stress response; Inhibition of apoptosis; Hematopoiesis; Other oncogenesis; Cell motility | Angiogenesis->Signal Transducer and Activator of Transcription 3; EGF receptor signaling pathway->signal transducer and activator of transcription; PDGF signaling pathway->STAT; Interleukin signaling pathway->Signal transducers and activators of transcription; Ras Pathway->Signal transducer and activator of transcription; JAK/STAT signaling pathway->Signal transducers and activators of transcription; Inflammation mediated by chemokine and cytokine signaling pathway->Signal transducers and activators of transcription |
| ***STX18*** | 53407 | 4p16.3-p16.2 | Syntaxin 18. RINT-1 coordinates the localization and function of ZW10 by serving as a link between ZW10 and the SNARE complex comprising syntaxin 18. | Transporter; SNARE protein | Ligand-mediated signaling; Regulated exocytosis; Transport; Neurotransmitter release | N/A |
| ***SULT1A1*** | 6817 | 16p12.1-p11.2 | Sulfotransferase family, cytosolic, 1A, phenol-preferring, member 1. Sulfotransferase enzymes catalyze the sulfate conjugation of many hormones, neurotransmitters, drugs, and xenobiotic compounds. These cytosolic enzymes are different in their tissue distributions and substrate specificities. The gene structure (number and length of exons) is similar among family members. This gene encodes one of two phenol sulfotransferases with thermostable enzyme activity. Multiple alternatively spliced variants that encode two isoforms have been identified for this gene. | Other transferase | Steroid hormone metabolism; Sulfur metabolism | N/A |
| ***SUMO1*** | 7341 | 2q33 | SMT3 suppressor of mif two 3 homolog 1 (S. cerevisiae). This gene encodes a protein that is a member of the SUMO (small ubiquitin-like modifier) protein family. It functions in a manner similar to ubiquitin in that it is bound to target proteins as part of a post-translational modification system. However, unlike ubiquitin which targets proteins for degradation, this protein is involved in a variety of cellular processes, such as nuclear transport, transcriptional regulation, apoptosis, and protein stability. It is not active until the last four amino acids of the carboxy-terminus have been cleaved off. Several pseudogenes have been reported for this gene. Alternate transcriptional splice variants encoding different isoforms have been characterized. | Other miscellaneous function protein | Protein modification; Inhibition of apoptosis; Chromosome segregation; Miscellaneous | p53 pathway->Small ubiquitin-like modifier 1 |
| ***TBX1*** | 6899 | 22q11 | T-box 1. This gene is a member of a phylogenetically conserved family of genes that share a common DNA-binding domain, the T-box. T-box genes encode transcription factors involved in the regulation of developmental processes. This gene product shares 98% amino acid sequence identity with the mouse ortholog. DiGeorge syndrome (DGS)/velocardiofacial syndrome (VCFS), a common congenital disorder characterized by neural-crest-related developmental defects, has been associated with deletions of chromosome 22q11.2, where this gene has been mapped. Studies using mouse models of DiGeorge syndrome suggest a major role for this gene in the molecular etiology of DGS/VCFS. Several alternatively spliced transcript variants encoding different isoforms have been described for this gene. | Other transcription factor; Nucleic acid binding | mRNA transcription regulation; Developmental processes | N/A |
| ***TBX10*** | 347853 | 11q13.1 | T-box 10 | Other transcription factor; Nucleic acid binding | mRNA transcription regulation; Developmental processes | N/A |
| ***TBX15*** | 6913 | 1p11.1 | T-box 15 | Other transcription factor; Nucleic acid binding | mRNA transcription regulation; Developmental processes | N/A |
| ***TBX21*** | 30009 | 17q21.32 | T-box 21. This gene is a member of a phylogenetically conserved family of genes that share a common DNA-binding domain, the T-box. T-box genes encode transcription factors involved in the regulation of developmental processes. This gene is the human ortholog of mouse Tbx21/Tbet gene. Studies in mouse show that Tbx21 protein is a Th1 cell-specific transcription factor that controls the expression of the hallmark Th1 cytokine, interferon-gamma (IFNG). Expression of the human ortholog also correlates with IFNG expression in Th1 and natural killer cells, suggesting a role for this gene in initiating Th1 lineage development from naive Th precursor cells. | Other transcription factor; Nucleic acid binding | mRNA transcription regulation; T-cell mediated immunity; Cell proliferation and differentiation | N/A |
| ***TBX22*** | 50945 | Xq21.1 | T-box 22. This gene is a member of a phylogenetically conserved family of genes that share a common DNA-binding domain, the T-box. T-box genes encode transcription factors involved in the regulation of developmental processes. Mutations in this gene have been associated with the inherited X-linked disorder, Cleft palate with ankyloglossia, and it is believed to play a major role in human palatogenesis. | Other transcription factor; Nucleic acid binding | mRNA transcription regulation; T-cell mediated immunity; Cell proliferation and differentiation | N/A |
| ***TBX4*** | 9496 | 17q21-q22 | T-box 4. This gene is a member of a phylogenetically conserved family of genes that share a common DNA-binding domain, the T-box. T-box genes encode transcription factors involved in the regulation of developmental processes. This gene is the human homolog of mouse Tbx4, which is closely linked to Tbx2 on mouse chromosome 11. Similarly this gene, like TBX2, maps to human chromosome 17. Expression studies in mouse and chicken show that Tbx4 is expressed in developing hindlimb, but not in forelimb buds, suggesting a role for this gene in regulating limb development and specification of limb identity. | Other transcription factor; Nucleic acid binding | mRNA transcription regulation; Mesoderm development | N/A |
| ***TCF1*** | 6927 | 12q24.31 | Transcription factor 1, hepatic; LF-B1, hepatic nuclear factor (HNF1), albumin proximal factor. Too many to enumerate here. See Entrez Gene. | Other transcription factor; Nucleic acid binding | mRNA transcription regulation | N/A |
| ***TCF21*** | 6943 | 6pter-qter | Transcription factor 21. TCF21 encodes a transcription factor of the basic helix-loop-helix family. The TCF21 product is mesoderm specific, and expressed in embryonic epicardium, mesenchyme-derived tissues of lung, gut, gonad, and both mesenchymal and glomerular epithelial cells in the kidney. Two transcript variants encoding the same protein have been found for this gene. | Basic helix-loop-helix transcription factor; Nucleic acid binding | mRNA transcription regulation | N/A |
| ***TCOF1*** | 6949 | 5q33.1 | Treacher Collins-Franceschetti syndrome 1. This gene encodes a nucleolar protein with a LIS1 homology domain. The protein is involved in ribosomal DNA gene transcription through its interaction with upstream binding factor (UBF). Mutations in this gene have been associated with Treacher Collins syndrome, a disorder which includes abnormal craniofacial development. Alternate transcriptional splice variants encoding different isoforms have been found for this gene, but only three of them have been characterized to date. | Transporter | Apoptosis; Hearing; Neurogenesis | N/A |
| ***TFAP2A*** | 7020 | 6p24 | Transcription factor AP-2 alpha (activating enhancer binding protein 2 alpha). AP2-alpha is a 52-kD retinoic acid-inducible and developmentally regulated activator of transcription that binds to a consensus DNA-binding sequence CCCCAGGC in the SV40 and metallothionein (MIM 156350) promoters. | Other transcription factor | mRNA transcription regulation; Ectoderm development; Oncogenesis | N/A |
| ***TGFA*** | 7039 | 2p13 | Transforming growth factor, alpha. Transforming growth factors (TGFs) are biologically active polypeptides that reversibly confer the transformed phenotype on cultured cells. TGF-alpha shows about 40% sequence homology with epidermal growth factor (EGF; MIM 131530) and competes with EGF for binding to the EGF receptor (MIM 131550), stimulating its phosphorylation and producing a mitogenic response. | Other cytokine | Cytokine and chemokine mediated signaling pathway; Ligand-mediated signaling; Cell cycle control; Cell proliferation and differentiation | EGF receptor signaling pathway->Epidermal growth factor |
| ***TGFB1*** | 7040 | 19q13.2 | Transforming growth factor, beta 1. TGFB is a multifunctional peptide that controls proliferation, differentiation, and other functions in many cell types. TGFB acts synergistically with TGFA (MIM 190170) in inducing transformation. It also acts as a negative autocrine growth factor. Dysregulation of TGFB activation and signaling may result in apoptosis. Many cells synthesize TGFB and almost all of them have specific receptors for this peptide. TGFB1, TGFB2 (MIM 190220), and TGFB3 (MIM 190230) all function through the same receptor signaling systems. | Growth factor | Other receptor mediated signaling pathway; Developmental processes; Cell proliferation and differentiation | TGF-beta signaling pathway->Transforming growth factor beta |
| ***TGFB2*** | 7042 | 1q41 | Transforming growth factor, beta 2. Too many to enumerate. See Entrez Gene. TGFB2 induces MMP-2 expression and suppresses TIMP-2 expression. It promotes invasion of cultured glioma cells in a dose-dependent way. | Growth factor | Other receptor mediated signaling pathway; Developmental processes; Cell proliferation and differentiation | TGF-beta signaling pathway->Transforming growth factor beta |
| ***TGFB3*** | 7043 | 14q24 | Transforming growth factor, beta 3. Too many to enumerate. See Entrez Gene. CD24 regulates E-cadherin and TGF-beta3 expression in cultured oral epithelial cells. Glucocorticoids significantly inhibited TGF-beta1 and TGF-beta2 production and reduced expression of the up-regulated TGF-beta1 and TGF-beta2 mRNA induced by exogenous TGF-beta1, -beta2, or -beta3 | Growth factor | Other receptor mediated signaling pathway; Developmental processes; Cell proliferation and differentiation | TGF-beta signaling pathway->Transforming growth factor beta |
| ***TGFBR1*** | 7046 | 9q22.33 | Transforming growth factor, beta receptor I (activin A receptor type II-like kinase, 53kDa). The protein encoded by this gene forms a heteromeric complex with type II TGF-beta receptors when bound to TGF-beta, transducing the TGF-beta signal from the cell surface to the cytoplasm. The encoded protein is a serine/threonine protein kinase. Mutations in this gene have been associated with Loeys-Dietz aortic aneurysm syndrome (LDAS). | Molecular function unclassified | Biological process unclassified | N/A |
| ***TGFBR2*** | 7048 | 3p24.1 | Transforming growth factor, beta receptor II (70/80kDa). This gene encodes a member of the Ser/Thr protein kinase family and the TGFB receptor subfamily. The encoded protein is a transmembrane protein that has a protein kinase domain, forms a heterodimeric complex with another receptor protein, and binds TGF-beta. This receptor/ligand complex phosphorylates proteins, which then enter the nucleus and regulate the transcription of a subset of genes related to cell proliferation. Mutations in this gene have been associated with Marfan Syndrome, Loeys-Deitz Aortic Aneurysm Syndrome, and the development of various types of tumors. Alternatively spliced transcript variants encoding different isoforms have been characterized. | Other cytokine receptor; Serine/threonine protein kinase receptor; Protein kinase | Protein phosphorylation; Cytokine and chemokine mediated signaling pathway; Receptor protein serine/threonine kinase signaling pathway; Other developmental process | TGF-beta signaling pathway |
| ***TGFBR3*** | 7049 | 1p33-p32 | Transforming growth factor, beta receptor III. Too many to enumerate here. Refer to Entrez Gene. | TGF-beta receptor | Cytokine and chemokine mediated signaling pathway; Receptor protein serine/threonine kinase signaling pathway | N/A |
| ***TGIF*** | 7050 | 18p11.3 | TGFB-induced factor homeobox 1. The protein encoded by this gene is a member of the three-amino acid loop extension (TALE) superclass of atypical homeodomains. TALE homeobox proteins are highly conserved transcription regulators. This particular homeodomain binds to a previously characterized retinoid X receptor responsive element from the cellular retinol-binding protein II promoter. In addition to its role in inhibiting 9-cis-retinoic acid-dependent RXR alpha transcription activation of the retinoic acid responsive element, the protein is an active transcriptional co-repressor of SMAD2 and may participate in the transmission of nuclear signals during development and in the adult. Mutations in this gene are associated with holoprosencephaly type 4, which is a structural anomaly of the brain. Alternative splicing has been observed at this locus and eight variants, encoding four distinct isoforms, are described. | Homeobox transcription factor; Nucleic acid binding | mRNA transcription regulation | N/A |
| ***THRB*** | 7068 | 3p24.3 | Thyroid hormone receptor, beta (erythroblastic leukemia viral (v-erb-a) oncogene homolog 2, avian). The protein encoded by this gene is a nuclear hormone receptor for triiodothyronine. It is one of the several receptors for thyroid hormone, and has been shown to mediate the biological activities of thyroid hormone. Knockout studies in mice suggest that the different receptors, while having certain extent of redundancy, may mediate different functions of thyroid hormone. Defects in this gene are known to be a cause of generalized thyroid hormone resistance (GTHR), a syndrome characterized by goiter and high levels of circulating thyroid hormone (T3-T4), with normal or slightly elevated thyroid stimulating hormone (TSH). Several transcript variants have been observed for this gene, but the full-length nature of only one has been determined so far. | Nuclear hormone receptor; Transcription factor | mRNA transcription regulation; Steroid hormone-mediated signaling; Sensory perception; Developmental processes | N/A |
| ***TIMP2*** | 7077 | [17q25](http://www.ncbi.nlm.nih.gov/mapview/maps.cgi?ORG=hum&chr=17&maps=cntg-r,clone,model,ugHs,loc&VERBOSE=ON&cmd=focus&fill=40&size=40&query=TIMP2%5BSYM%5D) | TIMP metallopeptidase inhibitor 2. This gene is a member of the TIMP gene family. The proteins encoded by this gene family are natural inhibitors of the matrix metalloproteinases, a group of peptidases involved in degradation of the extracellular matrix. In addition to an inhibitory role against metalloproteinases, the encoded protein has a unique role among TIMP family members in its ability to directly suppress the proliferation of endothelial cells. As a result, the encoded protein may be critical to the maintenance of tissue homeostasis by suppressing the proliferation of quiescent tissues in response to angiogenic factors, and by inhibiting protease activity in tissues undergoing remodelling of the extracellular matrix. | Metalloprotease inhibitor | Proteolysis | N/A |
| ***TNFRSF10B*** | 8795 | 8p22-p21 | Tumor necrosis factor receptor superfamily, member 10b. The protein encoded by this gene is a member of the TNF-receptor superfamily, and contains an intracelluar death domain. This receptor can be activated by tumor necrosis factor-related apoptosis inducing ligand (TNFSF10/TRAIL/APO-2L), and transduces apoptosis signal. Studies with FADD-deficient mice suggested that FADD, a death domain containing adaptor protein, is required for the apoptosis mediated by this protein. | Tumor necrosis factor receptor | Other receptor mediated signaling pathway; Induction of apoptosis | p53 pathway->KILLER/death receptor (DR)5; Apoptosis signaling pathway->TRAIL-receptor |
| ***TNNT3*** | 7140 | 11p15.5 | Troponin T type 3 (skeletal, fast). These results indicate that the f peptide confers an inhibitory effect on the biological function of fast skeletal troponin T and this can be correlated with changes in the Ca2+ regulation associated with development in fast skeletal muscle. | Actin binding motor protein | Muscle contraction | N/A |
| ***TP63*** | 8626 | 3q27 | Tumor protein p73-like. Too many to enumerate here. See Entrez Gene. A common role is shown for p63 in classical EEC syndrome, both familial and sporadic, but not in other related or non-syndromic forms of orofacial clefts. | Other transcription factor | DNA repair; mRNA transcription regulation; Induction of apoptosis; Cell cycle control; Cell proliferation and differentiation; Tumor suppressor | P53 pathway |
| ***TRPS1*** | 7227 | 8q24.12 | Trichorhinophalangeal syndrome I. TRPS-1 protein and gene were expressed in >90% of early- and late-stage breast cancer, including ductal carcinoma in situ and invasive ductal, lobular, and papillary carcinomas. Novel and recurrent mutations responsible for the tricho-rhino-phalangeal syndromes. TRPS-1 protein and gene were expressed in >90% of early- and late-stage breast cancer, including ductal carcinoma in situ and invasive ductal, lobular, and papillary carcinomas. | Transcription factor; Nuclease | Nucleoside, nucleotide and nucleic acid metabolism; Other metabolism | N/A |
| ***TULP3*** | 7289 | [12p13.3](http://www.ncbi.nlm.nih.gov/mapview/maps.cgi?ORG=hum&chr=12&maps=cntg-r,clone,model,ugHs,loc&VERBOSE=ON&cmd=focus&fill=40&size=40&query=TULP3%5BSYM%5D) | Tubby like protein 3. This gene encodes a member of the tubby-like protein (TULP) family. Members of this family have been identified in plants, vertebrates, and invertebrates, and they share a conserved C-terminal region of approximately 200 amino acid residues. The human and mouse TULP3 proteins share 69% amino acid sequence identity, with higher identity at the N and C termini than in the central region. | Molecular function unclassified | Neurogenesis | N/A |
| ***TWIST1*** | 7291 | 7p21.2 | Twist homolog 1 (acrocephalosyndactyly 3; Saethre-Chotzen syndrome) (Drosophila). Basic helix-loop-helix (bHLH) transcription factors have been implicated in cell lineage determination and differentiation. The protein encoded by this gene is a bHLH transcription factor and shares similarity with another bHLH transcription factor, Dermo1. The strongest expression of this mRNA is in placental tissue; in adults, mesodermally derived tissues express this mRNA preferentially. Mutations in this gene have been found in patients with Saethre-Chotzen syndrome. | Basic helix-loop-helix transcription factor; Nucleic acid binding | mRNA transcription regulation; Developmental processes; Cell proliferation and differentiation | N/A |
| ***TYMS*** | 7298 | 18p11.32 | Thymidylate synthetase. Thymidylate synthase (TYMS, or TS; EC 2.1.1.45) uses the 5,10-methylenetetrahydrofolate (methylene-THF) as a cofactor to maintain the dTMP (thymidine-5-prime monophosphate) pool critical for DNA replication and repair. The enzyme has been of interest as a target for cancer chemotherapeutic agents. It is considered to be the primary site of action for 5-fluorouracil, 5-fluoro-2-prime-deoxyuridine, and some folate analogs. | Synthase; Methyltransferase | Pyrimidine metabolism; DNA metabolism; Other carbon metabolism | Formyltetrahydroformate biosynthesis->Dihydrofolate reductase; Formyltetrahydroformate biosynthesis->Thymidylate synthase; De novo pyrimidine deoxyribonucleotide biosynthesis->Thymidylate synthase; Folate biosynthesis->Dihydrofolate reductase; Folate biosynthesis->Dihydrofolate synthase |
| ***UFD1L*** | 7353 | 22q11.21 | Ubiquitin fusion degradation 1 like (yeast). The protein encoded by this gene forms a complex with two other proteins, NPL4 and VCP, which is necessary for the degradation of ubiquitinated proteins. In addition, this complex controls the disassembly of the mitotic spindle and the formation of a closed nuclear envelope after mitosis. Mutations in this gene have been associated with Catch 22 syndrome as well as cardiac and craniofacial defects. Alternative splicing results in multiple transcript variants encoding different isoforms. | Select regulatory molecule | Pre-mRNA processing; Proteolysis; Transport | N/A |
| ***UGT1A7*** | 54577 | 2q37 | UDP glucuronosyltransferase 1 family, polypeptide A7. This gene encodes a UDP-glucuronosyltransferase, an enzyme of the glucuronidation pathway that transforms small lipophilic molecules, such as steroids, bilirubin, hormones, and drugs, into water-soluble, excretable metabolites. This gene is part of a complex locus that encodes several UDP-glucuronosyltransferases. The locus includes thirteen unique alternate first exons followed by four common exons. Four of the alternate first exons are considered pseudogenes. Each of the remaining nine 5' exons may be spliced to the four common exons, resulting in nine proteins with different N-termini and identical C-termini. Each first exon encodes the substrate binding site, and is regulated by its own promoter. The enzyme encoded by this gene has moderate glucuronidase activity with phenols. | Glycosyltransferase | Other polysaccharide metabolism; Steroid hormone metabolism | N/A |
| ***VCL*** | 7414 | 10q22.1-q23 | Vinculin. Vinculin is a cytoskeletal protein associated with cell-cell and cell-matrix junctions, where it is thought to function as one of several interacting proteins involved in anchoring F-actin to the membrane. Multiple alternatively spliced transcript variants have been found for this gene, but the biological validity of some variants has not been determined. Human vinculin protein exhibits a greater than 95% sequence identity to the chicken vinculin protein. | Cell adhesion molecule; Non-motor actin binding protein | Cell structure | Integrin signalling pathway->Vinculin |
| ***WHSC1*** | 7468 | 4p16.3 | Wolf-Hirschhorn syndrome candidate 1. This gene encodes a protein that contains four domains present in other developmental proteins: a PWWP domain, an HMG box, a SET domain, and a PHD-type zinc finger. It is expressed ubiquitously in early development. Wolf-Hirschhorn syndrome (WHS) is a malformation syndrome associated with a hemizygous deletion of the distal short arm of chromosome 4. This gene maps to the 165 kb WHS critical region and has also been involved in the chromosomal translocation t(4;14)(p16.3;q32.3) in multiple myelomas. Alternative splicing of this gene results in multiple transcript variants encoding different isoforms. Some transcript variants are nonsense-mediated mRNA (NMD) decay candidates, hence not represented as reference sequences. | Other DNA-binding protein; Methyltransferase | Chromatin packaging and remodeling | N/A |
| ***WNT3A*** | 89780 | 1q42 | Wingless-type MMTV integration site family, member 3A. The WNT gene family consists of structurally related genes which encode secreted signaling proteins. These proteins have been implicated in oncogenesis and in several developmental processes, including regulation of cell fate and patterning during embryogenesis. This gene is a member of the WNT gene family. It encodes a protein which shows 96% amino acid identity to mouse Wnt3A protein, and 84% to human WNT3 protein, another WNT gene product. This gene is clustered with WNT14 gene, another family member, in chromosome 1q42 region. | Other signaling molecule | Ligand-mediated signaling; Developmental processes | Cadherin signaling pathway; Wnt signaling pathway |
| ***WNT4*** | 54361 | 1p36.23-p35.1 | Wingless-type MMTV integration site family, member 4. The WNT gene family consists of structurally related genes which encode secreted signaling proteins. These proteins have been implicated in oncogenesis and in several developmental processes, including regulation of cell fate and patterning during embryogenesis. This gene is a member of the WNT gene family, and is the first signaling molecule shown to influence the sex-determination cascade. It encodes a protein which shows 98% amino acid identity to the Wnt4 protein of mouse and rat. This gene and DAX1, a gene known to antagonize the testis-determining factor, play a concerted role in both the control of female development and the prevention of testes formation. This gene and another two family members, WNT2 and WNT7B, may be associated with abnormal proliferation in breast tissue. | Other signaling molecule | Ligand-mediated signaling; Developmental processes | Cadherin signaling pathway; Wnt signaling pathway |
| ***WNT5A*** | 7474 | 3p21 | Wingless-type MMTV integration site family, member 5A. The WNT gene family consists of structurally related genes which encode secreted signaling proteins. These proteins have been implicated in oncogenesis and in several developmental processes, including regulation of cell fate and patterning during embryogenesis. This gene is a member of the WNT gene family. It encodes a protein which shows 98%, 98% and 87% amino acid identity to the mouse, rat and the xenopus Wnt5A protein, respectively. The experiments performed in Xenopus laevis embryos identified that human frizzled-5 (hFz5) is the receptor for the Wnt5A ligand and the Wnt5A/hFz5 signaling mediates axis induction. | Other signaling molecule | Ligand-mediated signaling; Developmental processes | Cadherin signaling pathway; Wnt signaling pathway |
| ***WNT6*** | 7475 | 2q35 | Wingless-type MMTV integration site family, member 6. The WNT gene family consists of structurally related genes which encode secreted signaling proteins. These proteins have been implicated in oncogenesis and in several developmental processes, including regulation of cell fate and patterning during embryogenesis. This gene is a member of the WNT gene family. It is overexpressed in cervical cancer cell line and strongly coexpressed with another family member, WNT10A, in colorectal cancer cell line. The gene overexpression may play key roles in carcinogenesis. This gene and the WNT10A gene are clustered in the chromosome 2q35 region. The protein encoded by this gene is 97% identical to the mouse Wnt6 protein at the amino acid level. | Other signaling molecule | Ligand-mediated signaling; Developmental processes | Cadherin signaling pathway; Wnt signaling pathway |
| ***WNT7B*** | 7477 | 22q13 | Wingless-type MMTV integration site family, member 7B. The WNT gene family consists of structurally related genes which encode secreted signaling proteins. These proteins have been implicated in oncogenesis and in several developmental processes, including regulation of cell fate and patterning during embryogenesis. This gene is a member of the WNT gene family. It encodes a protein which shows 99% and 91% amino acid identity to the mouse and Xenopus Wnt7A proteins, respectively. Among members of the human WNT family, this protein is most similar to WNT7A protein (77.1% total amino acid identity). This gene may play important roles in the development and progression of gastric cancer, esophageal cancer, and pancreatic cancer. | Other signaling molecule | Ligand-mediated signaling; Developmental processes | Cadherin signaling pathway; Wnt signaling pathway |
| ***WNT9B*** | 7484 | 17q21 | Wingless-type MMTV integration site family, member 9B.The WNT gene family consists of structurally related genes that encode secreted signaling proteins. These proteins have been implicated in oncogenesis and in several developmental processes, including regulation of cell fate and patterning during embryogenesis. This gene is a member of the WNT gene family. Study of its expression in the teratocarcinoma cell line NT2 suggests that it may be implicated in the early process of neuronal differentiation of NT2 cells induced by retinoic acid. This gene is clustered with WNT3, another family member, in the chromosome 17q21 region. | Other signaling molecule; Extracellular matrix structural protein | Ligand-mediated signaling | Cadherin signaling pathway; Wnt signaling pathway |
| ***XPD*** | 2068 | 19q13.3 | Same as ERCC2 or Excision repair cross-complementing rodent repair deficiency, complementation group 2 (xeroderma pigmentosum D). The nucleotide excision repair pathway is a mechanism to repair damage to DNA. The protein encoded by this gene is involved in transcription-coupled nucleotide excision repair and is an integral member of the basal transcription factor BTF2/TFIIH complex. The gene product has ATP-dependent DNA helicase activity and belongs to the RAD3/XPD subfamily of helicases. Defects in this gene can result in three different disorders, the cancer-prone syndrome xeroderma pigmentosum complementation group D, trichothiodystrophy, and Cockayne syndrome. | DNA helicase | DNA repair; mRNA transcription; Oncogenesis | N/A |
| ***XRCC1*** | 7515 | 19q13.2 | X-ray repair complementing defective repair in Chinese hamster cells 1. The protein encoded by this gene is involved in the efficient repair of DNA single-strand breaks formed by exposure to ionizing radiation and alkylating agents. This protein interacts with DNA ligase III, polymerase beta and poly (ADP-ribose) polymerase to participate in the base excision repair pathway. It may play a role in DNA processing during meiogenesis and recombination in germ cells. A rare microsatellite polymorphism in this gene is associated with cancer in patients of varying radiosensitivity. | Nucleic acid binding | DNA repair | N/A |
| ***XRCC3*** | 7517 | 14q32.3 | X-ray repair complementing defective repair in Chinese hamster cells 3. This gene encodes a member of the RecA/Rad51-related protein family that participates in homologous recombination to maintain chromosome stability and repair DNA damage. This gene functionally complements Chinese hamster irs1SF, a repair-deficient mutant that exhibits hypersensitivity to a number of different DNA-damaging agents and is chromosomally unstable. A rare microsatellite polymorphism in this gene is associated with cancer in patients of varying radiosensitivity. | DNA strand-pairing protein; Hydrolase | DNA repair; DNA recombination; Stress response | N/A |
| ***ZFHX1B*** | 9839 | 2q22 | Same as ZEB2 or zinc finger E-box binding homeobox 2. The SMADIP1 gene (also known as SIP1) is a member of the delta-EF1 (TCF8; MIM 189909)/Zfh1 family of 2-handed zinc finger/homeodomain proteins. SMADIP1 interacts with receptor-mediated, activated full-length SMADs (see MIM 605568). | KRAB box transcription factor | Biological process unclassified | N/A |
| ***ZIC3*** | 7547 | Xq26.2 | Zic family member 3 heterotaxy 1 (odd-paired homolog, Drosophila). This gene encodes a member of the ZIC family of C2H2-type zinc finger proteins. This nuclear protein probably functions as a transcription factor in early stages of left-right body axis formation. Mutations in this gene cause X-linked visceral heterotaxy, which includes congenital heart disease and left-right axis defects in organs. | Zinc finger transcription factor | mRNA transcription regulation; Embryogenesis; Neurogenesis | N/A |
| ***ZNF189*** | 7743 | 9q22-q31 | Zinc finger protein 189 | KRAB box transcription factor | mRNA transcription regulation; Cell proliferation and differentiation | N/A |

^a^ Underlined are genes on the X-chromosome that were not analyzed in the present study.

^b^ Gene ID from NCBI Entrez Gene (<http://www.ncbi.nlm.nih.gov/sites/entrez>).

^c^ From the PANTHER database ([http://www.pantherdb.org](http://www.pantherdb.org/)). N/A, not available.
