# Supplementary material for: Genetic Determinants of Facial Clefting: Analysis of 357 Candidate Genes Using Two National Cleft Studies from Scandinavia
Source: PLoS One. 2009 Apr 29;4(4):e5385. doi: 10.1371/journal.pone.0005385 (PMC2671138; doi:10.1371/journal.pone.0005385)
Supplement: Table S2 — Genes and SNPs. (1.78 MB DOC) [file pone.0005385.s002.doc]

**Table S2.** Genes and SNPs

| **Gene Name** ^a^ | **Chromosome** | **SNP Name** | **Physical location (bp)** ^b^ |
| --- | --- | --- | --- |
| ***ABCA1*** | 9q31.1 | rs2740481 | 104635972 |
|  |  | rs2230808 | 104642359 |
|  |  | rs2740479 | 104642992 |
|  |  | rs2254884 | 104661304 |
|  |  | rs4149313 | 104666308 |
|  |  | rs2487054 | 104684277 |
|  |  | rs2472384 | 104698236 |
|  |  | rs2230806 | 104700422 |
|  |  | rs4149272 | 104721842 |
|  |  | rs3858075 | 104724385 |
| ***ACTN1*** | 14q24.1-q24.2 | rs2073307 | 68412003 |
|  |  | rs3784132 | 68432440 |
|  |  | rs2268973 | 68493775 |
| ***ADAM17*** | 2p25 | rs17524594 | 9580614 |
|  |  | rs6705408 | 9580829 |
|  |  | rs1056204 | 9597689 |
|  |  | rs4499396 | 9610208 |
|  |  | rs4278906 | 9612187 |
|  |  | rs16867049 | 9618528 |
|  |  | rs10495565 | 9637038 |
| ***ADH1A*** | 4q21-q23 | rs1230025 | 100543554 |
|  |  | rs13123099 | 100551695 |
|  |  | rs3819197 | 100557687 |
| ***ADH1B*** | 4q21-q23 | rs1042026 | 100585644 |
|  |  | rs1229984 | 100596497 |
|  |  | rs1353621 | 100598753 |
|  |  | rs1159918 | 100600187 |
| ***ADH1C*** | 4q21-q23 | rs698 | 100617967 |
|  |  | rs1693482 | 100621143 |
|  |  | rs2241894 | 100623311 |
|  |  | rs3133158 | 100627790 |
| ***ADH4*** | 4q21-q24 | rs2602880 | 100397831 |
|  |  | rs3828541 | 100399440 |
|  |  | rs1042364 | 100402752 |
|  |  | rs1126671 | 100405592 |
|  |  | rs3762894 | 100423262 |
| ***ADH5*** | 4q21-q25 | rs1230155 | 100346437 |
|  |  | rs7684986 | 100348854 |
|  |  | rs1154414 | 100357314 |
|  |  | rs1154400 | 100367188 |
| ***AHCY*** | 20cen-q13.1 | rs819142 | 32324668 |
|  |  | rs819133 | 32333975 |
|  |  | rs6088466 | 32377195 |
| ***AHR*** | 7p15 | rs2282886 | 17116645 |
|  |  | rs3802083 | 17122147 |
|  |  | rs2282883 | 17129587 |
|  |  | rs2066853 | 17152350 |
| ***AIP*** | 11q13 | rs1638588 | 66956282 |
|  |  | rs3741168 | 66991927 |
|  |  | rs4084113 | 67013613 |
| ***ALDH1A1*** | 9q21.13 | rs348472 | 72750614 |
|  |  | rs10781106 | 72752476 |
|  |  | rs348458 | 72759442 |
|  |  | rs2303317 | 72771496 |
|  |  | rs2017362 | 72773945 |
|  |  | rs348462 | 72776723 |
|  |  | rs348463 | 72777166 |
|  |  | rs1330286 | 72782507 |
| ***ALK3*** | 10q22.3 | rs4934262 | 88536108 |
|  |  | rs4933411 | 88548451 |
|  |  | rs4934268 | 88561319 |
|  |  | rs11202221 | 88592294 |
|  |  | rs2354354 | 88596931 |
|  |  | rs2883420 | 88601102 |
|  |  | rs10749542 | 88621608 |
| ***ALK6*** | 4q22-q24 | rs925283 | 96063515 |
|  |  | rs6847607 | 96107201 |
|  |  | rs3775010 | 96178597 |
|  |  | rs7673752 | 96234858 |
|  |  | rs9997720 | 96320970 |
|  |  | rs6838546 | 96379928 |
| ***ALX3*** | 1p21-p13 | rs12749726 | 110316122 |
|  |  | rs3820664 | 110318123 |
|  |  | rs3754443 | 110321330 |
|  |  | rs3754439 | 110327136 |
|  |  | rs1466788 | 110330772 |
| ***ALX4*** | 11p11.2 | rs897004 | 44240501 |
|  |  | rs3802805 | 44243142 |
|  |  | rs3824915 | 44288085 |
| ***AMT*** | 3p21.2-p21.1 | rs4855877 | 49423531 |
|  |  | rs6997 | 49428838 |
|  |  | rs10640 | 49429281 |
| ***APE1*** | 14q11.2-q12 | rs2275008 | 19986089 |
|  |  | rs938883 | 19986798 |
|  |  | rs1320150 | 19989883 |
|  |  | rs3136820 | 19994994 |
| ***APOA1*** | 11q23-q24 | rs5076 | 116212400 |
| ***APOA1BP*** | 1q22-q21.2 | rs3795732 | 153377713 |
|  |  | rs618923 | 116159369 |
|  |  | rs619054 | 116166023 |
|  |  | rs1729410 | 116170871 |
| ***APOB*** | 2p24-p23 | rs12713450 | 21136495 |
|  |  | rs1042034 | 21136933 |
|  |  | rs12713559 | 21140720 |
|  |  | rs676210 | 21143176 |
|  |  | rs12713675 | 21144025 |
|  |  | rs12713681 | 21144497 |
|  |  | rs12713843 | 21150019 |
|  |  | rs12714097 | 21157541 |
|  |  | rs12691202 | 21161368 |
|  |  | rs679899 | 21162566 |
|  |  | rs12714214 | 21163019 |
|  |  | rs12714225 | 21167007 |
| ***APOC2*** | 19q13.2 | rs2288911 | 50141124 |
|  |  | rs5126 | 50144269 |
|  |  | rs3760626 | 50148945 |
| ***APOE*** | 19q13.2 | rs2075650 | 50087459 |
|  |  | rs8106922 | 50093506 |
|  |  | rs405509 | 50100676 |
|  |  | rs439401 | 50106291 |
| ***ARNT*** | 1q21.3 | rs2228099 | 147621962 |
|  |  | rs7532045 | 147634920 |
|  |  | rs11204735 | 147654740 |
|  |  | rs3738480 | 147803898 |
| ***ARNT2*** | 15q24 | rs4238518 | 78513794 |
|  |  | rs2305145 | 78522092 |
|  |  | rs4331301 | 78585923 |
|  |  | rs4778604 | 78624874 |
|  |  | rs4301984 | 78674486 |
| ***ARVCF*** | 22q11.21 | rs165815 | 18334027 |
|  |  | rs2240717 | 18343660 |
|  |  | rs1990277 | 18345056 |
|  |  | rs2073746 | 18353112 |
|  |  | rs2238794 | 18373172 |
| ***ARX*** | Xp22.1-p21.3 | rs2285563 | 24786650 |
|  |  | rs7063687 | 24789943 |
| ***ATIC*** | 2q35 | rs13417729 | 216011331 |
|  |  | rs3772078 | 216015351 |
|  |  | rs2372536 | 216015526 |
|  |  | rs2030774 | 216034298 |
| ***ATR*** | 3q22-q24 | rs9816736 | 143655478 |
|  |  | rs3922730 | 143685555 |
|  |  | rs7630115 | 143693499 |
|  |  | rs4256152 | 143702240 |
|  |  | rs10804682 | 143717232 |
|  |  | rs2227928 | 143764310 |
| ***ATRX*** | Xq13.1-q21.1 | rs4826225 | 76599417 |
| ***BAMBI*** | 10p12.3-p11.2 | rs604352 | 28992658 |
|  |  | rs10826490 | 29003271 |
|  |  | rs675558 | 29008312 |
|  |  | rs655766 | 29017016 |
| ***BARX1*** | 9q12 | rs2095778 | 93784189 |
|  |  | rs1933679 | 93788577 |
|  |  | rs1933683 | 93792326 |
|  |  | rs7872123 | 93795713 |
| ***BARX2*** | 11q25 | rs2323587 | 128757778 |
|  |  | rs4937426 | 128779244 |
|  |  | rs1353111 | 128780953 |
|  |  | rs6590385 | 128798776 |
|  |  | rs11828252 | 128805081 |
| ***BCL3*** | 19q13.1-q13.2 | rs2965174 | 49936855 |
|  |  | rs8103315 | 49946008 |
|  |  | rs1046881 | 49954765 |
| ***BCOR*** | Xp11.4 | rs6609050 | 39671943 |
|  |  | rs6609051 | 39694449 |
|  |  | rs12687359 | 39701849 |
| ***BHMT*** | 5q13.1-q15 | rs567754 | 78452172 |
|  |  | rs3733890 | 78457715 |
|  |  | rs585800 | 78462964 |
| ***BHMT2*** | 5q13 | rs542721 | 78394188 |
|  |  | rs642431 | 78400443 |
|  |  | rs10944 | 78421601 |
| ***BMP10*** | 2p13.3 | rs2871760 | 68992289 |
|  |  | rs2312078 | 69006803 |
|  |  | rs4500981 | 69015026 |
| ***BMP2*** | 20p12.3 | rs1980499 | 6694498 |
|  |  | rs1005464 | 6704148 |
|  |  | rs235768 | 6707115 |
|  |  | rs3178250 | 6708201 |
| ***BMP4*** | 14q22.2 | rs2147105 | 53475815 |
|  |  | rs17563 | 53487272 |
|  |  | rs2071047 | 53488161 |
|  |  | rs762642 | 53492803 |
| ***BMP6*** | 6p24-p23 | rs270413 | 7694642 |
|  |  | rs270404 | 7702373 |
|  |  | rs1358893 | 7731412 |
|  |  | rs6938135 | 7743422 |
|  |  | rs267802 | 7760518 |
|  |  | rs267190 | 7787120 |
|  |  | rs752751 | 7816613 |
| ***BMPR2*** | 2q33-q34 | rs6435149 | 203096773 |
|  |  | rs6747299 | 203110663 |
|  |  | rs12693968 | 203128133 |
|  |  | rs4675278 | 203160078 |
|  |  | rs12621870 | 203183720 |
| ***CASR*** | 3q21-q24 | rs4678029 | 123391124 |
|  |  | rs1814740 | 123401181 |
|  |  | rs9866419 | 123422899 |
|  |  | rs4300957 | 123432826 |
|  |  | rs7644390 | 123472918 |
|  |  | rs1042636 | 123486459 |
| ***CBS*** | 21q22.3 | rs4920037 | 43354960 |
|  |  | rs234705 | 43356841 |
|  |  | rs234709 | 43360033 |
| ***CCDC6*** | 10q21 | rs1053266 | 61222698 |
|  |  | rs1053265 | 61222780 |
| ***CCR1*** | 3p21 | rs3136663 | 46221231 |
| ***CCR6*** | 6q27 | rs1556413 | 167495154 |
|  |  | rs3093023 | 167504701 |
| ***CCT3*** | 1q23 | rs10908501 | 153098586 |
|  |  | rs2296374 | 153100476 |
|  |  | rs2296375 | 153122445 |
| ***CDH1*** | 16q22.1 | rs16260 | 67328535 |
|  |  | rs1078621 | 67336497 |
|  |  | rs4076177 | 67381509 |
|  |  | rs2010724 | 67389915 |
| ***CDH2*** | 18q11.2 | rs11083241 | 23805959 |
|  |  | rs6508522 | 23811871 |
|  |  | rs8092870 | 23816829 |
|  |  | rs597591 | 23836287 |
|  |  | rs694943 | 23880301 |
| ***CDKN1C*** | 11p15.5 | rs4930026 | 2856994 |
|  |  | rs452338 | 2864771 |
|  |  | rs384037 | 2869129 |
|  |  | rs400643 | 2869522 |
| ***CDX4*** | Xq13.2 | rs1554917 | 72455183 |
|  |  | rs2812027 | 72458216 |
| ***CEAL1*** | 19q13.31 | rs2289494 | 49878805 |
|  |  | rs2289495 | 49879142 |
|  |  | rs8103947 | 49882850 |
| ***CETP*** | 16q21 | rs173539 | 55545545 |
|  |  | rs5882 | 55573593 |
|  |  | rs289741 | 55574975 |
|  |  | rs289747 | 55581439 |
| ***CFC1*** | 2q21.1 | rs6715024 | 130986927 |
|  |  | rs1470041 | 130990005 |
| ***CHD7*** | 8q12.2 | rs2068096 | 61928114 |
|  |  | rs2272727 | 61931749 |
| ***CHES1*** | 14q24.3-q32.11 | rs722479 | 88711091 |
|  |  | rs2147100 | 88721925 |
|  |  | rs4899971 | 88772725 |
|  |  | rs10498619 | 88784816 |
|  |  | rs10484026 | 88879524 |
|  |  | rs2241122 | 88894569 |
|  |  | rs243175 | 89121344 |
|  |  | rs11622292 | 89136080 |
| ***CHL1*** | 3p26.1 | rs4684331 | 253366 |
|  |  | rs7640724 | 258039 |
|  |  | rs331891 | 313146 |
|  |  | rs2272522 | 336508 |
|  |  | rs13060847 | 366100 |
|  |  | rs3773384 | 391441 |
| ***CHRNA4*** | 20q13.2-q13.3 | rs4522666 | 61444924 |
|  |  | rs2236196 | 61448000 |
|  |  | rs3787137 | 61449544 |
|  |  | rs1044396 | 61451578 |
|  |  | rs2229959 | 61451998 |
|  |  | rs2273504 | 61458505 |
|  |  | rs755203 | 61464708 |
| ***CKM*** | 19q13.2-q13.3 | rs8111989 | 50501048 |
|  |  | rs7260463 | 50506700 |
|  |  | rs1133190 | 50510675 |
| ***CLPTM1*** | 19q13.2-q13.3 | rs11668758 | 50166520 |
|  |  | rs2075620 | 50171877 |
|  |  | rs8111069 | 50175278 |
| ***COL11A1*** | 1p21 | rs2229783 | 103064472 |
|  |  | rs1676486 | 103066159 |
|  |  | rs1085 | 103068189 |
|  |  | rs1625969 | 103080860 |
|  |  | rs3753841 | 103091939 |
|  |  | rs2045819 | 103112335 |
|  |  | rs2622848 | 103133024 |
|  |  | rs6672647 | 103136824 |
| ***COL11A2*** | 6p21.3 | rs926421 | 33216013 |
|  |  | rs7750683 | 33218812 |
|  |  | rs1003979 | 33222149 |
|  |  | rs3129207 | 33233290 |
|  |  | rs2235498 | 33238408 |
|  |  | rs2855442 | 33245381 |
| ***COL2A1*** | 12q13.11 | rs6823 | 46648679 |
|  |  | rs2070739 | 46654243 |
|  |  | rs1793953 | 46679793 |
|  |  | rs1793931 | 46682772 |
|  |  | rs1859444 | 46685670 |
| ***CORS26*** | 5p13-p12 | rs253202 | 34050931 |
|  |  | rs6546 | 34055787 |
|  |  | rs299602 | 34061668 |
| ***CRABP1*** | 15q24 | rs8027394 | 76404968 |
|  |  | rs7178402 | 76412612 |
|  |  | rs11072753 | 76414086 |
| ***CRELD1*** | 3p25.3 | rs2302786 | 9954660 |
|  |  | rs3774207 | 9960656 |
| ***CTH*** | 1p31.1 | rs681475 | 70592738 |
|  |  | rs1145920 | 70595861 |
|  |  | rs663649 | 70609204 |
|  |  | rs515064 | 70616089 |
|  |  | rs1021737 | 70616821 |
| ***CTNNB1*** | 3p22.1 | rs4533622 | 41217342 |
|  |  | rs6776881 | 41225485 |
|  |  | rs1798802 | 41236983 |
|  |  | rs3774371 | 41251170 |
| ***CUX2*** | 12q24.11 | rs10774613 | 110008885 |
|  |  | rs3809283 | 110170474 |
|  |  | rs1265566 | 110179096 |
|  |  | rs3847953 | 110228184 |
| ***CX43*** | 6q21-q23.2 | rs12197797 | 121805662 |
|  |  | rs11961755 | 121807985 |
| ***CXORF5*** | Xp22.2-p22.3 | rs2285635 | 13512552 |
|  |  | rs2283707 | 13543591 |
| ***CYP1A1*** | 15q24.1 | rs1048943 | 72800038 |
|  |  | rs4646421 | 72803245 |
|  |  | rs2606345 | 72804229 |
|  |  | rs4886406 | 72844256 |
| ***CYP1A2*** | 15q24.1 | rs7495739 | 72972723 |
|  |  | rs2470890 | 72834479 |
|  |  | rs11854147 | 72839824 |
| ***CYP1B1*** | 2p22.2 | rs163078 | 38198596 |
|  |  | rs2256327 | 38203355 |
|  |  | rs162549 | 38207107 |
|  |  | rs10916 | 38208821 |
|  |  | rs1056836 | 38209854 |
|  |  | rs162556 | 38218105 |
|  |  | rs2447752 | 38225374 |
| ***CYP2D6*** | 22q13.2 | rs5758589 | 40842880 |
|  |  | rs1058172 | 40848026 |
|  |  | rs1058167 | 40862527 |
|  |  | rs2743467 | 40873378 |
|  |  | rs873833 | 22752432 |
| ***CYP2E1*** | 10q26.3 | rs1329151 | 135123274 |
|  |  | rs9418990 | 135226847 |
|  |  | rs743535 | 135238248 |
|  |  | rs2515642 | 135240894 |
|  |  | rs1536826 | 135246120 |
| ***CYP3A7*** | 7q22.1 | rs651430 | 99074494 |
|  |  | rs1025576 | 99118275 |
| ***DHAND*** | 4q34.1 | rs2119788 | 174822119 |
|  |  | rs2877766 | 174830415 |
| ***DHCR24*** | 1p33-p31.1 | rs683737 | 55026163 |
|  |  | rs11206455 | 55037815 |
|  |  | rs642737 | 55045474 |
| ***DHCR7*** | 11q13.2-q13.5 | rs1792282 | 70812814 |
|  |  | rs1790345 | 70823589 |
|  |  | rs3750997 | 70836489 |
| ***DHFR*** | 5q11.2-q13.2 | rs2618372 | 79961366 |
|  |  | rs1643638 | 79966012 |
|  |  | rs380691 | 79987790 |
| ***DKK1*** | 10q11.2 | rs1528877 | 53741348 |
|  |  | rs2288335 | 53749060 |
|  |  | rs12780421 | 53757938 |
| ***DLX1*** | 2q31.1 | rs788172 | 172778945 |
|  |  | rs813720 | 172780248 |
|  |  | rs1486957 | 172838880 |
| ***DLX2*** | 2q31.1 | rs4972740 | 172933950 |
|  |  | rs4519482 | 172788866 |
| ***DLX3*** | 17q21 | rs3891034 | 45425224 |
|  |  | rs2303466 | 45425877 |
|  |  | rs2278163 | 45427425 |
| ***DLX5*** | 7q21.3 | rs1207730 | 96289980 |
|  |  | rs1207735 | 96300377 |
|  |  | rs6960249 | 96304783 |
|  |  | rs1207721 | 96319075 |
| ***DLX6*** | 7q21.3 | rs1004278 | 96282696 |
|  |  | rs3213654 | 96284019 |
|  |  | rs2272280 | 96284781 |
| ***DLX7*** | 17q21.33 | rs11079884 | 45431434 |
|  |  | rs890397 | 45458934 |
|  |  | rs919089 | 45402420 |
|  |  | rs4793624 | 45414776 |
| ***DMGDH*** | 5q14.1 | rs250513 | 78337952 |
|  |  | rs479405 | 78338615 |
|  |  | rs642013 | 78343021 |
|  |  | rs2034899 | 78351944 |
|  |  | rs1805074 | 78360108 |
|  |  | rs248386 | 78365983 |
|  |  | rs185077 | 78367579 |
|  |  | rs532964 | 78376042 |
| ***DSP*** | 6p24 | rs1998323 | 7491403 |
|  |  | rs2744372 | 7499465 |
|  |  | rs2076297 | 7513353 |
|  |  | rs2076299 | 7525957 |
|  |  | rs6929069 | 7526635 |
| ***DTDST*** | 5q33.1 | rs245056 | 149321231 |
|  |  | rs245055 | 149321472 |
|  |  | rs245081 | 149336268 |
|  |  | rs17110742 | 149337253 |
|  |  | rs3776070 | 149341414 |
| ***DVL1*** | 1p36 | rs3737720 | 1281723 |
|  |  | rs3737717 | 1282007 |
| ***EDN1*** | 6p24.1 | rs6931867 | 12385608 |
|  |  | rs10807242 | 12387672 |
|  |  | rs3087459 | 12397625 |
|  |  | rs1630736 | 12403973 |
|  |  | rs5370 | 12404241 |
| ***EFNB1*** | Xq12 | rs421069 | 67833003 |
|  |  | rs877818 | 67836528 |
| ***EGF*** | 4q25 | rs881878 | 111193652 |
|  |  | rs3796944 | 111231530 |
|  |  | rs11568943 | 111240725 |
|  |  | rs1860129 | 111243947 |
|  |  | rs2237051 | 111258802 |
|  |  | rs720419 | 111303570 |
| ***EGFR*** | 7p11.2 | rs759171 | 54860421 |
|  |  | rs763317 | 54869406 |
|  |  | rs723527 | 54909081 |
|  |  | rs4947971 | 54935204 |
|  |  | rs2075110 | 54993368 |
|  |  | rs845552 | 55019716 |
| ***EGR3*** | 8p23-p21 | rs10099846 | 22590123 |
|  |  | rs10104039 | 22594329 |
|  |  | rs1996147 | 22600103 |
|  |  | rs1008949 | 22609566 |
| ***EMX2*** | 10q26.1 | rs242956 | 119287469 |
|  |  | rs385209 | 119294046 |
|  |  | rs2286628 | 119311304 |
| ***EPHB2*** | 1p36.12 | rs2986657 | 22785973 |
|  |  | rs4655093 | 22800666 |
|  |  | rs4503323 | 22899785 |
|  |  | rs4654821 | 22905578 |
|  |  | rs309476 | 22958046 |
|  |  | rs309500 | 22978906 |
| ***EPHB3*** | 3q27.1 | rs9854599 | 185753944 |
|  |  | rs4132006 | 185764358 |
|  |  | rs4072650 | 185778530 |
|  |  | rs7652033 | 185781869 |
|  |  | rs11711863 | 185808657 |
| ***EPHX1*** | 1q42.12 | rs1223246 | 222400097 |
|  |  | rs14868 | 222414522 |
|  |  | rs3753658 | 222319421 |
|  |  | rs1051740 | 222326368 |
|  |  | rs2234922 | 222333141 |
| ***EPS15*** | 1p31-p32 | rs7308 | 51533011 |
|  |  | rs17567 | 51538942 |
|  |  | rs2405699 | 51556799 |
|  |  | rs1065754 | 51585972 |
|  |  | rs6668872 | 51669991 |
| ***ERCC4*** | 16p13.3-p13.11 | rs11649492 | 13916919 |
|  |  | rs3136079 | 13926738 |
|  |  | rs2020955 | 13946160 |
|  |  | rs3743538 | 13950189 |
| ***ESR1*** | 6q25.1 | rs10484922 | 152224431 |
|  |  | rs827423 | 152248311 |
|  |  | rs3853250 | 152252014 |
|  |  | rs2347867 | 152321964 |
|  |  | rs988328 | 152333264 |
|  |  | rs3020411 | 152435877 |
|  |  | rs3020368 | 152463304 |
|  |  | rs2982896 | 152491607 |
| ***ESR2*** | 14q | rs1256061 | 63773346 |
|  |  | rs8017441 | 63785547 |
|  |  | rs7154455 | 63806413 |
|  |  | rs1256030 | 63816923 |
|  |  | rs6573553 | 63824114 |
| ***ESRRB*** | 14q24.3 | rs2361330 | 75911208 |
|  |  | rs12588179 | 75915891 |
|  |  | rs10135736 | 75945199 |
|  |  | rs745011 | 75987028 |
|  |  | rs2921451 | 76009673 |
| ***ETV5*** | 3q28 | rs9820527 | 187235469 |
|  |  | rs9818739 | 187240446 |
|  |  | rs6780296 | 187246268 |
|  |  | rs7433760 | 187284330 |
|  |  | rs1516728 | 187312593 |
| ***EVC*** | 4p16 | rs4513510 | 5839846 |
|  |  | rs1871584 | 5863792 |
|  |  | rs735172 | 5876941 |
|  |  | rs1383180 | 5903514 |
| ***EVI1*** | 3q24-q28 | rs1479404 | 170271394 |
|  |  | rs6806825 | 170272407 |
|  |  | rs10513659 | 170292018 |
|  |  | rs17466625 | 170293576 |
|  |  | rs4955638 | 170302075 |
| ***EYA1*** | 8q13.3 | rs900109 | 72275703 |
|  |  | rs13262002 | 72299815 |
|  |  | rs1900079 | 72328807 |
|  |  | rs6472575 | 72345305 |
|  |  | rs10106252 | 72366884 |
|  |  | rs10104134 | 72401189 |
|  |  | rs1445403 | 72422808 |
| ***F13A1*** | 6p25.3-p24.3 | rs5988 | 6097136 |
|  |  | rs5982 | 6119865 |
|  |  | rs3024436 | 6141603 |
|  |  | rs2230848 | 6167325 |
|  |  | rs1781785 | 6174347 |
|  |  | rs7766109 | 6201832 |
|  |  | rs3851514 | 6219569 |
|  |  | rs5985 | 6263794 |
| ***FGF1*** | 5q31 | rs34012 | 141975961 |
|  |  | rs249916 | 141988572 |
|  |  | rs7722035 | 142036157 |
|  |  | rs11167790 | 142048550 |
| ***FGF10*** | 5p12 | rs2290070 | 44341066 |
|  |  | rs1448035 | 44347525 |
|  |  | rs980510 | 44354289 |
|  |  | rs900379 | 44405413 |
|  |  | rs723166 | 44431772 |
| ***FGF12*** | 3q28 | rs11717284 | 193407902 |
|  |  | rs6790664 | 193421939 |
|  |  | rs1464942 | 193569346 |
|  |  | rs1525911 | 193621434 |
|  |  | rs2701595 | 193793922 |
|  |  | rs12106855 | 193829112 |
|  |  | rs1875735 | 193842535 |
| ***FGF2*** | 4q26-q27 | rs3804150 | 124116783 |
|  |  | rs308428 | 124122113 |
|  |  | rs3789138 | 124142326 |
|  |  | rs308388 | 124144684 |
|  |  | rs7694627 | 124146100 |
|  |  | rs12506776 | 124160975 |
|  |  | rs1476214 | 124170614 |
| ***FGF4*** | 11q13.3 | rs713217 | 69288251 |
|  |  | rs3897660 | 69306312 |
| ***FGF5*** | 4q21 | rs3796606 | 81551269 |
|  |  | rs3733336 | 81565142 |
| ***FGF7*** | 15q15-q21.1 | rs10519225 | 47508070 |
|  |  | rs12592277 | 47516051 |
|  |  | rs4338740 | 47522589 |
|  |  | rs10519230 | 47535657 |
|  |  | rs7178101 | 47539986 |
|  |  | rs4480740 | 47543134 |
|  |  | rs2413959 | 47547717 |
| ***FGF8*** | 10q24.32 | rs10786646 | 103429092 |
|  |  | rs749694 | 103509774 |
|  |  | rs1008013 | 103538856 |
|  |  | rs874885 | 103589315 |
|  |  | rs10786650 | 103652779 |
| ***FGF9*** | 13q11-q12 | rs1886861 | 21141370 |
|  |  | rs9550757 | 21147975 |
|  |  | rs2274296 | 21153301 |
|  |  | rs829220 | 21158835 |
|  |  | rs4770192 | 21167455 |
|  |  | rs10047718 | 21172442 |
| ***FGFBP1*** | 4p16-p15 | rs732245 | 15613354 |
|  |  | rs4698427 | 15623201 |
|  |  | rs12509859 | 15627000 |
| ***FGFR1*** | 8p12 | rs3925 | 38400815 |
|  |  | rs2288696 | 38405382 |
|  |  | rs2978073 | 38414699 |
|  |  | rs6987534 | 38418872 |
|  |  | rs7825208 | 38446444 |
|  |  | rs7830964 | 38469472 |
| ***FGFR2*** | 10q26.13 | rs1047057 | 123229102 |
|  |  | rs2912795 | 123240238 |
|  |  | rs755793 | 123300861 |
|  |  | rs2981430 | 123301688 |
|  |  | rs2912779 | 123327172 |
| ***FGFR3*** | 4p16.3 | rs6599400 | 1752256 |
|  |  | rs2301293 | 1769097 |
| ***FGFR4*** | 5q35.1-qter | rs640180 | 176445853 |
|  |  | rs1966265 | 176449237 |
|  |  | rs376618 | 176450403 |
|  |  | rs6556301 | 176460183 |
| ***FLNA*** | Xq28 | rs766419 | 153075508 |
|  |  | rs2070822 | 153100295 |
|  |  | rs2070825 | 153102779 |
|  |  | rs2070816 | 153116265 |
| ***FLNB*** | 3p14.3 | rs1658397 | 57993147 |
|  |  | rs2033739 | 58038859 |
|  |  | rs6445945 | 58064556 |
|  |  | rs1131356 | 58084202 |
|  |  | rs12632456 | 58093595 |
|  |  | rs2001972 | 58098289 |
| ***FOLH1*** | 11p11.2 | rs6485963 | 49115799 |
|  |  | rs11040270 | 49122246 |
|  |  | rs7113251 | 49143860 |
|  |  | rs202720 | 49148927 |
|  |  | rs10839236 | 49149243 |
|  |  | rs202680 | 49178461 |
|  |  | rs202676 | 49184196 |
| ***FOLR3*** | 11q13.4 | rs533207 | 71525934 |
|  |  | rs555306 | 71532141 |
|  |  | rs575341 | 71535021 |
| ***FOLRA*** | 11q13.4 | rs3016432 | 71574903 |
|  |  | rs11235468 | 71601190 |
| ***FOLRB*** | 11q13.4 | rs514933 | 71607855 |
|  |  | rs2298444 | 71610062 |
| ***FOXC2*** | 16q24.1 | rs1025057 | 85132227 |
|  |  | rs7189970 | 85147931 |
|  |  | rs4843165 | 85162542 |
| ***FOXE1*** | 9q22 | rs1912996 | 97677816 |
|  |  | rs894673 | 97691825 |
|  |  | rs3758251 | 97693255 |
|  |  | rs3758249 | 97693695 |
|  |  | rs907577 | 97694672 |
|  |  | rs1443435 | 97697138 |
|  |  | rs874004 | 97701673 |
|  |  | rs907581 | 97701744 |
| ***FOXF2*** | 6p25.3 | rs1711970 | 1331736 |
|  |  | rs732835 | 1337999 |
|  |  | rs2293783 | 1340042 |
| ***FOXH1*** | 8q24.3 | rs748193 | 145652923 |
|  |  | rs7002959 | 145656204 |
|  |  | rs750472 | 145672261 |
| ***FOXN1*** | 17q11-q12 | rs2071587 | 23875729 |
|  |  | rs548973 | 23880388 |
|  |  | rs637931 | 23887199 |
| ***FOXP2*** | 7q31 | rs10268637 | 113668208 |
|  |  | rs10486026 | 113674386 |
|  |  | rs10262103 | 113685795 |
|  |  | rs1229762 | 113812533 |
|  |  | rs936146 | 113888356 |
| ***FRAS1*** | 4q21.21 | rs4859905 | 79344336 |
|  |  | rs12504081 | 79562878 |
|  |  | rs13136648 | 79566642 |
|  |  | rs345513 | 79597242 |
|  |  | rs6835769 | 79641873 |
|  |  | rs1385134 | 79668227 |
|  |  | rs2035510 | 79719388 |
|  |  | rs7675266 | 79774965 |
| ***FSCN1*** | 7p22 | rs852481 | 5410966 |
|  |  | rs1640233 | 5416386 |
| ***FST*** | 5q11.2 | rs745321 | 52782003 |
|  |  | rs3797297 | 52813413 |
|  |  | rs1469101 | 52819165 |
| ***FTCD*** | 21q22.3 | rs1047209 | 46381237 |
|  |  | rs1980983 | 46383422 |
|  |  | rs9978174 | 46389340 |
|  |  | rs4819208 | 46396672 |
|  |  | rs2277820 | 46399800 |
| ***FTHFD*** | 3q21.2 | rs1127717 | 127308757 |
|  |  | rs3772430 | 127312239 |
|  |  | rs2290053 | 127315958 |
|  |  | rs2365004 | 127329819 |
|  |  | rs2276724 | 127337107 |
|  |  | rs2886059 | 127348464 |
|  |  | rs1823213 | 127361771 |
| ***FZD1*** | 7q21 | rs10953043 | 90533498 |
|  |  | rs3750145 | 90541482 |
|  |  | rs1052015 | 90542382 |
|  |  | rs2163085 | 90551785 |
| ***FZD10*** | 12q24.33 | rs10848026 | 129172589 |
|  |  | rs1046890 | 129174650 |
|  |  | rs1046893 | 129174896 |
| ***FZD2*** | 17q21.31 | rs3803869 | 39992580 |
|  |  | rs9897736 | 40005308 |
| ***FZD4*** | 11q14.2 | rs4144615 | 86330509 |
|  |  | rs10898563 | 86336861 |
|  |  | rs7925666 | 86347183 |
|  |  | rs11234891 | 86348490 |
| ***FZD7*** | 2q33.1 | rs2280509 | 202722874 |
|  |  | rs13034206 | 202726741 |
|  |  | rs7583130 | 202730594 |
|  |  | rs4673224 | 202741404 |
| ***FZD8*** | 10p11.21 | rs10827518 | 35952358 |
|  |  | rs1353 | 35967351 |
|  |  | rs10454470 | 35973484 |
| ***GABRB3*** | 15q12 | rs1432007 | 24361782 |
|  |  | rs1897356 | 24416628 |
|  |  | rs2194958 | 24458502 |
|  |  | rs878960 | 24480029 |
|  |  | rs11637930 | 24522927 |
|  |  | rs2059574 | 24548136 |
|  |  | rs4906908 | 24591175 |
| ***GAD1*** | 2q31 | rs1049731 | 171500657 |
|  |  | rs3828275 | 171508247 |
|  |  | rs2241164 | 171512066 |
|  |  | rs2058725 | 171515628 |
|  |  | rs769407 | 171519215 |
| ***GAD2*** | 10p11.23 | rs1330581 | 26568841 |
|  |  | rs1805398 | 26574815 |
|  |  | rs2368160 | 26580777 |
|  |  | rs2839678 | 26599576 |
| ***GART*** | 21q22.1 | rs7283354 | 33798940 |
|  |  | rs8971 | 33805488 |
|  |  | rs2154583 | 33810810 |
|  |  | rs2834234 | 33816493 |
|  |  | rs9984077 | 33818983 |
|  |  | rs4817579 | 33832090 |
| ***GDF1*** | 19p12 | rs726407 | 18855638 |
|  |  | rs12974421 | 18861152 |
|  |  | rs886853 | 18870470 |
|  |  | rs2074797 | 18872244 |
| ***GJB2*** | 13q11-q12 | rs3751385 | 19660956 |
|  |  | rs2274083 | 19661380 |
|  |  | rs2274084 | 19661642 |
| ***GLI2*** | 2q14.2 | rs2123634 | 121287308 |
|  |  | rs4848641 | 121319814 |
|  |  | rs3768699 | 121448334 |
|  |  | rs13427953 | 121448892 |
|  |  | rs12618388 | 121458329 |
|  |  | rs3099537 | 121458466 |
| ***GLI3*** | 7p14.1 | rs2079451 | 41777316 |
|  |  | rs10259802 | 41780504 |
|  |  | rs846271 | 41810077 |
|  |  | rs846268 | 41860630 |
|  |  | rs846266 | 41861462 |
|  |  | rs3801176 | 41911946 |
|  |  | rs3801209 | 41971755 |
| ***GNMT*** | 6p12 | rs2296805 | 43036736 |
|  |  | rs2274514 | 43042478 |
| ***GPC3*** | Xq26.1 | rs6634941 | 132737511 |
|  |  | rs2284125 | 132755576 |
|  |  | rs5977928 | 132767980 |
|  |  | rs1264380 | 132770856 |
| ***GPR51*** | 9q22.1-q22.3 | rs2304389 | 98148135 |
|  |  | rs1435252 | 98183146 |
|  |  | rs1537959 | 98252249 |
|  |  | rs2491397 | 98284717 |
|  |  | rs2779547 | 98322576 |
|  |  | rs2779562 | 98357236 |
|  |  | rs3750344 | 98419871 |
|  |  | rs1547272 | 98472310 |
|  |  | rs1930139 | 98509143 |
|  |  | rs509747 | 98549489 |
| ***GRLF1*** | 19q13.3 | rs311368 | 52138402 |
|  |  | rs311370 | 52138531 |
|  |  | rs311366 | 52158678 |
|  |  | rs311365 | 52162376 |
|  |  | rs7253105 | 52180731 |
|  |  | rs1052668 | 52199308 |
| ***GSTA4*** | 6p12.2 | rs367836 | 52951090 |
|  |  | rs316141 | 52954117 |
|  |  | rs316133 | 52955510 |
|  |  | rs316128 | 52957105 |
|  |  | rs3756980 | 52959938 |
| ***GSTM1*** | 1p13.3 | rs668413 | 109907986 |
|  |  | rs737497 | 109943634 |
|  |  | rs2071487 | 109945123 |
|  |  | rs1065411 | 109945180 |
|  |  | rs929166 | 109967216 |
| ***GSTM3*** | 1p13.3 | rs1927328 | 109987786 |
|  |  | rs1537236 | 109991014 |
|  |  | rs7483 | 109991743 |
|  |  | rs1332018 | 109995014 |
| ***GSTP1*** | 11q13.2 | rs638140 | 67090434 |
|  |  | rs6591252 | 67102153 |
|  |  | rs947894 | 67109265 |
|  |  | rs7103632 | 67125019 |
| ***GSTT1*** | 22q11.23 | rs1002286 | 22581891 |
|  |  | rs4630 | 22700876 |
| ***HIC1*** | 17p13.3 | rs903158 | 1910768 |
|  |  | rs6503222 | 1924612 |
| ***HIF1A*** | 14q23.2 | rs810118 | 61206011 |
|  |  | rs1951795 | 61241179 |
|  |  | rs2301111 | 61269954 |
|  |  | rs2301113 | 61276301 |
|  |  | rs1319462 | 61288978 |
| ***HOGG1*** | 3p26.2 | rs3219008 | 9770543 |
|  |  | rs2072668 | 9773140 |
|  |  | rs1052133 | 9773773 |
| ***HOXA7*** | 7p15-p14 | rs2462907 | 26900471 |
|  |  | rs983186 | 26961899 |
|  |  | rs2301721 | 26969353 |
|  |  | rs10259620 | 26975529 |
|  |  | rs2237336 | 26981258 |
|  |  | rs6970537 | 26990599 |
|  |  | rs2285724 | 27000599 |
|  |  | rs2067087 | 27014900 |
|  |  | rs13243033 | 27021613 |
| ***HOXB6*** | 17q21.3 | rs872760 | 44023950 |
|  |  | rs9299 | 44024429 |
|  |  | rs15689 | 44039928 |
| ***HSP90*** | 14q32.33 | rs2298877 | 101617977 |
|  |  | rs11621560 | 101626496 |
|  |  | rs2224460 | 101633550 |
| ***HYAL1*** | 3p21.3-p21.2 | rs2624853 | 50103897 |
|  |  | rs1061474 | 50119955 |
|  |  | rs2071203 | 50286904 |
|  |  | rs2071205 | 50302433 |
| ***IDH1*** | 2q33.3 | rs17838654 | 208926818 |
|  |  | rs7565247 | 208929736 |
| ***IFNK*** | 9 | rs774354 | 27505967 |
|  |  | rs3849939 | 27515221 |
|  |  | rs4879540 | 27517479 |
| ***IKKA*** | 10q24-q25 | rs3750707 | 101927959 |
|  |  | rs3818411 | 101950956 |
|  |  | rs7903344 | 101967873 |
|  |  | rs663123 | 102004496 |
| ***IKKE*** | 1q32.1 | rs2274902 | 203034706 |
|  |  | rs1953090 | 203036542 |
|  |  | rs2184030 | 203055836 |
|  |  | rs3748022 | 203057860 |
|  |  | rs15672 | 203058353 |
| ***INHBA*** | 7p15-p13 | rs2237436 | 41504171 |
|  |  | rs2237435 | 41504293 |
|  |  | rs3801158 | 41512381 |
| ***INHBB*** | 2cen-q13 | rs4849864 | 120805800 |
|  |  | rs7589138 | 120812150 |
|  |  | rs11902591 | 120822233 |
|  |  | rs7576183 | 120830493 |
| ***IRF6*** | 1q32.2 | rs623360 | 206341260 |
|  |  | rs651141 | 206344243 |
|  |  | rs680331 | 206348267 |
|  |  | rs17015215 | 206352475 |
|  |  | rs674433 | 206353270 |
|  |  | rs2013162 | 206357079 |
|  |  | rs2236906 | 206359880 |
| ***IRF9*** | 14q11.2 | rs2236351 | 23685275 |
|  |  | rs2295979 | 23694581 |
|  |  | rs2236350 | 23700850 |
| ***ITGB3*** | 17q21.32 | rs2056131 | 42688742 |
|  |  | rs2015729 | 42709492 |
|  |  | rs2292699 | 42717294 |
|  |  | rs5921 | 42724600 |
| ***JAG1*** | 20p12.2 | rs8708 | 10566574 |
|  |  | rs3817996 | 10572046 |
|  |  | rs2235811 | 10592158 |
|  |  | rs6133987 | 10598354 |
| ***JAG2*** | 14q32.33 | rs2091918 | 104673664 |
|  |  | rs909236 | 104677201 |
|  |  | rs2072673 | 104695738 |
|  |  | rs2239284 | 104701534 |
| ***KCNJ2*** | 17q24.3 | rs236513 | 65681053 |
|  |  | rs173135 | 65683921 |
|  |  | rs236514 | 65685326 |
|  |  | rs643637 | 65687810 |
| ***KLHL4*** | Xq21.3 | rs6521948 | 86654375 |
|  |  | rs222084 | 86693389 |
|  |  | rs222108 | 86716255 |
|  |  | rs222110 | 86719817 |
| ***KREMEN1*** | 22q12.1 | rs134597 | 27795877 |
|  |  | rs20037 | 27802395 |
|  |  | rs713861 | 27810348 |
|  |  | rs10854812 | 27843696 |
| ***KRT14*** | 17q12-q21 | rs1008753 | 36977573 |
|  |  | rs4796674 | 36985185 |
|  |  | rs1840549 | 36989609 |
|  |  | rs4796588 | 37003449 |
| ***KRT18*** | 12q13 | rs2070875 | 51630585 |
|  |  | rs2363635 | 51640889 |
|  |  | rs727266 | 51642678 |
| ***L1CAM*** | Xq28 | rs5987171 | 152644258 |
|  |  | rs4646263 | 152661296 |
|  |  | rs3761531 | 152664622 |
|  |  | rs5945361 | 152667247 |
|  |  | rs12156710 | 152671638 |
| ***LCAT*** | 16q22.1 | rs2301246 | 66521704 |
|  |  | rs1109166 | 66534883 |
|  |  | rs2292318 | 66543207 |
| ***LDLR*** | 19p13.3 | rs5930 | 11085265 |
|  |  | rs688 | 11088602 |
|  |  | rs2738465 | 11103496 |
|  |  | rs1433099 | 11103658 |
| ***LEF1*** | 4q25 | rs1291490 | 109351616 |
|  |  | rs898518 | 109374428 |
|  |  | rs6533351 | 109380056 |
|  |  | rs749414 | 109385517 |
|  |  | rs922168 | 109415008 |
|  |  | rs922163 | 109423970 |
| ***LEFTY2*** | 1q42.1 | rs360096 | 222372273 |
|  |  | rs360104 | 222378339 |
|  |  | rs360076 | 222393071 |
|  |  | rs360074 | 222397885 |
|  |  | rs2273405 | 222430876 |
|  |  | rs3007716 | 222432352 |
|  |  | rs2180421 | 222467617 |
| ***LHX8*** | 1p31.1 | rs1007512 | 75283700 |
|  |  | rs941032 | 75334637 |
|  |  | rs6593568 | 75349940 |
|  |  | rs7545324 | 75374584 |
| ***LIMK1*** | 7q11.23 | rs810536 | 72948884 |
|  |  | rs2855726 | 72961765 |
|  |  | rs2269081 | 72964538 |
|  |  | rs150862 | 72968858 |
| ***LIPC*** | 15q21-q23 | rs3825776 | 56534122 |
|  |  | rs1968685 | 56555046 |
|  |  | rs1869137 | 56565704 |
|  |  | rs6078 | 56621285 |
|  |  | rs6083 | 56625302 |
|  |  | rs2242064 | 56626564 |
| ***LMX1B*** | 9q34 | rs1336980 | 126457409 |
|  |  | rs10819189 | 126459072 |
|  |  | rs4455975 | 126462753 |
|  |  | rs3829849 | 126470354 |
|  |  | rs4339739 | 126527685 |
| ***LOR*** | 1q21 | rs1329097 | 150037195 |
|  |  | rs873234 | 150040250 |
|  |  | rs4559442 | 150049297 |
| ***LPL*** | 8p22 | rs1534649 | 19843921 |
|  |  | rs10099160 | 19866095 |
|  |  | rs3866471 | 19868949 |
| ***MARK4*** | 19q13.3 | rs10445572 | 50459054 |
|  |  | rs2240672 | 50461415 |
|  |  | rs345409 | 50471475 |
|  |  | rs1568 | 50489932 |
| ***MAT1A*** | 10q22 | rs1556894 | 82014145 |
|  |  | rs2993763 | 82023574 |
|  |  | rs9285726 | 82025130 |
| ***MAT2A*** | 2p11.2 | rs6739015 | 85667015 |
|  |  | rs2028900 | 85679393 |
|  |  | rs2028898 | 85688928 |
|  |  | rs7568458 | 85699833 |
| ***MAT2B*** | 5q34-q35.1 | rs10515861 | 162863325 |
|  |  | rs4869087 | 162869803 |
|  |  | rs4869089 | 162876986 |
|  |  | rs729352 | 162881793 |
| ***MDCR*** | 17p13.3 | rs8068673 | 2449136 |
|  |  | rs1266475 | 2482070 |
|  |  | rs8081803 | 2511759 |
| ***MDR1*** | 7q21.1 | rs6949448 | 86786465 |
|  |  | rs2235039 | 86810505 |
|  |  | rs2235036 | 86819922 |
|  |  | rs2235013 | 86823277 |
|  |  | rs1922240 | 86828005 |
| ***MID1*** | Xp22 | rs741499 | 10265021 |
|  |  | rs2525073 | 10315185 |
|  |  | rs1965009 | 10316904 |
|  |  | rs7886985 | 10348745 |
|  |  | rs7053320 | 10350846 |
|  |  | rs6530404 | 10351406 |
| ***MKX*** | 10p12.1 | rs2492909 | 28003252 |
|  |  | rs1907390 | 28022532 |
|  |  | rs10829311 | 28042162 |
|  |  | rs10508728 | 28067169 |
| ***MMEL2*** | 1p36 | rs881640 | 2554366 |
|  |  | rs3748816 | 2558908 |
|  |  | rs10797437 | 2571168 |
| ***MMP13*** | 11q22.3 | rs11824755 | 102315174 |
|  |  | rs640198 | 102330301 |
|  |  | rs597315 | 102332994 |
|  |  | rs659383 | 102336301 |
|  |  | rs655316 | 102337574 |
|  |  | rs479963 | 102378938 |
| ***MMP14*** | 14q11-q12 | rs3811188 | 22368975 |
|  |  | rs1042703 | 22375888 |
|  |  | rs1042704 | 22382434 |
|  |  | rs4982695 | 22392453 |
| ***MMP2*** | 16q13-q21 | rs857403 | 54074209 |
|  |  | rs9302671 | 54079226 |
|  |  | rs2241145 | 54079701 |
|  |  | rs243849 | 54081206 |
|  |  | rs243842 | 54084923 |
|  |  | rs1992116 | 54085392 |
|  |  | rs243836 | 54091737 |
|  |  | rs243834 | 54094188 |
| ***MSC*** | 8q21 | rs1481851 | 72915206 |
|  |  | rs3779757 | 72916294 |
|  |  | rs7812453 | 72917594 |
|  |  | rs979451 | 72919997 |
| ***MSX1*** | 4p16.3-p16.1 | rs4075007 | 6641467 |
|  |  | rs732971 | 4961303 |
|  |  | rs3733397 | 4967474 |
|  |  | rs3821949 | 4978474 |
|  |  | rs1106514 | 4993998 |
| ***MSX2*** | 5q34-35 | rs1567202 | 174077866 |
|  |  | rs4868442 | 174085643 |
|  |  | rs4242182 | 174088774 |
|  |  | rs14459 | 174090317 |
|  |  | rs10057011 | 174093855 |
| ***MT1A*** | 16q13 | rs2270836 | 55225115 |
|  |  | rs4784701 | 55228168 |
|  |  | rs1001362 | 55232359 |
| ***MT4*** | 16q13 | rs2071005 | 55156405 |
|  |  | rs405536 | 55160739 |
|  |  | rs9302683 | 55167876 |
|  |  | rs1909749 | 55170203 |
| ***MTHFD1*** | 14q24 | rs3783731 | 63931140 |
|  |  | rs8003379 | 63943352 |
|  |  | rs1950902 | 63952133 |
|  |  | rs2236225 | 63978598 |
|  |  | rs2236224 | 63978904 |
|  |  | rs1256146 | 63990418 |
| ***MTHFD2*** | 2p13.1 | rs1667627 | 74340847 |
|  |  | rs702462 | 74356086 |
| ***MTHFR*** | 1p36.3 | rs4845877 | 11758569 |
|  |  | rs1476413 | 11786566 |
|  |  | rs1801131 | 11788742 |
|  |  | rs1801133 | 11790644 |
|  |  | rs7533315 | 11794949 |
|  |  | rs3737964 | 11801310 |
|  |  | rs12404124 | 11808135 |
| ***MTHFS*** | 15q25.1 | rs685487 | 77923184 |
|  |  | rs6495452 | 77951143 |
|  |  | rs2562744 | 77961443 |
| ***MTR*** | 1q43 | rs10925235 | 233288816 |
|  |  | rs6668344 | 233327367 |
|  |  | rs1805087 | 233374541 |
|  |  | rs16834521 | 233380610 |
| ***MTRR*** | 5p15.3-p15.2 | rs1801394 | 7923973 |
|  |  | rs1532268 | 7931179 |
|  |  | rs162031 | 7933287 |
|  |  | rs162036 | 7938959 |
|  |  | rs3776455 | 7949511 |
|  |  | rs10380 | 7950191 |
| ***MYL2*** | 12q23-q24.3 | rs2238149 | 109774649 |
|  |  | rs933296 | 109815568 |
|  |  | rs756823 | 109827570 |
| ***NAT1*** | 8p23.1-p21.3 | rs6998240 | 18105323 |
|  |  | rs10888150 | 18110406 |
|  |  | rs4298522 | 18112962 |
|  |  | rs7829368 | 18133537 |
|  |  | rs7015070 | 18136385 |
| ***NAT2*** | 8p23.1-21.3 | rs1390358 | 18297035 |
|  |  | rs1041983 | 18302075 |
|  |  | rs1801280 | 18302134 |
|  |  | rs1799929 | 18302274 |
|  |  | rs1208 | 18302596 |
|  |  | rs721398 | 18303585 |
|  |  | rs1495737 | 18309117 |
|  |  | rs1587145 | 18312696 |
| ***NBS1*** | 8q21 | rs9995 | 91015232 |
|  |  | rs2697679 | 91019056 |
|  |  | rs2735386 | 91020279 |
|  |  | rs6470523 | 91024346 |
|  |  | rs709816 | 91036887 |
|  |  | rs741778 | 91053979 |
|  |  | rs1805794 | 91059655 |
| ***NIPBL*** | 5p13.2 | rs158796 | 36914537 |
|  |  | rs292180 | 36986016 |
| ***NNMT*** | 11q23.1 | rs683271 | 113634355 |
|  |  | rs694539 | 113638629 |
|  |  | rs10891641 | 113644291 |
|  |  | rs2852432 | 113669214 |
|  |  | rs2852447 | 113684911 |
|  |  | rs4646337 | 113688519 |
|  |  | rs11569688 | 113688653 |
| ***NOTCH3*** | 19p13.2-p13.1 | rs1044055 | 15131805 |
|  |  | rs1044009 | 15132771 |
|  |  | rs2074621 | 15151412 |
| ***NQO1*** | 16q21.1 | rs10517 | 68301261 |
|  |  | rs1800566 | 68302646 |
|  |  | rs2917666 | 68321461 |
| ***NR3C1*** | 5q31 | rs6196 | 142641683 |
|  |  | rs258813 | 142654883 |
|  |  | rs4986593 | 142674039 |
|  |  | rs33388 | 142677488 |
|  |  | rs10482633 | 142730726 |
|  |  | rs2963156 | 142738689 |
|  |  | rs10482616 | 142761760 |
| ***NRXN2*** | 11q13 | rs2360873 | 64147733 |
|  |  | rs519090 | 64212816 |
|  |  | rs500531 | 64243595 |
| ***NSDHL*** | Xq28 | rs3788741 | 151673901 |
|  |  | rs4828729 | 151697563 |
|  |  | rs2071256 | 151707020 |
| ***OSR2*** | 8q22.2 | rs985794 | 100024750 |
|  |  | rs7824113 | 100059040 |
| ***PAX3*** | 2q35-q37; 2q35 | rs12620338 | 222890273 |
|  |  | rs7559271 | 222893791 |
|  |  | rs1978859 | 222907836 |
|  |  | rs10498134 | 222930485 |
|  |  | rs1367410 | 222931157 |
|  |  | rs930140 | 222973797 |
| ***PAX8*** | 2q12-q14 | rs11123170 | 113695171 |
|  |  | rs2863242 | 113705467 |
|  |  | rs6734610 | 113733259 |
|  |  | rs895417 | 113734820 |
| ***PAX9*** | 14q12-q13 | rs1955750 | 36156821 |
|  |  | rs1018466 | 36193001 |
|  |  | rs2073243 | 36199244 |
|  |  | rs1955734 | 36208379 |
|  |  | rs8004187 | 36210255 |
|  |  | rs1048200 | 36218136 |
| ***PDGFC*** | 4q32 | rs6837319 | 158055336 |
|  |  | rs4691379 | 158064509 |
|  |  | rs10517653 | 158091595 |
|  |  | rs2911940 | 158131918 |
|  |  | rs894588 | 158174748 |
| ***PDGFRA*** | 4q12 | rs7677751 | 54965388 |
|  |  | rs2303430 | 54965986 |
|  |  | rs7656613 | 54982771 |
|  |  | rs1547904 | 54987317 |
| ***PEX7*** | 6q21-q22.2 | rs927182 | 137203992 |
|  |  | rs2012700 | 137221080 |
|  |  | rs7750251 | 137245439 |
|  |  | rs1012515 | 137258540 |
| ***PHF8*** | Xp11.22 | rs6521788 | 53815855 |
|  |  | rs12115965 | 53827942 |
|  |  | rs7876951 | 53856370 |
|  |  | rs5960612 | 53949900 |
| ***PIGA*** | Xp22.1 | rs3434 | 15097271 |
|  |  | rs5978726 | 15101317 |
|  |  | rs6527487 | 15104280 |
| ***PIPOX*** | 17q11.2 | rs8067401 | 24399965 |
|  |  | rs7220662 | 24411302 |
| ***PITX1*** | 5q31 | rs28330 | 134380433 |
|  |  | rs31210 | 134388919 |
|  |  | rs888685 | 134393689 |
| ***PITX2*** | 4q25 | rs1448811 | 111868235 |
|  |  | rs2595110 | 111902927 |
|  |  | rs994978 | 111910002 |
| ***PKP1*** | 1q32 | rs832144 | 197994134 |
|  |  | rs713292 | 197997427 |
|  |  | rs706494 | 198002879 |
|  |  | rs1772836 | 198007438 |
|  |  | rs1543805 | 198024451 |
| ***POMT1*** | 9q34.1 | rs3887873 | 131414989 |
|  |  | rs3739494 | 131417042 |
|  |  | rs3739495 | 131428088 |
| ***PON1*** | 7q21.3 | rs854547 | 94568507 |
|  |  | rs8491 | 94570265 |
|  |  | rs854549 | 94571236 |
|  |  | rs2237582 | 94578851 |
|  |  | rs662 | 94582097 |
|  |  | rs3917498 | 94590906 |
|  |  | rs2074351 | 94592450 |
|  |  | rs854565 | 94592995 |
|  |  | rs2299261 | 94594314 |
|  |  | rs705382 | 94599872 |
| ***PQBP1*** | Xq13, Xp11.23 | rs4824733 | 48497341 |
|  |  | rs2016813 | 48508290 |
|  |  | rs741932 | 48515452 |
| ***PRDM16*** | 1p36.23-p3 | rs876740 | 3060770 |
|  |  | rs1798246 | 3104012 |
|  |  | rs2651929 | 3124510 |
|  |  | rs2500278 | 3249097 |
|  |  | rs2483274 | 3271255 |
|  |  | rs868688 | 3323964 |
|  |  | rs870124 | 3351515 |
|  |  | rs870171 | 3365961 |
| ***PRRX1*** | 1q24.2 | rs608930 | 167348964 |
|  |  | rs520525 | 167369991 |
|  |  | rs4656220 | 167380935 |
|  |  | rs520131 | 167385776 |
|  |  | rs513287 | 167395895 |
| ***PRRX2*** | 9q34.1 | rs10988467 | 129508481 |
|  |  | rs1867098 | 129528803 |
|  |  | rs920659 | 129541870 |
| ***PTCH1*** | 9q22.3 | rs2066829 | 95308944 |
|  |  | rs2297087 | 95322480 |
| ***PTCH2*** | 1p33-p34 | rs11584031 | 44948809 |
|  |  | rs6429550 | 44980842 |
| ***PTEN*** | 10q23.3 | rs1903858 | 89643666 |
|  |  | rs1234224 | 89665276 |
| ***PTPN11*** | 12q24 | rs11066301 | 111334092 |
|  |  | rs2301756 | 111353496 |
|  |  | rs11066320 | 111369135 |
|  |  | rs11066322 | 111385249 |
| ***PVR*** | 19q13.2 | rs7255066 | 49837943 |
|  |  | rs203709 | 49853406 |
| ***PVRL1*** | 11q23 | rs906827 | 119019339 |
|  |  | rs4938706 | 119044574 |
|  |  | rs4459318 | 119046988 |
|  |  | rs7122134 | 119052215 |
|  |  | rs7129848 | 119071438 |
|  |  | rs1876795 | 119101357 |
| ***PVRL2*** | 19q13.2-q13.4 | rs1871047 | 50043586 |
|  |  | rs4803763 | 50049131 |
|  |  | rs416041 | 50062694 |
|  |  | rs519113 | 50068124 |
|  |  | rs6859 | 50073874 |
| ***PVRL3*** | 3q13.13 | rs4682230 | 112279101 |
|  |  | rs9876350 | 112290656 |
|  |  | rs1477844 | 112312521 |
|  |  | rs7631446 | 112438758 |
| ***RAI1*** | 17p11.2 | rs7224617 | 17541304 |
|  |  | rs11656775 | 17595044 |
|  |  | rs11649804 | 17637480 |
|  |  | rs3818717 | 17647830 |
| ***RARA*** | 17q21 | rs2715553 | 35749846 |
|  |  | rs482284 | 35757767 |
|  |  | rs2715556 | 35790509 |
| ***RARB*** | 3p24 | rs1299407 | 25506928 |
|  |  | rs1997351 | 25513128 |
|  |  | rs1881703 | 25553556 |
|  |  | rs7610831 | 25577666 |
|  |  | rs1286735 | 25588905 |
|  |  | rs1730220 | 25593372 |
| ***RARG*** | 12q13 | rs1554753 | 51890005 |
|  |  | rs6580936 | 51904096 |
|  |  | rs10082776 | 51907978 |
| ***RECQL4*** | 8q24.3 | rs2101039 | 145701138 |
|  |  | rs756627 | 145708094 |
|  |  | rs2306386 | 145713687 |
|  |  | rs2721173 | 145715237 |
| ***RET*** | 10q11.2 | rs1799939 | 42930125 |
|  |  | rs1800861 | 42933849 |
|  |  | rs2075914 | 42943818 |
|  |  | rs2435355 | 42944839 |
| ***RFC1*** | 4p14-p13 | rs17754 | 39111874 |
|  |  | rs2381375 | 39136395 |
|  |  | rs3796517 | 39159519 |
| ***RIP3*** | 14q11.2 | rs4981504 | 23860976 |
|  |  | rs2295300 | 23874957 |
|  |  | rs3212254 | 23875303 |
|  |  | rs724165 | 23876069 |
| ***ROR1*** | 1p32-p31 | rs583102 | 64021323 |
|  |  | rs834473 | 64202414 |
|  |  | rs12409562 | 64262074 |
|  |  | rs2806537 | 64291178 |
|  |  | rs4565754 | 64336424 |
|  |  | rs7527017 | 64355298 |
| ***ROR2*** | 9q22.31 | rs3935053 | 91587669 |
|  |  | rs3935601 | 91588255 |
|  |  | rs4744098 | 91623837 |
|  |  | rs4595189 | 91626538 |
|  |  | rs7021744 | 91728815 |
|  |  | rs12554679 | 91740670 |
|  |  | rs7863167 | 91785053 |
| ***RPS19*** | 19q13.2 | rs12974044 | 47060469 |
|  |  | rs3786536 | 47064297 |
|  |  | rs1366610 | 47065138 |
|  |  | rs882520 | 47081120 |
| ***RUNX2*** | 6p21 | rs1406846 | 45508918 |
|  |  | rs910586 | 45518290 |
|  |  | rs2396441 | 45575743 |
|  |  | rs10485422 | 45597170 |
|  |  | rs6458445 | 45598196 |
|  |  | rs7748231 | 45609530 |
| ***RXRG*** | 1q22-q23 | rs2134095 | 162109210 |
|  |  | rs2651860 | 162112713 |
|  |  | rs4657437 | 162115279 |
|  |  | rs2281985 | 162117816 |
|  |  | rs157869 | 162120606 |
|  |  | rs517456 | 162127325 |
| ***RYK*** | 3q22 | rs10935104 | 135365759 |
|  |  | rs12635994 | 135404951 |
|  |  | rs9839609 | 135419467 |
|  |  | rs1131262 | 135424018 |
| ***SALL1*** | 16q12.1 | rs1007883 | 49720907 |
|  |  | rs2111125 | 49723392 |
|  |  | rs1965024 | 49731060 |
|  |  | rs1015438 | 49735018 |
| ***SALL2*** | 14q11.1-q12 | rs1124981 | 21060377 |
|  |  | rs1263810 | 21061466 |
|  |  | rs1263811 | 21063338 |
| ***SALL3*** | 18q23 | rs558191 | 74833209 |
|  |  | rs594821 | 74846577 |
|  |  | rs612829 | 74848306 |
|  |  | rs2472640 | 74852046 |
| ***SALL4*** | 20q13.13-q13.2 | rs6091375 | 49840037 |
|  |  | rs6021441 | 49844950 |
|  |  | rs6021460 | 49860267 |
| ***SARA1*** | 10q22.1 | rs15801 | 71580669 |
|  |  | rs2271690 | 71591597 |
|  |  | rs3858169 | 71599001 |
| ***SATB2*** | 2q33.1 | rs2881208 | 199963114 |
|  |  | rs2009622 | 199992742 |
|  |  | rs930616 | 200011421 |
|  |  | rs1014497 | 200066735 |
|  |  | rs4673339 | 200098882 |
|  |  | rs4675576 | 200104740 |
|  |  | rs4673346 | 200133486 |
| ***SC5DL*** | 11q23.3 | rs6589870 | 120659131 |
|  |  | rs1560409 | 120665270 |
|  |  | rs2060008 | 120685057 |
| ***SCD4*** | 4q21.3 | rs3733228 | 83939243 |
|  |  | rs3733227 | 83939390 |
|  |  | rs1880719 | 83974614 |
|  |  | rs1506609 | 83990152 |
|  |  | rs1358055 | 84005731 |
|  |  | rs4256218 | 84047858 |
|  |  | rs1980360 | 84051462 |
| ***SET*** | 9q34 | rs11542565 | 128531452 |
|  |  | rs3750330 | 128533674 |
|  |  | rs4836618 | 128535457 |
|  |  | rs4836619 | 128535588 |
| ***SHFM3*** | 10q24 | rs10883665 | 103364223 |
|  |  | rs11191069 | 103378119 |
|  |  | rs9651449 | 103380946 |
|  |  | rs3127230 | 103382493 |
|  |  | rs4244347 | 103442635 |
| ***SHH*** | 7q36 | rs1233560 | 155092914 |
|  |  | rs1233571 | 155093770 |
|  |  | rs288746 | 155106148 |
|  |  | rs288756 | 155123702 |
|  |  | rs1675570 | 155149976 |
| ***SHMT1*** | 17p11.2 | rs1979277 | 18172821 |
|  |  | rs2168781 | 18181471 |
|  |  | rs7207306 | 18188087 |
| ***SHMT2*** | 12q12-q14 | rs1800164 | 55891069 |
|  |  | rs7311958 | 55899692 |
| ***SIX3*** | 2p16-p21 | rs83995 | 45079221 |
|  |  | rs2673270 | 45082166 |
| ***SKI*** | 1p36.33 | rs263533 | 2195730 |
|  |  | rs260513 | 2212686 |
|  |  | rs7553178 | 2236917 |
| ***SLC7A11*** | 4q28-q32 | rs4504306 | 139450833 |
|  |  | rs4863771 | 139470941 |
|  |  | rs4602539 | 139482646 |
|  |  | rs7682497 | 139493004 |
|  |  | rs10519451 | 139514265 |
| ***SMAD1*** | 4q31 | rs11944685 | 146771872 |
|  |  | rs2289737 | 146776452 |
|  |  | rs714195 | 146803285 |
|  |  | rs11100883 | 146808575 |
| ***SMAD2*** | 18q21.1 | rs1792684 | 43623855 |
|  |  | rs1787186 | 43625507 |
|  |  | rs1792683 | 43648204 |
|  |  | rs1631576 | 43657481 |
|  |  | rs11082639 | 43680633 |
|  |  | rs4940086 | 43700305 |
| ***SMAD3*** | 15q22.33 | rs7162912 | 65148362 |
|  |  | rs10518705 | 65150337 |
|  |  | rs2118613 | 65165461 |
|  |  | rs4776886 | 65168612 |
|  |  | rs1465842 | 65172387 |
|  |  | rs991157 | 65206067 |
|  |  | rs731874 | 65233885 |
| ***SMAD4*** | 18q21.1 | rs12958604 | 46814323 |
|  |  | rs12968012 | 46821624 |
|  |  | rs10502913 | 46822269 |
|  |  | rs2229083 | 46829105 |
| ***SMS*** | Xp22.1 | rs2238958 | 21722800 |
|  |  | rs2040357 | 21738484 |
|  |  | rs5951678 | 21768641 |
| ***SNAI1*** | 20q13.1-q13.2 | rs6020157 | 48025165 |
|  |  | rs6020170 | 48031066 |
|  |  | rs6012791 | 48037380 |
|  |  | rs6067309 | 48043700 |
|  |  | rs1973947 | 48064792 |
| ***SNAI2*** | 8q11 | rs12155623 | 49974754 |
|  |  | rs2891682 | 49978377 |
|  |  | rs1023769 | 49985710 |
|  |  | rs1992375 | 50000397 |
|  |  | rs2582778 | 50001461 |
|  |  | rs13279837 | 50014091 |
| ***SNX3*** | 6q21 | rs588409 | 108664973 |
|  |  | rs3800223 | 108679188 |
| ***SOX1*** | 13q34 | rs3742223 | 111773197 |
|  |  | rs571564 | 111773792 |
| ***SOX5*** | 12p12.1 | rs10505893 | 23605703 |
|  |  | rs1871403 | 23676927 |
|  |  | rs2054472 | 23698902 |
|  |  | rs10771022 | 23789607 |
|  |  | rs4511370 | 23844721 |
|  |  | rs4469991 | 23970288 |
|  |  | rs2900528 | 24041708 |
|  |  | rs10771064 | 24208017 |
|  |  | rs1487658 | 24614333 |
| ***SOX9*** | 17q24.3-q25.1 | rs1477066 | 67618023 |
|  |  | rs7502198 | 67622102 |
|  |  | rs2229989 | 67630530 |
|  |  | rs9915657 | 67639131 |
|  |  | rs918080 | 67642487 |
| ***SP8*** | 7p21.2 | rs6944200 | 20574460 |
|  |  | rs2709748 | 20593010 |
|  |  | rs2529753 | 20612191 |
| ***SPAM1*** | 7q31.3 | rs2285996 | 123188157 |
| ***SPP1*** | 4q21-q25 | rs10516800 | 89248769 |
|  |  | rs6813526 | 89251414 |
|  |  | rs10516799 | 89260372 |
|  |  | rs4660 | 89261184 |
|  |  | rs9138 | 89261521 |
|  |  | rs4128340 | 89265315 |
| ***SPPL3*** | 12q24.31 | rs625228 | 119740986 |
|  |  | rs683091 | 119757464 |
|  |  | rs11065300 | 119761232 |
|  |  | rs610694 | 119767546 |
|  |  | rs3809313 | 119792687 |
| ***SPRY2*** | 13q31.1 | rs11911 | 79808852 |
|  |  | rs504122 | 79809526 |
| ***SPTLC1*** | 9q22.2 | rs6479400 | 91884861 |
|  |  | rs10820939 | 91930613 |
| ***STAT3*** | 17q21.31 | rs1064116 | 37728870 |
|  |  | rs3816769 | 37751799 |
|  |  | rs744166 | 37767727 |
| ***STX18*** | 4p16.3-p16.2 | rs2044 | 4539127 |
|  |  | rs10516164 | 4615083 |
|  |  | rs4689118 | 4618201 |
|  |  | rs10516165 | 4627826 |
|  |  | rs689484 | 4640687 |
| ***SULT1A1*** | 16p12.1-p11.2 | rs12445705 | 28517197 |
|  |  | rs1801030 | 28524986 |
|  |  | rs9282862 | 28525738 |
|  |  | rs1126446 | 28527421 |
| ***SUMO1*** | 2q33 | rs6714212 | 202882493 |
|  |  | rs4675272 | 202912235 |
|  |  | rs7599810 | 202914774 |
|  |  | rs6755690 | 202916099 |
|  |  | rs6709162 | 202924065 |
|  |  | rs3754931 | 202928907 |
| ***TBX1*** | 22q11 | rs2238777 | 18132782 |
|  |  | rs2238778 | 18132953 |
|  |  | rs737869 | 18133991 |
|  |  | rs4819522 | 18141336 |
|  |  | rs2073762 | 18146122 |
|  |  | rs1978060 | 18124079 |
|  |  | rs2301558 | 18126383 |
| ***TBX10*** | 11q13.1 | rs7124513 | 67152290 |
|  |  | rs1531514 | 67155438 |
|  |  | rs2514022 | 67158689 |
|  |  | rs2514027 | 67162878 |
| ***TBX15*** | 1p11.1 | rs2282322 | 119158043 |
| ***TBX21*** | 17q21.32 | rs4794063 | 43159493 |
|  |  | rs7502875 | 43178226 |
|  |  | rs11659069 | 43189274 |
| ***TBX22*** | Xq21.1 | rs195295 | 79083561 |
|  |  | rs195294 | 79087542 |
|  |  | rs195293 | 79089654 |
| ***TBX4*** | 17q21-q22 | rs1468566 | 56882992 |
|  |  | rs11867179 | 56888135 |
|  |  | rs3744448 | 56888650 |
|  |  | rs3744447 | 56889769 |
| ***TCF1*** | 12q24.31 | rs1169288 | 119879370 |
|  |  | rs1169292 | 119889198 |
|  |  | rs1169301 | 119894020 |
|  |  | rs2464195 | 119898195 |
|  |  | rs2257764 | 119909166 |
| ***TCF21*** | 6pter-qter | rs2327429 | 134251530 |
|  |  | rs229919 | 134271012 |
| ***TCOF1*** | 5q33.1 | rs2255796 | 149731539 |
|  |  | rs7713638 | 149739289 |
|  |  | rs2748220 | 149744486 |
|  |  | rs2569062 | 149758557 |
| ***TFAP2A*** | 6p24 | rs616879 | 10500486 |
|  |  | rs303055 | 10527462 |
|  |  | rs1621700 | 10528084 |
| ***TGFA*** | 2p13 | rs494537 | 70570771 |
|  |  | rs3732253 | 70587753 |
|  |  | rs1807968 | 70619916 |
|  |  | rs377122 | 70678680 |
| ***TGFB1*** | 19q13.2 | rs8105161 | 46531471 |
|  |  | rs4803455 | 46543349 |
|  |  | rs2241715 | 46548726 |
| ***TGFB2*** | 1q41 | rs6658835 | 214909390 |
|  |  | rs2027566 | 214946722 |
|  |  | rs2799090 | 214957224 |
|  |  | rs2799103 | 214972814 |
|  |  | rs2000220 | 214976031 |
|  |  | rs2796813 | 214977592 |
| ***TGFB3*** | 14q24 | rs989910 | 74158311 |
|  |  | rs2359991 | 75475877 |
|  |  | rs2284792 | 75513332 |
| ***TGFBR1*** | 9q22.33 | rs1888223 | 98944529 |
|  |  | rs7874221 | 98948623 |
|  |  | rs10739778 | 98955344 |
|  |  | rs334363 | 99008257 |
| ***TGFBR2*** | 3p24.1 | rs1835538 | 30630485 |
|  |  | rs4522809 | 30643688 |
|  |  | rs2043138 | 30651021 |
|  |  | rs1155708 | 30661744 |
|  |  | rs3773634 | 30672940 |
|  |  | rs876688 | 30700780 |
| ***TGFBR3*** | 1p33-p32 | rs11165354 | 91906343 |
|  |  | rs284180 | 91939869 |
|  |  | rs284185 | 91945841 |
|  |  | rs4658269 | 91959675 |
|  |  | rs10493859 | 91985665 |
|  |  | rs284149 | 91988556 |
|  |  | rs2129975 | 91994101 |
| ***TGIF*** | 18p11.3 | rs12457997 | 3405831 |
|  |  | rs7234567 | 3410806 |
|  |  | rs4798095 | 3429156 |
|  |  | rs2229333 | 3447607 |
| ***THRB*** | 3p24.3 | rs4858582 | 24194525 |
|  |  | rs1466120 | 24247294 |
|  |  | rs1667744 | 24271092 |
|  |  | rs1158265 | 24311166 |
|  |  | rs1394766 | 24337712 |
|  |  | rs9839986 | 24435352 |
|  |  | rs982953 | 24486257 |
| ***TIMP2*** | 17q25 | rs9900972 | 74380209 |
|  |  | rs7502935 | 74390224 |
|  |  | rs6501266 | 74418948 |
|  |  | rs8066695 | 74425438 |
|  |  | rs7212662 | 74429726 |
| ***TNFRSF10B*** | 8p22-p21 | rs2889 | 22931854 |
|  |  | rs883429 | 22942763 |
|  |  | rs4460370 | 22943553 |
|  |  | rs11785599 | 22948219 |
|  |  | rs11135693 | 22981099 |
|  |  | rs11778166 | 22985295 |
| ***TNNT3*** | 11p15.5 | rs965912 | 1900778 |
|  |  | rs643753 | 1906046 |
|  |  | rs2292473 | 1907482 |
|  |  | rs7395920 | 1920888 |
|  |  | rs1618613 | 1926464 |
| ***TP63*** | 3q27 | rs1920282 | 190874759 |
|  |  | rs6804480 | 190912003 |
|  |  | rs4687091 | 190931247 |
|  |  | rs6779677 | 190941806 |
|  |  | rs869546 | 190999799 |
|  |  | rs4687094 | 191011554 |
|  |  | rs9810322 | 191026676 |
|  |  | rs1515490 | 191079557 |
|  |  | rs4687100 | 191093728 |
| ***TRPS1*** | 8q24.12 | rs800897 | 116491112 |
|  |  | rs12334912 | 116516401 |
|  |  | rs3808409 | 116521516 |
|  |  | rs800888 | 116534165 |
|  |  | rs1180626 | 116545569 |
|  |  | rs1180624 | 116545987 |
|  |  | rs3779878 | 116625474 |
|  |  | rs3808451 | 116648263 |
|  |  | rs2737205 | 116679355 |
|  |  | rs2737219 | 116704863 |
|  |  | rs727582 | 116719643 |
| ***TULP3*** | 12p13.3 | rs2302283 | 2901619 |
|  |  | rs7316823 | 2923009 |
|  |  | rs10774085 | 2930485 |
| ***TWIST1*** | 7p21.2 | rs2285681 | 18932006 |
|  |  | rs1157350 | 18945840 |
| ***TYMS*** | 18p11.32 | rs502396 | 649236 |
|  |  | rs2244500 | 651005 |
|  |  | rs10502290 | 665903 |
|  |  | rs10502289 | 666789 |
| ***UFD1L*** | 22q11.21 | rs2073731 | 17812492 |
|  |  | rs2269722 | 17819469 |
|  |  | rs5748219 | 17825822 |
|  |  | rs737858 | 17846163 |
| ***UGT1A7*** | 2q37 | rs7577677 | 234372616 |
|  |  | rs4663888 | 234373987 |
|  |  | rs6724485 | 234374816 |
|  |  | rs10168416 | 234379087 |
|  |  | rs6725478 | 234397400 |
|  |  | rs7572563 | 234399236 |
|  |  | rs6744284 | 234407297 |
|  |  | rs1875263 | 234407622 |
|  |  | rs7597496 | 234412443 |
|  |  | rs7574296 | 234420249 |
| ***VCL*** | 10q22.1-q23 | rs10762573 | 75468154 |
|  |  | rs4268450 | 75488081 |
|  |  | rs2131960 | 75501265 |
|  |  | rs4746172 | 75525848 |
|  |  | rs6480716 | 75545800 |
| ***WHSC1*** | 4p16.3 | rs487903 | 1869140 |
|  |  | rs489015 | 1919532 |
|  |  | rs474235 | 1928368 |
| ***WNT3A*** | 1q42 | rs708121 | 224514992 |
|  |  | rs708122 | 224523732 |
|  |  | rs947631 | 224530282 |
| ***WNT4*** | 1p36.23-p35.1 | rs1474647 | 22187171 |
|  |  | rs3765351 | 22191297 |
|  |  | rs2235529 | 22195793 |
|  |  | rs7526484 | 22197151 |
|  |  | rs3820282 | 22213521 |
|  |  | rs4655026 | 22218964 |
|  |  | rs7542242 | 22222799 |
| ***WNT5A*** | 3p21 | rs7622120 | 55479770 |
|  |  | rs11918967 | 55480430 |
|  |  | rs556874 | 55482321 |
|  |  | rs472631 | 55486715 |
| ***WNT6*** | 2q35 | rs751135 | 219545854 |
|  |  | rs6754599 | 219557647 |
|  |  | rs3806557 | 219569379 |
|  |  | rs10177996 | 219572066 |
| ***WNT7B*** | 22q13 | rs133349 | 40753227 |
| ***WNT9B*** | 17q21 | rs2165846 | 42296365 |
|  |  | rs6504591 | 42299827 |
|  |  | rs4968281 | 42305121 |
|  |  | rs1530364 | 42306776 |
|  |  | rs1530365 | 42311711 |
| ***XPD*** | 19q13.3 | rs13181 | 50546759 |
|  |  | rs171140 | 50556842 |
|  |  | rs11878644 | 50568807 |
| ***XRCC1*** | 19q13.2 | rs25474 | 48741890 |
|  |  | rs1799782 | 48749414 |
|  |  | rs1799778 | 48750981 |
|  |  | rs25495 | 48757006 |
|  |  | rs1001581 | 48757228 |
| ***XRCC3*** | 14q32.3 | rs861545 | 103225698 |
|  |  | rs861531 | 103242560 |
|  |  | rs861528 | 103252751 |
| ***ZFHX1B*** | 2q22 | rs3770305 | 145002909 |
|  |  | rs6430057 | 145005644 |
|  |  | rs7600752 | 145011842 |
|  |  | rs1427297 | 145061190 |
|  |  | rs1365779 | 145072714 |
| ***ZIC3*** | Xq26.2 | rs5931172 | 136367469 |
|  |  | rs10856541 | 136376454 |
|  |  | rs5931174 | 136380666 |
| ***ZNF189*** | 9q22-q31 | rs2777336 | 101243717 |
|  |  | rs1929488 | 101245553 |
|  |  | rs2777345 | 101247273 |
|  |  | rs475348 | 101259033 |
|  |  | rs565474 | 101261115 |

^a^ All 1536 SNPs for the 357 selected candidate genes are listed in the table.

^b^ SNP coordinates are based on NCBI Human Genome Build 35.1.
