# Supplementary material for: Genetic Determinants of Facial Clefting: Analysis of 357 Candidate Genes Using Two National Cleft Studies from Scandinavia
Source: PLoS One. 2009 Apr 29;4(4):e5385. doi: 10.1371/journal.pone.0005385 (PMC2671138; doi:10.1371/journal.pone.0005385)
Supplement: Table S3 — TRIMM results for I-CL/P. (0.09 MB DOC) [file pone.0005385.s003.doc]

**Table S3.** TRIMM results for I-CL/P.

| Chromosome | Gene ID ^a^ | Number of SNPs | Norway I-CL/P p-value ^b^ | Denmark I-CL/P p-value ^b^ | Fisher-combined p-values ^b^ |
| --- | --- | --- | --- | --- | --- |
| 1 | ***IRF6*** | 7 | **0.0390** | **0.0005** | **0.0002** |
| 1 | *MTR* | 3 | 0.6170 | **0.0234** | 0.0756 |
| 1 | *TGFB2* | 6 | **0.0172** | 0.3248 | **0.0346** |
| 2 | *CYP1B1* | 6 | **0.0185** | 0.8225 | 0.0789 |
| 2 | *DLX1* | 3 | 0.5204 | **0.0011** | **0.0048** |
| 2 | *MTHFD2* | 2 | 0.9074 | **0.0216** | 0.0967 |
| 3 | *CTNNB1* | 4 | **0.0278** | 0.9506 | 0.1224 |
| 3 | *EVI1* | 5 | **0.0164** | 0.1793 | **0.0201** |
| 3 | *FGF12* | 6 | 0.0610 | **0.0202** | **0.0095** |
| 3 | *RYK* | 4 | **0.0446** | 0.2686 | 0.0650 |
| 3 | *TP63* | 9 | 0.6433 | **0.0453** | 0.1322 |
| 3 | *WNT5A* | 3 | **0.0233** | 0.9958 | 0.1105 |
| 4 | *ADH1B* | 3 | **0.0227** | 0.2127 | **0.0306** |
| 4 | ***ADH1C*** | 3 | **0.0285** | **0.0054** | **0.0015** |
| 4 | *ADH4* | 5 | 0.3520 | **0.0384** | 0.0717 |
| 4 | *ADH5* | 4 | **0.0286** | 0.1140 | **0.0219** |
| 4 | *FGFR3* | 2 | 0.4870 | **0.0020** | **0.0077** |
| 4 | *MSX1* | 5 | **0.0093** | 0.5440 | **0.0318** |
| 4 | *PDGFRA* | 4 | **0.0184** | 0.1471 | **0.0187** |
| 6 | *DSP* | 5 | **0.0336** | 0.5257 | 0.0890 |
| 7 | *TWIST1* | 2 | 0.6872 | **0.0221** | 0.0788 |
| 8 | *NAT1* | 4 | 0.0688 | 0.0648 | **0.0286** |
| 9 | *BARX1* | 3 | **0.0304** | 0.8823 | 0.1239 |
| 9 | *FOXE1* | 7 | 0.0928 | **0.0035** | **0.0029** |
| 10 | *VCL* | 5 | **0.0351** | 0.5486 | 0.0953 |
| 11 | *APOA5* | 2 | **0.0025** | 0.9964 | **0.0174** |
| 11 | *DHCR7* | 2 | **0.0367** | 0.2881 | 0.0587 |
| 11 | *FOLR3* | 3 | 0.3582 | **0.0350** | 0.0674 |
| 11 | *TNNT3* | 5 | 0.0706 | **0.0082** | **0.0049** |
| 12 | *PTPN11* | 4 | **0.0470** | 0.7507 | 0.1533 |
| 12 | *SOX5* | 9 | **0.0299** | 0.1533 | **0.0293** |
| 14 | *CHES1* | 8 | 0.9674 | **0.0425** | 0.1723 |
| 14 | *JAG2* | 4 | **0.0182** | 0.9674 | 0.0887 |
| 14 | *XRCC3* | 2 | **0.0392** | 0.5549 | 0.1050 |
| 17 | *FZD2* | 2 | 0.2171 | **0.0170** | **0.0244** |
| 17 | *HOXB6* | 3 | **0.0284** | 0.7979 | 0.1085 |
| 17 | *TBX21* | 3 | **0.0396** | 0.9819 | 0.1651 |
| 19 | *XRCC1* | 3 | **0.0227** | 0.0657 | **0.0112** |
| 20 | *AHCY* | 2 | **0.0405** | 0.7169 | 0.1318 |
| 20 | *JAG1* | 4 | **0.0194** | 0.6458 | 0.0674 |
| 22 | *CYP2D6* | 4 | **0.0026** | 0.7255 | **0.0137** |

^a^ Gene ID from NCBI Entrez Gene. Genes associated in both samples are boldfaced.

^b^ P-values ≤ 0.05 are boldfaced (the Fisher-combined p-values have not been Bonferroni-corrected).
