# Supplementary material for: Genetic Determinants of Facial Clefting: Analysis of 357 Candidate Genes Using Two National Cleft Studies from Scandinavia
Source: PLoS One. 2009 Apr 29;4(4):e5385. doi: 10.1371/journal.pone.0005385 (PMC2671138; doi:10.1371/journal.pone.0005385)
Supplement: Table S4 — TRIMM results for I-CP. (0.09 MB DOC) [file pone.0005385.s004.doc]

**Table S4.** TRIMM results for I-CP.

| Chromosome | Gene ID ^a^ | Number of SNPs | Norway I-CP p-value ^b^ | Denmark I-CP p-value ^b^ | Fisher-combined p-values ^b^ |
| --- | --- | --- | --- | --- | --- |
| 1 | ***ALX3*** | 4 | **0.0336** | **0.0387** | **0.0099** |
| 1 | *LHX8* | 4 | **0.0266** | 0.1924 | **0.0321** |
| 1 | *PKP1* | 5 | **0.0368** | 0.3465 | 0.0684 |
| 1 | *PTCH2* | 2 | 0.9072 | **0.0279** | 0.1184 |
| 1 | *WNT3A* | 3 | **0.0458** | 0.1044 | **0.0303** |
| 2 | *BMP10* | 3 | 0.8340 | **0.0204** | 0.0863 |
| 4 | *FGF5* | 2 | 0.8576 | **0.0014** | **0.0093** |
| 4 | *FGFBP1* | 2 | 0.3392 | **0.0264** | 0.0512 |
| 4 | ***PDGFC*** | 5 | **0.0237** | **0.0411** | **0.0077** |
| 4 | *PITX2* | 3 | 0.0850 | 0.0697 | **0.0363** |
| 5 | *NIPBL* | 2 | **0.0348** | 0.6752 | 0.1116 |
| 5 | *NR3C1* | 6 | **0.0451** | 0.1943 | 0.0503 |
| 5 | *TCOF1* | 3 | **0.0498** | 0.1557 | **0.0454** |
| 7 | *AHR* | 4 | **0.0206** | 0.8289 | 0.0866 |
| 7 | *CYP3A7* | 2 | **0.0166** | 0.3403 | **0.0349** |
| 7 | *SPAM1* | 1 | 0.4780 | **0.0220** | 0.0584 |
| 8 | *FOXH1* | 3 | **0.0056** | 0.4212 | **0.0166** |
| 8 | *RECQL4* | 3 | **0.0415** | 0.2398 | 0.0558 |
| 9 | *BARX1* | 3 | **0.0254** | 0.5959 | 0.0786 |
| 10 | *EMX2* | 3 | 0.3648 | **0.0189** | **0.0412** |
| 10 | *FZD8* | 2 | **0.0132** | 0.9325 | 0.0664 |
| 10 | ***MKX*** | 4 | **0.0355** | **0.0172** | **0.0051** |
| 11 | *FOLRB* | 2 | **0.0093** | 0.6748 | **0.0381** |
| 12 | *PTPN11* | 4 | 0.9160 | **0.0099** | 0.0517 |
| 12 | *SHMT2* | 1 | **0.0430** | 0.8825 | 0.1621 |
| 13 | *FGF9* | 6 | **0.0050** | 0.5973 | **0.0203** |
| 14 | *ESRRB* | 4 | 0.9526 | **0.0111** | 0.0587 |
| 14 | *IRF9* | 3 | 0.4885 | **0.0270** | 0.0703 |
| 14 | *JAG2* | 4 | 0.4069 | **0.0331** | 0.0715 |
| 16 | *ERCC4* | 3 | 0.8215 | **0.0213** | 0.0883 |
| 17 | *FZD2* | 2 | **0.0053** | 0.9649 | **0.0321** |
| 17 | *RARA* | 2 | 0.9303 | **0.0052** | **0.0306** |
| 17 | *STAT3* | 2 | 0.1919 | **0.0470** | 0.0515 |
| 18 | *TGIF* | 4 | 0.2806 | **0.0023** | **0.0054** |
| 20 | *CHRNA4* | 6 | **0.0095** | 0.7704 | **0.0433** |

^a^ Gene ID from NCBI Entrez Gene. Genes associated in both samples are boldfaced.

^b^ P-values ≤ 0.05 are boldfaced (the Fisher-combined p-values have not been Bonferroni-corrected).
