# Supplementary material for: Genetic Determinants of Facial Clefting: Analysis of 357 Candidate Genes Using Two National Cleft Studies from Scandinavia
Source: PLoS One. 2009 Apr 29;4(4):e5385. doi: 10.1371/journal.pone.0005385 (PMC2671138; doi:10.1371/journal.pone.0005385)
Supplement: Table S5 — HAPLIN results for I-CL/P. (0.08 MB DOC) [file pone.0005385.s005.doc]

**Table S5.** HAPLIN results for I-CL/P.

| Chromosome | Gene ID ^a^ | Number of SNPs | Norway I-CL/P p-value ^b^ | Denmark I-CL/P p-value ^b^ | Fisher-combined p-values ^b^ |
| --- | --- | --- | --- | --- | --- |
| 1 | ***IRF6*** | 7 | **0.0134** | **0.0004** | **0.0001** |
| 2 | *DLX1* | 3 | 0.5517 | **0.0187** | 0.0576 |
| 2 | *GLI2* | 2 | 0.0762 | 0.0631 | **0.0305** |
| 2 | *MTHFD2* | 2 | 0.6058 | **0.0002** | **0.0013** |
| 2 | *PAX3* | 5 | **0.0023** | 0.9213 | **0.0153** |
| 2 | *SUMO1* | 6 | **0.0304** | 0.2890 | 0.0504 |
| 2 | *TGFA* | 4 | 0.3826 | **0.0170** | **0.0393** |
| 2 | *ZFHX1B* | 5 | **0.0480** | 0.0769 | **0.0244** |
| 3 | *CTNNB1* | 4 | **0.0260** | 0.8438 | 0.1058 |
| 3 | ***FGF12*** | 6 | **0.0113** | **0.0415** | **0.0041** |
| 3 | *WNT5A* | 3 | **0.0349** | 0.6963 | 0.1147 |
| 4 | *ADH1B* | 3 | **0.0277** | 0.5120 | 0.0745 |
| 4 | ***ADH1C*** | 3 | **0.0393** | **0.0463** | **0.0133** |
| 4 | *FGFR3* | 2 | 0.2052 | **0.0134** | **0.0189** |
| 4 | *MSX1* | 5 | **0.0258** | 0.2676 | **0.0413** |
| 4 | *PDGFRA* | 4 | **0.0127** | 0.4534 | **0.0354** |
| 5 | *FGF10* | 5 | **0.0145** | 0.1617 | **0.0165** |
| 6 | *CX43* | 2 | 0.3777 | **0.0105** | **0.0259** |
| 7 | *MDR1* | 3 | 0.6956 | **0.0394** | 0.1260 |
| 9 | *BARX1* | 3 | **0.0384** | 0.5968 | 0.1095 |
| 9 | *FOXE1* | 7 | 0.0998 | **0.0148** | **0.0111** |
| 9 | *LMX1B* | 3 | **0.0169** | 0.7973 | 0.0714 |
| 9 | *PTCH1* | 2 | **0.0335** | 0.7799 | 0.1214 |
| 10 | *VCL* | 5 | **0.0018** | 0.9177 | **0.0119** |
| 11 | *APOA5* | 2 | **0.0016** | 0.7869 | **0.0099** |
| 12 | *PTPN11* | 4 | **0.0275** | 0.2427 | **0.0401** |
| 14 | *JAG2* | 4 | **0.0050** | 0.7754 | **0.0256** |
| 14 | *XRCC3* | 2 | **0.0392** | 0.6340 | 0.1168 |
| 17 | *HOXB6* | 3 | **0.0463** | 0.9664 | 0.1837 |
| 17 | *TBX21* | 3 | **0.0170** | 0.8768 | 0.0775 |
| 17 | *TBX4* | 3 | **0.0084** | 0.9090 | **0.0451** |
| 17 | *TIMP2* | 5 | **0.0205** | 0.8404 | 0.0872 |
| 18 | *TGIF* | 4 | 0.1970 | **0.0478** | 0.0533 |
| 19 | *XRCC1* | 3 | 0.1820 | **0.0215** | **0.0256** |
| 20 | *BMP2* | 4 | 0.9208 | **0.0236** | 0.1050 |
| 22 | *TBX1* | 6 | **0.0240** | 0.2549 | **0.0373** |
| 22 | *UFD1L* | 3 | **0.0115** | 0.0760 | **0.0070** |

^a^ Gene ID from NCBI Entrez Gene. Genes associated in both samples are boldfaced.

^b^ P-values ≤ 0.05 are boldfaced (the Fisher-combined p-values have not been Bonferroni-corrected).
