# Supplementary material for: Genetic Determinants of Facial Clefting: Analysis of 357 Candidate Genes Using Two National Cleft Studies from Scandinavia
Source: PLoS One. 2009 Apr 29;4(4):e5385. doi: 10.1371/journal.pone.0005385 (PMC2671138; doi:10.1371/journal.pone.0005385)
Supplement: Table S6 — HAPLIN results for I-CP. (0.08 MB DOC) [file pone.0005385.s006.doc]

**Table S6**. HAPLIN results for I-CP.

| Chromosome | Gene ID ^a^ | Number of SNPs | Norway I-CP p-value ^b^ | Denmark I-CP p-value ^b^ | Fisher-combined p-values ^b^ |
| --- | --- | --- | --- | --- | --- |
| 1 | *DVL1* | 1 | 0.7309 | **0.0300** | 0.1056 |
| 1 | *LHX8* | 4 | **0.0168** | 0.2965 | **0.0314** |
| 1 | *PKP1* | 5 | **0.0142** | 0.8783 | 0.0670 |
| 1 | *PTCH2* | 2 | 0.9899 | **0.0483** | 0.1931 |
| 1 | *TGFBR3* | 7 | 0.6692 | **0.0445** | 0.1345 |
| 1 | *WNT3A* | 3 | **0.0191** | 0.1649 | **0.0213** |
| 3 | *CRELD1* | 2 | 0.8504 | **0.0308** | 0.1217 |
| 3 | ***ETV5*** | 5 | **0.0134** | **0.0340** | **0.0040** |
| 3 | *FLNB* | 5 | **0.0204** | 0.9087 | 0.0926 |
| 4 | *FGF5* | 2 | 0.9241 | **0.0446** | 0.1727 |
| 4 | *LEF1* | 5 | 0.4049 | **0.0191** | **0.0454** |
| 4 | ***PDGFC*** | 5 | **0.0389** | **0.0317** | **0.0095** |
| 5 | *CORS26* | 3 | 0.9281 | **0.0371** | 0.1505 |
| 5 | *DMGDH* | 8 | 0.7183 | **0.0373** | 0.1238 |
| 5 | *MTRR* | 5 | 0.8121 | **0.0317** | 0.1200 |
| 5 | *NIPBL* | 2 | **0.0300** | 0.8219 | 0.1161 |
| 5 | *TCOF1* | 3 | **0.0289** | 0.1707 | **0.0311** |
| 6 | *COL11A2* | 6 | **0.0334** | 0.3270 | 0.0603 |
| 7 | *CYP3A7* | 2 | **0.0357** | 0.8534 | 0.1369 |
| 7 | *FZD1* | 4 | 0.7352 | **0.0360** | 0.1226 |
| 8 | *FOXH1* | 3 | **0.0263** | 0.7936 | 0.1015 |
| 8 | *MSC* | 4 | 0.0500 | 0.5869 | 0.1328 |
| 8 | *TRPS1* | 10 | **0.0280** | 0.2017 | **0.0349** |
| 9 | *FOXE1* | 7 | **0.0055** | 0.4091 | **0.0160** |
| 9 | *LMX1B* | 3 | 0.6992 | **0.0230** | 0.0826 |
| 9 | *TGFBR1* | 3 | 0.7109 | **0.0266** | 0.0938 |
| 10 | *SARA1* | 3 | 0.5681 | **0.0015** | **0.0070** |
| 11 | *FOLRB* | 2 | **0.0136** | 0.3608 | **0.0310** |
| 12 | *SPPL3* | 5 | 0.4640 | **0.0269** | 0.0672 |
| 13 | *FGF9* | 6 | **0.0038** | 0.5539 | **0.0150** |
| 15 | *MTHFS* | 3 | 0.1449 | **0.0409** | **0.0363** |
| 16 | *SALL1* | 3 | 0.5277 | **0.0291** | 0.0794 |
| 17 | *FZD2* | 2 | **0.0033** | 0.9332 | **0.0207** |
| 17 | *RARA* | 2 | 0.4342 | **0.0239** | 0.0579 |
| 17 | *SOX9* | 5 | **0.0450** | 0.6353 | 0.1302 |
| 17 | *STAT3* | 2 | 0.1345 | **0.0491** | **0.0397** |
| 20 | *CHRNA4* | 6 | **0.0199** | 0.3690 | **0.0434** |
| 20 | *SNAI1* | 5 | **0.0342** | 0.4698 | 0.0824 |
| 21 | *GART* | 6 | 0.6528 | **0.0381** | 0.1168 |

^a^ Gene ID from NCBI Entrez Gene. Genes associated in both samples are boldfaced.

^b^ P-values ≤ 0.05 are boldfaced (the Fisher-combined p-values have not been Bonferroni-corrected).
